# Risk factors for stomach cancer: a systematic review and meta-analysis

**DOI:** 10.4178/epih.e2020004

**Published:** 2020-02-02

**Authors:** Jalal Poorolajal, Leila Moradi, Younes Mohammadi, Zahra Cheraghi, Fatemeh Gohari-Ensaf

**Affiliations:** 1 Department of Epidemiology, School of Public Health, Hamadan University of Medical Sciences, Hamadan, Iran; 2 Research Center for Health Sciences, Hamadan University of Medical Sciences, Hamadan, Iran; 3 Modeling of Noncommunicable Diseases Research Center, Hamadan University of Medical Sciences, Hamadan, Iran; 4 Social Determinants of Health Research Center, Hamadan University of Medical Sciences, Hamadan, Iran

**Keywords:** Stomach neoplasms, Gastric neoplasms, Risk factors, Behavior, Nutrition status, Meta-analysis

## Abstract

**OBJECTIVES:**

This report provides information on 14 behavioral and nutritional factors that can be addressed in stomach cancer prevention programs.

**METHODS:**

PubMed, Web of Science, and Scopus were searched through December 2018. Reference lists were also screened. Observational studies addressing the associations between stomach cancer and behavioral factors were analyzed. Between-study heterogeneity was investigated using the χ^2^, τ^2^, and I^2^ statistics. The likelihood of publication bias was explored using the Begg and Egger tests and trim-and-fill analysis. Effect sizes were expressed as odds ratios (ORs) with 95% confidence intervals (CIs) using a random-effects model.

**RESULTS:**

Of 52,916 identified studies, 232 (including 33,831,063 participants) were eligible. The OR (95% CI) of factors associated with stomach cancer were as follows: *Helicobacter pylori* infection, 2.56 (95% CI, 2.18 to 3.00); current smoking, 1.61 (95% CI, 1.49 to 1.75); former smoking 1.43 (95% CI, 1.29 to 1.59); current drinking, 1.19 (95% CI, 1.10 to 1.29); former drinking, 1.73 (95% CI, 1.17 to 2.56); overweight/obesity, 0.89 (95% CI, 0.74 to 1.08); sufficient physical activity, 0.83 (95% CI, 0.68 to 1.02); consumption of fruits ≥3 times/wk, 0.48 (95% CI, 0.37 to 0.63); consumption of vegetables ≥3 times/wk, 0.62 (95% CI, 0.49 to 0.79); eating pickled vegetables, 1.28 (95% CI, 1.09 to 1.51); drinking black tea, 1.00 (95% CI, 0.84 to 1.20); drinking green tea, 0.88 (95% CI, 0.80 to 0.97); drinking coffee, 0.99 (95% CI, 0.88 to 1.11); eating fish ≥1 time/wk 0.79 (95% CI, 0.61 to 1.03); eating red meat ≥4 times/wk 1.31 (95% CI, 0.87 to 1.96), and high salt intake 3.78 (95% CI, 1.74 to 5.44) and 1.34 (95% CI, 0.88 to 2.03), based on two different studies.

**CONCLUSIONS:**

This meta-analysis provided a clear picture of the behavioral and nutritional factors associated with the development of stomach cancer. These results may be utilized for ranking and prioritizing preventable risk factors to implement effective prevention programs.

## INTRODUCTION

Stomach cancer, also known as gastric cancer, is the fifth most frequent type of cancer and the third-leading cause of cancer-related death worldwide, responsible for over 1,000,000 new cases and an estimated 783,000 deaths in 2018 [1].

Many factors may play a role in the development of stomach cancer. Advanced age [2], male sex [1], ethnicity [3], and genetic factors [4] may contribute to the development of stomach cancer, but they are neither modifiable nor preventable. However, nutritional factors [5] and behavioral factors such as cigarette smoking [6] and drinking alcohol [6,7], as well as *Helicobacter pylori* infection [8], also contribute to the development of stomach cancer. These factors are largely modifiable and preventable, and therefore can be considered when designing effective prevention programs.

Efforts to improve screening programs and the early detection and treatment of stomach cancer are important, but taking action to address preventable factors that play a role in the development of stomach cancer is a priority. Ranking and prioritizing the factors that contribute to stomach cancer and implementing prevention programs can prevent thousands of cases of stomach cancer each year. Effective intervention strategies and prevention programs require a comprehensive understanding and a clear picture of the factors that promote stomach cancer. No comprehensive systematic review has yet been conducted to address all the potential behavioral and nutritional factors that play a pivotal role in the development of stomach cancer. This systematic review was conducted to address the associations between stomach cancer and 14 potentially modifiable behavioral and nutritional factors that may be addressed in prevention programs aimed at reducing the incidence of stomach cancer.

## MATERIALS AND METHODS

### Eligibility criteria

The outcome of interest was pathologically confirmed stomach cancer, of any type (adenocarcinoma, lymphoma, sarcoma, or carcinoid) and location (cardia or non-cardia), among the general population, regardless of age, sex, race, ethnicity, and geographical region. The exposures of interest are listed below:

*H. pylori* infection, regardless of cytotoxin-associated gene A (CagA) pathogenicity (positive vs. negative); Cigarette smoking (current/former smokers vs. non-smokers); Drinking alcohol (current/former drinkers vs. non-drinkers); Body mass index (BMI; overweight/obese vs. normal weight); Physical activity (sufficient vs. insufficient); Fruit consumption (≥7 times/wk vs. <7 times/wk and ≥3 times/wk vs. <3 times/wk); Vegetable consumption (≥7 times/wk vs. <7 times/wk and ≥3 times/wk vs. <3 times/wk); Consumption of pickled vegetables (yes vs. no); Drinking black tea (yes vs. no); Drinking green tea (yes vs. no); Drinking coffee (yes vs. no); Fish consumption (≥1 serving/wk vs. <1 serving/wk); Red meat consumption (≥4 times/wk vs. <4 times/wk); Salt intake (>5 g/d vs. ≤5 g/d); A BMI of 18.5-24.9 kg/m^2^ was classified as normal weight, 25.0-29.9 kg/m^2^ as overweight, and ≥30.0 kg/m^2^ as obese.

At least 60 minutes of moderate- to vigorous-intensity physical activity per day (or 300 min/wk) was considered sufficient for adults [9].

Observational (cohort and case-control) studies addressing the association between stomach cancer and any of the above factors were included in the meta-analysis, irrespective of language, publication date, and the nationality, race, sex, and age of participants.

### Information sources and search

PubMed, Web of Science, and Scopus were searched through December 2018. The reference lists of the included studies were also explored. The following terms were searched: (stomach cancer OR gastric cancer OR stomach neoplasms OR gastric neoplasms OR gastric malignancy OR stomach malignancy OR stomach tumor OR gastric tumor) AND (*Helicobacter pylori* OR *H. pylori* OR smoking OR cigarette OR tobacco products OR tobacco OR alcohol OR ethanol OR body mass index OR BMI OR overweight OR obesity OR obese OR physical activity OR exercise OR fruit OR vegetable OR pickled OR meat OR coffee OR tea OR fish OR salt OR sodium chloride).

### Study selection

The search results of all databases were combined using EndNote, and duplicates were deleted. Then, 2 authors (LM and FG) independently screened the titles and abstracts and excluded ineligible studies. The full texts of potentially relevant studies were retrieved for further evaluation.

### Data extraction

The data from the relevant studies were extracted by 2 authors (LM and JP) using an electronic data collection form prepared in Stata (StataCorp., College Station, TX, USA).

### Methodological quality

The Newcastle-Ottawa Scale (NOS) [10] was used to assess the methodological quality of the included studies. Based on this scale, a maximum of 9 stars was assigned to each study. Studies that received 7 or more stars were labeled high-quality, and otherwise studies were classified as low-quality.

### Heterogeneity and publication bias

The heterogeneity across studies was examined using the chi-square (χ^2^) test [11] and tau-square (τ^2^) test and was quantified by the I^2^ statistic [12]. According to the I^2^ value, heterogeneity was classified as low (<50%), moderate (50-74%), or high (≥75%).

The possibility of publication bias was explored by the Egger [13] and Begg [14] tests and the trim-and-fill method [15].

### Summary measures

The effect measure of choice was the odds ratio (OR), rate ratio, or hazard ratio with 95% confidence intervals (CIs). However, we analyzed these effect measures separately.

The results were reported based on a random-effects model [16]. The data were analyzed at a significance level of 0.05 using Stata version 14.2 (StataCorp., College Station, TX, USA) and Review Manager version 5.3 (https://review-manager.software.informer.com/5.3/).

### Sensitivity analysis

If the between-study heterogeneity was moderate to high (I^2^ ≥50%), the source of heterogeneity was investigated using a sequential algorithm [17].

### Ethics statement

This study was a systematic review in which no human subject or animal was employed.

## RESULTS

### Description of studies

**Figure 1.**
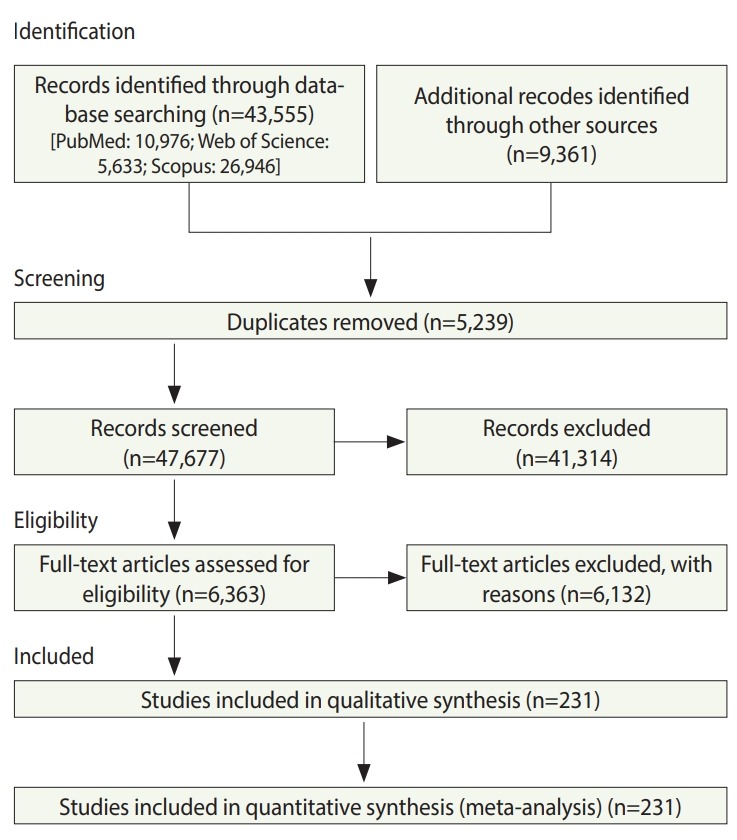
Flow of information through the various phases of the systematic review.

In total, 52,916 studies were identified, including 43,555 studies obtained by searching the electronic databases through December 2018 and 9,359 articles identified by searching the reference lists of the included studies. After excluding duplicates and ineligible studies, 232 studies with 33,831,063 participants (Supplementary Material 1) were included in the meta-analysis (Figure 1).

### Synthesis of results

#### *H. pylori* infection

Based on 68 studies (Supplementary Material 2), the overall OR for positive versus negative *H. pylori* infection status was 2.56 (95% CI, 2.18 to 3.00). The overall effect measure showed that *H. pylori* infection significantly increased the risk of stomach cancer by more than 2.5-fold (p=0.001). Between-study heterogeneity was high (I^2^=86%). The overall effect became weaker (OR, 2.13; 95% CI, 1.89 to 2.41; I^2^=47%) after performing a sensitivity analysis (Table 1).

**Figure 2.**
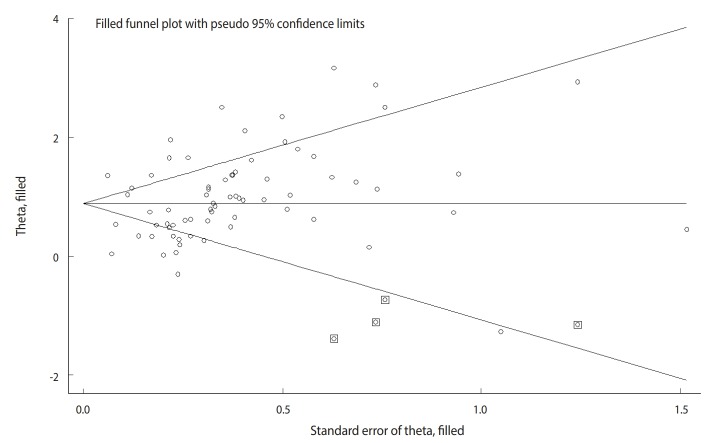
Trim-and-fill analysis estimating the number of possible missing studies for the association between stomach cancer and *Helicobacter pylori* infection. The squares represent the possible missing studies.

The Begg test (p=0.004), but not the Egger test (p=0.122), revealed evidence of publication bias. Trim-and-fill analysis estimated 4 missing studies (Figure 2). The overall effect measure based on this analysis was an OR of 2.42 (95% CI, 2.06 to 2.83), which was slightly weaker than the originally reported overall effect measure.

#### Cigarette smoking

Based on 77 studies (Supplementary Material 3), the overall OR for current smokers versus never smokers was 1.61 (95% CI, 1.49 to 1.75). The overall effect measure showed that current smoking significantly increased the risk of stomach cancer by 61% (p=0.001). Between-study heterogeneity was high (I^2^=78%). The overall effect became slightly stronger (OR, 1.66; 95% CI, 1.54 to 1.79; I^2^=49%) after performing a sensitivity analysis (Table 1).

In addition, based on 66 studies (Supplemental Material 4), the overall OR for former smokers versus never smokers was 1.43 (95% CI, 1.29 to 1.59). The overall effect measure showed that former smoking significantly increased the risk of stomach cancer by 43% (p=0.001). Between-study heterogeneity was moderate (I^2^=65%). The overall effect became slightly weaker (OR, 1.35; 95% CI, 1.24 to 1.47; I^2^=44%) after performing a sensitivity analysis (Table 1).

The Begg test revealed no evidence of publication bias (p=0.722), but the Egger test did show evidence of publication bias (p=0.001). Trim-and-fill analysis estimated 19 missing studies, but the overall effect measure did not change significantly.

#### Drinking alcohol

Based on 84 studies (Supplementary Material 5), the overall OR for current drinkers versus never drinkers was 1.19 (95% CI, 1.10, 1.29). The overall effect measure showed that current drinking significantly increased the risk of stomach cancer by 19% (p= 0.001). Between-study heterogeneity was high (I^2^=83%). The overall effect became slightly weaker (OR, 1.05; 95% CI, 0.99 to 1.11; I^2^=50%) after performing a sensitivity analysis (Table 1).

In addition, based on 16 studies (Supplementary Material 6), the overall OR for former drinking versus never drinking was 1.73 (95% CI, 1.17 to 2.56). The overall effect measure showed that former drinking significantly increased the risk of stomach cancer by 73% (p=0.004). Between-study heterogeneity was high (I^2^= 84%). The overall effect became slightly weaker (OR, 2.01; 95% CI, 1.48 to 2.72; I^2^=48%) after performing a sensitivity analysis (Table 1). There was no evidence of publication bias.

#### Body mass index

Based on 25 studies (Supplementary Material 7), the overall OR for overweight/obesity versus normal weight was 0.89 (95% CI, 0.74 to 1.08). The overall effect measure showed that overweight/obesity had no significant effect on stomach cancer (p=0.240). Between-study heterogeneity was high (I^2^=86%). The overall effect changed slightly (OR, 1.14; 95% CI, 1.03 to 1.26; I^2^=41%) after performing a sensitivity analysis (Table 1). There was no evidence of publication bias.

#### Sufficient physical activity

Based on 11 studies (Supplementary Material 8), the overall OR for sufficient versus insufficient physical activity was 0.83 (95% CI, 0.68 to 1.02). The overall effect measure showed that physical activity had no significant effect on stomach cancer (p=0.080), which seems negligible. Between-study heterogeneity was low (I^2^=45%). There was no evidence of publication bias.

#### Fruits

Based on 13 studies (Supplementary Material 9), the overall OR for fruit consumption ≥3 times/wk versus fruit consumption <3 times/wk was 0.48 (95% CI, 0.37 to 0.63). The overall effect measure showed that fruit consumption significantly reduced the risk of stomach cancer by 48% (p=0.001). Between-study heterogeneity was high (I^2^=86%). The overall effect became slightly weaker (OR, 0.64; 95% CI, 0.55 to 0.75; I^2^=42%) after performing a sensitivity analysis (Table 1). Both the Begg test (p=0.010) and the Egger test (p=0.001) revealed evidence of publication bias, but trim-and-fill analysis did not change the results.

#### Vegetables

Based on 18 studies (Supplementary Material 10), the OR for vegetable consumption ≥3 times/wk versus vegetable consumption <3 times/wk was 0.62 (95% CI, 0.49 to 0.79). The overall effect measure showed that vegetable consumption significantly reduced the risk of stomach cancer by 62% (p=0.001). Between-study heterogeneity was high (I^2^=74%). The overall effect became slightly weaker (OR, 0.70; 95% CI, 0.58 to 0.84; I^2^=40%) after performing a sensitivity analysis (Table 1). There was no evidence of publication bias.

#### Pickled vegetable

Based on 19 studies (Supplementary Material 11), the overall OR for consuming versus not consuming pickled vegetables was 1.28 (95% CI, 1.09 to 1.51). The overall effect measure showed that consuming pickled vegetables significantly increased the risk of stomach cancer by 28% (p=0.001). Between-study heterogeneity was low (I^2^=39%). There was no evidence of publication bias.

#### Black tea

Based on 15 studies (Supplementary Material 12), the overall OR for drinking versus not drinking black tea was 1.00 (95% CI, 0.84 to 1.20). The overall effect measure showed that drinking black tea had no significant effect on stomach cancer (p=0.970). Between-study heterogeneity was moderate (I^2^=62%). The overall effect became slightly stronger (OR, 0.94; 95% CI, 0.83 to 1.07; I^2^=34%) after performing a sensitivity analysis (Table 1). No evidence of publication bias was revealed.

#### Green tea

Based on 16 studies (Supplementary Material 13), the overall OR for drinking versus not drinking green tea was 0.88 (95% CI, 0.80 to 0.97). The overall effect measure showed that drinking green tea had no significant effect on stomach cancer (p=0.010). Between-study heterogeneity was low (I^2^=22%). No evidence of publication bias was seen.

#### Coffee

Based on 14 studies (Supplementary Material 14), the overall OR for drinking coffee versus not drinking coffee was 0.99 (95% CI, 0.88 to 1.11). The overall effect measure showed that coffee drinking had no significant effect on stomach cancer (p=0.820). Between-study heterogeneity was moderate (I^2^=29%). There was no evidence of publication bias.

#### Fish

Based on 11 studies (Supplementary Material 15), the OR for eating fish ≥1 time/wk versus <1 time/wk was 0.79 (95% CI, 0.61 to 1.03). The overall effect showed that fish consumption had no significant effect on stomach cancer (p=0.080). Between-study heterogeneity was high (I^2^=76%). The overall effect became stronger (OR, 0.68; 95% CI, 0.55 to 0.83; I^2^=45%) after performing a sensitivity analysis (Table 1). No evidence of publication bias was seen.

#### Red meat

Based on 11 studies (Supplementary Material 16), the overall OR for eating red meat ≥4 times/wk versus <4 times/wk was 1.31 (95% CI, 0.87 to 1.96). The overall effect measure showed that consumption of red meat had no significant effect on stomach cancer (p=0.080). Between-study heterogeneity was high (I^2^=83%). The overall effect changed slightly (OR, 0.91; 95% CI, 0.77 to 1.09; I^2^= 11%) after performing a sensitivity analysis (Table 1). There was no evidence of publication bias.

#### Salt

Only 2 studies addressed the association between high salt intake and stomach cancer. The results of the two studies are reported separately rather than a pooled OR because of the number of studies was limited. According to these studies, the OR for salt intake of >5 g/d versus ≤5 g/d was 3.78 (95% CI, 1.74 to 5.44) [18] and 1.34 (95% CI, 0.88 to 2.03) [19], respectively. Both studies reported that a high intake of salt significantly increased the risk of stomach cancer.

**Figure 3.**
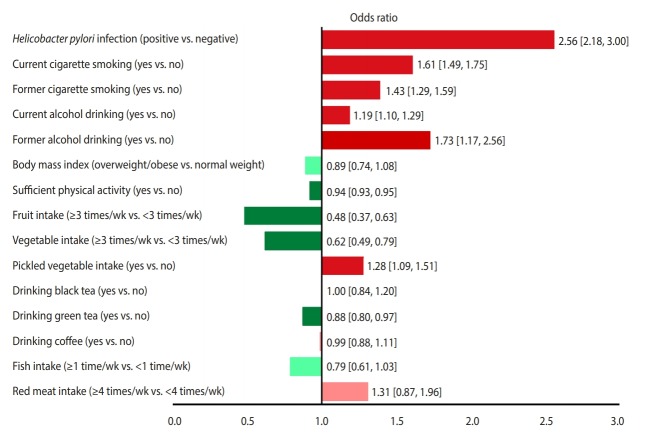
The associations (95% confidence intervals) between stomach cancer and nutritional and behavioral factors in a single view. Protective factors are shown in green (dark green, significant; light green, non-significant) and risk factors are shown in red (dark red, significant; light red, non-significant).

Figure 3 presents a unified overview of the associations between stomach cancer and all nutritional and behavioral factors. As shown in this figure, *H. pylori* infection, current and former cigarette smoking, current and former alcohol drinking, and pickled vegetable consumption were found to significantly increase the risk of stomach cancer. In contrast, sufficient physical activity, fruit consumption, and vegetable consumption significantly reduced the risk of stomach cancer. Meanwhile, BMI, drinking black tea, green tea, and coffee, and eating fish and red meat had no statistically significant effects on the risk of stomach cancer.

## DISCUSSION

According to our findings, *H. pylori* infection and smoking were the first and second most powerful risk factors for stomach cancer, respectively, whereas fruit and vegetable consumption were the first and second most powerful protective factors against stomach cancer, respectively.

The magnitudes of the measures of association reported in this systematic review may be used for ranking and prioritizing the relative importance of risk and protective factors. However, it should be kept in mind that these factors vary in terms of their physiological modus operandi and their units of exposure. Therefore, direct comparisons are often unwarranted [20]. In other words, the mere fact that the ORs of some risk factors for stomach cancer are higher than the ORs of other risk factors is not a sufficient basis for ranking and prioritizing risk factors. Instead, the prevalence of risk factors in the community is an essential criterion that must be taken into account when ranking and prioritizing risk factors. When the association between a particular risk factor and the outcome of interest is strong (a high OR), but the prevalence of that risk factor is low in the community, the overall impact of the risk factor on the disease burden in the community is low. In contrast, when a particular risk factor is common in the community, the overall impact of the factor on the outcome of interest may be tremendous even if the association between the risk factor and the outcome is not as strong (a low OR). Therefore, ranking and prioritizing the behavioral and nutritional factors affecting stomach cancer risk depends on both the strength of the associations (the magnitude of ORs) and the prevalence of the factors in the community.

Our results indicated that *H. pylori* infection was strongly associated with the development of stomach cancer. Based on the available evidence, *H. pylori* infection induces stomach cancer through direct and indirect pathways. The direct action of *H. pylori* on gastric epithelial cells is thought to be mediated by the induction of protein modulation and genetic mutations. Its indirect action on gastric epithelial cells is thought to be through inflammation. Both pathways work together to promote gastric carcinogenesis [21]. In addition, CagA apparently interacts with some host proteins that regulate cell growth, cell motility, and cell polarity. These interactions with CagA induce morphological transformations that may predispose cells to epigenetic changes involved in gastric carcinogenesis [22].

Our results revealed a positive relationship between cigarette smoking and the development of stomach cancer. Cigarette smoke contains over 7,000 toxic chemicals, including human carcinogens [23]. These toxins and carcinogens can cause direct DNA damage. Since DNA controls cells’ normal growth and function, DNA damage can alter cells’ growth patterns, and abnormal gastric epithelial cells with DNA damage can turn into cancer [24,25].

This systematic review showed that drinking alcohol increased the risk of developing stomach cancer. Acetaldehyde, the first and most toxic metabolite of ethanol, is a human carcinogen that can induce DNA lesions by inhibiting DNA methylation and by interacting with retinoid metabolism [26]. DNA lesions may lead to cell mutations, which convert a normal cell into cancer [27]. In addition, alcohol can act as an irritant and cause mucosal damage. The damaged cells may try to repair themselves, which could lead to DNA changes that can be a step toward cancer [28].

According to our results, the risk of stomach cancer of former drinkers was higher than that of current drinkers. One possible explanation for this finding is that former drinkers might be heavy drinkers who had drunk alcohol for many years, but were forced to quit drinking alcohol because of severe liver and gastric complications.

Pickled vegetables may increase the risk of stomach cancer because they contain large amounts of salt and because key nutrients are lost in vegetables under acidic and oxygenic conditions [29,30]. Furthermore, pickled vegetables are considered to be a possible source of nitrosamines, which may contribute to gastric carcinogenesis. The contamination of pickled vegetables with fungi has also been postulated to contribute to the incidence of stomach cancer [31].

Based on our findings, fruit and vegetable consumption was associated with a substantial reduction in stomach cancer risk. It has been postulated that the anti-carcinogenic effects of fruits and vegetables may be attributed to the antioxidant effect of their vitamin content, especially vitamin C and beta-carotene. Antioxidants neutralize reactive oxygen free radicals, which cause DNA damage [32,33]. Damaged DNA may lead to genetic modifications and carcinogenesis [24,25].

Our results showed a protective, but non-significant accusation between stomach cancer and overweight and obesity. However, the between-study heterogeneity was high (I^2^=86%). When we performed a sensitivity analysis, the overall effect changed from protective to a significant risk elevation (OR, 1.14; 95% CI, 1.03 to 1.26). Chen et al. [34]. conducted a meta-analysis including studies published before 2013 that were indexed in MEDLINE and EMBASE to address the association between gastric cancer and BMI. They reported that the relative risk of gastric cancer was 1.01 (95% CI, 0.96 to 1.07) for overweight and 1.06 (95% CI, 0.99 to 1.12) for obesity, and neither of those associations was statistically significant. Based on the current evidence, BMI does not seem to have a significant effect on the incidence of stomach cancer.

This systematic review has a few limitations and potential biases. There were some studies, mostly old, that seemed potentially eligible to be included in this meta-analysis, but neither their full texts nor their corresponding authors were accessible. This issue might have introduced selection bias in our results. Furthermore, several epidemiological studies that investigated the associations between stomach cancer and some nutritional and behavioral risk factors were excluded from the meta-analysis because they were not consistent with the inclusion criteria of this review. This issue may also raise the possibility of selection bias.

## CONCLUSION

This meta-analysis provided a clear picture of several behavioral and nutritional factors that play pivotal roles in the development of stomach cancer. These results are helpful and may be utilized for ranking and prioritizing preventable risk factors to implement effective interventions and community-based prevention programs. We reemphasize that both the strength of associations and the prevalence of factors in the community should be taken into account when ranking and prioritizing stomach cancer–associated factors.

## Figures and Tables

**Table 1.** Results of the sensitivity analysis

Variables	Sensitivity analysis							
	Before				After			
	Study (n)	χ^2^	I^2^ (%)	OR (95% CI)	Study (n)	χ^2^	I^2^ (%)	OR (95% CI)
*Helicobacter pylori*	68	0.001	86	2.56 (2.18, 3.00)	49	0.003	47	2.13 (1.89, 2.41)
Smoking								
Current	95	0.001	78	1.61 (1.49, 1.75)	73	0.001	49	1.66 (1.54, 1.79)
Former	52	0.001	65	1.43 (1.29, 1.59)	50	0.001	44	1.35 (1.24, 1.47)
Alcohol								
Current	72	0.001	83	1.19 (1.10, 1.29)	60	0.001	50	1.05 (0.99, 1.11)
Former	11	0.001	84	1.73 (1.17, 2.56)	9	0.050	48	2.01 (1.48, 2.72)
Body mass index	14	0.001	86	0.89 (0.74, 1.08)	10	0.090	41	1.14 (1.03, 1.26)
Physical activity	7	0.090	45	0.83 (0.68, 1.02)	NA	-	-	-
Fruit consumption	14	0.001	86	0.48 (0.37, 0.63)	11	0.070	42	0.64 (0.55, 0.75)
Vegetable consumption	9	0.001	74	0.62 (0.49. 0.79)	6	0.140	40	0.70 (0.58, 0.84)
Pickled vegetable consumption	16	0.060	39	1.28 (1.09, 1.51)	NA	-	-	-
Black tea intake	13	0.002	62	1.00 (0.84, 1.20)	12	0.120	34	0.94 (0.83,1.07)
Green tea intake	16	0.220	22	0.88 (0.80, 0.97)	NA	-	-	-
Coffee intake	12	0.160	29	0.99 (0.88, 1.11)	NA	-	-	-
Fish consumption	11	0.001	76	0.79 (0.61, 1.03)	9	0.070	45	0.68 (0.55, 0.83)
Red meat consumption	7	0.001	83	1.31 (0.87, 1.96)	4	0.340	11	0.91 (0.77, 1.09)

OR, odds ratio; CI, confidence interval; NA, not available.
