# Supplementary material for: Risk factors for stomach cancer: a systematic review and meta-analysis
Source: Epidemiol Health. 2020 Feb 2;42:e2020004. doi: 10.4178/epih.e2020004 (PMC7056944; doi:10.4178/epih.e2020004)
Supplement: Supplementary file 1 [file epih-42-e2020004-suppl.pdf]

Supplementary file 1: Characteristics of the included studies (sorted by authors' names)

Author	Country	Sex	Age (yr)	Study	Effect size	Adjustment	Sample	NOS score	Quality
Abnet 2011[1]	China	Female	40-70	Case-control	Odds Ratio	Adjusted	423	********	High
Agudo 1992[2]	Spain	Male	31-88	Case-control	Odds Ratio	Adjusted	708	*******	High
Agudo 2012[3]	European	Both	30-70	Cohort	Hazard Ratio	Adjusted	441,211	********	High
Al-Qadasl 2016[4]	Yamane	Both	57.80	Case-control	Odds Ratio	Unadjusted	210	********	High
Amadori 1995[5]	Italy	Both	No data	Case-control	Odds Ratio	Adjusted	662	********	High
Andereotti 2010[6]	USA	Both	40-70	Cohort	Hazard Ratio	Adjusted	67,947	********	High
Asombang 2013[7]	Zambia	Both	56.50	Case-control	Odds Ratio	Adjusted	140	*******	High
Bao 2004[8]	China	Both	30-74	Case-control	Odds Ratio	Adjusted	1,890	********	High
Baroudi 2014[9]	Tunisia	Both	20-89	Case-control	Odds Ratio	Adjusted	253	*******	High
Barstad 2005[10]	Denmark	Both	No data	Cohort	Risk Ratio	Adjusted	28,463	********	High
Behnampour 2014[11]	Iran	Both	62.85	Case-control	Odds Ratio	Adjusted	468	********	High
Blakely 2013[12]	New Zealand	Both	≥25	Cohort	Rate Ratio	Adjusted	94,524	********	High
Blaster 1995[13]	Japan	Male	58.75	Case-control	Odds Ratio	Adjusted	206	********	High
Boffetta 2005[14]	Norway	Male	No data	Cohort	Risk Ratio	Adjusted	10,136	********	High
Brenner 2002[15]	Germany	Both	65.67	Case-control	Odds Ratio	Adjusted	434	*******	High
Brenner 2004[16]	Germany	Both	≤80	Case-control	Odds Ratio	Adjusted	428	********	High
Cai 2000[17]	China	Both	32-78	Case-control	Odds Ratio	Adjusted	202	*********	High
Cai 2003[18]	China	Both	30-79	Case-control	Odds Ratio	Adjusted	603	*******	High
Cai 2016[19]	China-Jap-Korea	Both	57.60	Case-control	Odds Ratio	Adjusted	3,566	*******	High
Campbell 2007[20]	USA	Both	20-74	Case-control	Odds Ratio	Adjusted	1,932	********	High
Campos 2006[21]	Colombia	Both	49-75	Case-control	Odds Ratio	Adjusted	647	*******	High
Chang 2001[22]	Korea	Both	20-79	Case-control	Odds Ratio	Adjusted	272	*******	High
Chen 2000[23]	Taiwan	Both	31-86	Case-control	Odds Ratio	Adjusted	649	*******	High
Chen 2002[24]	USA	Both	60.34	Case-control	Odds Ratio	Adjusted	573	********	High
Chen 2009[25]	Taiwan	Male	No Data	Case-control	Odds Ratio	Adjusted	246	*******	High
Chen 2016[26]	German	Both	50-75	Cohort	Hazard Ratio	Adjusted	9,506	********	High
Cho 2010[27]	Korea	Both	57.25	Case-control	Odds Ratio	Adjusted	3,381	*******	High
Choi 1991[28]	Japan	Male	49.65	Case-control	Odds Ratio	Adjusted	1,536	*******	High
Choi 2017[29]	Korea	Both	≥20	Cohort	Hazard Ratio	Adjusted	23,323,730	********	High
Chow 1998[30]	USA	Both	30-79	Case-control	Odds Ratio	Adjusted	291	********	High
Chow 1999[31]	Poland	Both	21-79	Case-control	Odds Ratio	Adjusted	944	********	High
Chung 2010[32]	Korea	Both	18-45	Case-control	Odds Ratio	Adjusted	6,242	********	High
Coggon 1989[33]	England	Both	No data	Case-control	Odds Ratio	Adjusted	285	********	High
Corley 2008[34]	USA	Both	47.51	Case-control	Odds Ratio	Adjusted	2,905	********	High
Correa 1985[35]	USA	Both	No data	Case-control	Odds Ratio	Adjusted	782	********	High
D'Avanzo[36]	Italy	Both	19-74	Case-control	Odds Ratio	Adjusted	2,799	*****	Low
de Larrea-Baz 2017[37]	Spain	Both	20-85	Case-control	Odds Ratio	Adjusted	2,335	********	High
de Menezes 2015[38]	Brazil	Both	18-75	Case-control	Odds Ratio	Adjusted	34,017	*******	High
de Souza Moura 2014[39]	Brazil	Both	18-65	Case-control	Odds Ratio	Adjusted	231,102	*******	High
Demirer 1990[40]	Turkey	Both	28-78	Case-control	Odds Ratio	Adjusted	200	********	High
Duan 2009[41]	USA	Both	60.01	Case-control	Odds Ratio	Adjusted	2,294	********	High
Duell 2011[42]	Europe	Both	35-70	Cohort	Hazard Ratio	Adjusted	110,187	********	High
Ekstrom 2000[43]	Sweden	Both	40-79	Case-control	Odds Ratio	Adjusted	1,732	********	High
Ellison-Loschman 2017[44]	New Zealand	Both	35-65	Case-control	Odds Ratio	Adjusted	645	********	High
Engel 2003[45]	USA	Both	30-79	Case-control	Odds Ratio	Adjusted	1,324	********	High
Enorth 2000[46]	Sweden	Both	71.23	Case-control	Odds Ratio	Adjusted	396	*******	High
Eom 2015[47]	Korea	Both	30-80	Cohort	Hazard Ratio	Adjusted	2,176,501	********	High
Epplein 2010[48]	China	Both	40-74	Cohort	Odds Ratio	Adjusted	136,442	*****	Low
Epplein 2014[49]	China	Male	40-74	Case-control	Odds Ratio	Adjusted	677	********	High
Fang 2015[50]	China	Both	60.23	Case-control	Odds Ratio	Adjusted	604	********	High
Fei 2006[51]	China	Both	28-93	Case-control	Odds Ratio	Adjusted	756	********	High
Forman 1991[52]	UK	Male	41-64	Case-control	Odds Ratio	Adjusted	145	********	High
Franandez 1999[53]	Italy	Both	<75	Case-control	Odds Ratio	Unadjusted	8,735	****	Low
Freedman 2007[54]	USA	Both	62.50	Cohort	Hazard Ratio	Adjusted	474,606	*******	High
Fukuda 1995[55]	Japan	Both	54.39	Case-control	Odds Ratio	Adjusted	1,049	*******	High
Gajalkshmi 1996[56]	India	Both	25-75	Case-control	Odds Ratio	Adjusted	776	********	High
Galanis 1998[57]	USA	Both	No data	Cohort	Hazard Ratio	Adjusted	11,907	********	High
Gallus 2009[58]	Italy	Both	19-80	Case-control	Odds Ratio	Adjusted	3,627	*******	High
Gao 1999[59]	China	Both	59.62	Case-control	Odds Ratio	Adjusted	387	********	High
Gao 2011[60]	China	Both	50-66	Case-control	Odds Ratio	Adjusted	2,429	*******	High
Goh 2007[61]	Malaysia	Both	59.73	Case-control	Odds Ratio	Adjusted	261	*******	High
Goldbohm 1996[62]	Netherland	Both	55-69	Cohort	Rate Ratio	Adjusted	120,852	*******	High
Gomez Zuleta 2011[63]	Columbia	Both	18-89	Case-control	Odds Ratio	Adjusted	183	*****	Low
Gunathilake 2018[64]	Korea	Both	53.73	Case-control	Odds Ratio	Adjusted	1,245	*******	High
Gwack 2006[65]	Korea	Both	63.00	Case-control	Odds Ratio	Unadjusted	500	*******	High
Hamada 2002[66]	Brazil	Both	38-89	Case-control	Odds Ratio	Adjusted	288	*******	High
Hansen 1999[67]	Norwegian	Both	No data	Case-control	Odds Ratio	Adjusted	1,191	********	High
Hansson 1993a[68]	Sweden	Both	40-79	Case-control	Odds Ratio	Adjusted	1,007	********	High
Hansson 1993b[69]	Sweden	Both	60-70	Case-control	Odds Ratio	Adjusted	215	*******	High
Hansson 1994[70]	Sweden	Both	40-79	Case-control	Odds Ratio	Adjusted	1,017	*******	High
Haruma 2000[71]	Japan	Both	26.66	Case-control	Odds Ratio	Adjusted	150	*******	High
Held 2004[72]	Sweden	Both	50-70	Case-control	Odds Ratio	Adjusted	196	********	High
Hirohata 1997[73]	Japan	Both	No data	Case-control	Odds Ratio	Adjusted	780	********	High
Hisihida 2010[74]	Japan	Both	25-84	Case-control	Odds Ratio	Adjusted	2,175	*******	High
Hoang 2016[75]	Korea	Both	53.73	Case-control	Odds Ratio	Adjusted	1,245	******	Low
Hoey 1981[76]	France	Both	62.94	Case-control	Odds Ratio	Unadjusted	208	*****	Low
Hoshiyama 1992a[77]	Japan	Both	45-75	Case-control	Odds Ratio	Adjusted	588	*******	High
Hoshiyama 1992b[78]	Japan	Both	54-65	Case-control	Odds Ratio	Adjusted	734	********	High
Hoshiyama 2004[79]	Japan	Both	40-79	Case-control	Odds Ratio	Adjusted	442	********	High
Huang 1999[80]	Japan	Both	20-79	Case-control	Odds Ratio	Adjusted	29,469	********	High
Huang 2000[81]	Japan	Both	40-79	Case-control	Odds Ratio	Adjusted	19,346	********	High

Huerta 2017[82]	Spain	Both	20-85	Case-control	Odds Ratio	Adjusted	3,653	********	High
Iclic 2011[83]	Turkey	Both	56.25	Case-control	Odds Ratio	Adjusted	506	********	High
Inoue 1994[84]	Japan	Male	56.60	Case-control	Odds Ratio	Adjusted	1,336	*******	High
Inoue 1998[85]	Japan	Both	40-60	Case-control	Odds Ratio	Adjusted	22,021	*******	High
Inoue 2002[86]	Japan	Female	39-82	Case-control	Odds Ratio	Adjusted	2,190	********	High
Inoue 2008[87]	Japan	Both	45-74	Cohort	Hazard Ratio	Adjusted	79,771	********	High
Ito 2003[88]	Japan	Female	48.72	Case-control	Odds Ratio	Adjusted	36,998	********	High
Jang 2018[89]	Korea	Both	53.80	Cohort	Hazard Ratio	Adjusted	2,458	********	High
Jayalekshmi 2015[90]	Indian	Male	30-84	Cohort	Risk Ratio	Adjusted	65,553	*******	High
Jedrychowski 1986[91]	Poland	Both	49-70	Case-control	Odds Ratio	Adjusted	330	********	High
Jedrychowski 1993[92]	Poland	Male	<75	Case-control	Odds Ratio	Adjusted	1,040	*******	High
Ji 1996[93]	China	Both	20-69	Case-control	Odds Ratio	Adjusted	2,575	********	High
Kabat 1992[94]	USA	Both	No data	Case-control	Odds Ratio	Adjusted	6,925	*******	High
Kamanger 2006[95]	Finland	Both	50-69	Case-control	Odds Ratio	Adjusted	468	*******	High
Karagulle 2014[96]	Turkey	Both	58.44	Case-control	Odds Ratio	Unadjusted	450	*****	Low
Kato 1990[97]	Japan	Both	29-70	Case-control	Odds Ratio	Unadjusted	3,441	*****	Low
Kato 2004[98]	Japan	Both	21-71	Case-control	Odds Ratio	Unadjusted	9,081	*******	High
Keck 2014[99]	China	Both	42.24	Case-control	Odds Ratio	Adjusted	468	********	High
Keszei 2012[100]	Netherland	Both	55-69	Cohort	Odds Ratio	Unadjusted	120,852	***	Low
Kikuchi 2002[101]	Japan	Both	20-69	Case-control	Odds Ratio	Adjusted	1,601	********	High
Kim 1997[102]	Korea	Both	28-89	Case-control	Odds Ratio	Adjusted	320	******	Low
Kim 2002[103]	Korea	Both	55-74	Case-control	Odds Ratio	Adjusted	272	*******	High
Kim 2005[104]	Korea	Both	49.25	Case-control	Odds Ratio	Adjusted	590	********	High
Kim 2010[105]	Korea	Both	30-80	Cohort	Hazard Ratio	Adjusted	2,248,129	********	High
Kim 2015[106]	Korea	Male	58.20	Case-control	Odds Ratio	Adjusted	2,286	********	High
Ko 2013[107]	Korea	Both	30-90	Cohort	Odds Ratio	Unadjusted	9,724	******	Low
Komoto 1998[108]	Japan	Both	63.92	Case-control	Odds Ratio	Adjusted	246	*******	High
Kono 1988[109]	Japan	Both	25-75	Case-control	Odds Ratio	Adjusted	417	********	High
Kuriyama 2005[110]	Japan	Both	56.65	Cohort	Risk Ratio	Adjusted	31,345	********	High
La Vecchia 1987[111]	Italy	Both	28-74	Case-control	Odds Ratio	Adjusted	680	*******	High
La Vecchia 1988[112]	Italy	Both	45-74	Case-control	Odds Ratio	Unadjusted	6,711	*****	Low
La Vecchia 1997[113]	Italy	Both	19-74	Case-control	Odds Ratio	Unadjusted	2,799	*****	Low
Lagergren 2000[114]	Sweden	Both	No data	Case-control	Odds Ratio	Adjusted	1,082	********	High
Lai 2016[115]	Vietnam	Male	30-84	Case-control	Odds Ratio	Adjusted	1,082	*******	High
Larsson 2006[116]	Sweden	Female	53.72	Cohort	Hazard Ratio	Adjusted	61,433	*******	High
Lazarevic 2011[117]	Serbia	Both	45-85	Case-control	Odds Ratio	Adjusted	306	*******	High
Lee 1995[118]	Korea	Both	25-65	Case-control	Odds Ratio	Adjusted	426	*******	High
Lee 1998[119]	Korea	Both	1284	Case-control	Odds Ratio	Adjusted	288	********	High
Lee 2002[120]	Korea	Both	40-56	Case-control	Odds Ratio	Adjusted	268	*******	High
Lee 2003[121]	Korea	Both	>40	Case-control	Odds Ratio	Adjusted	268	*******	High
Levi 2013[122]	Israel	Male	16-19	Cohort	Hazard Ratio	Adjusted	1,088,530	********	High
Li 2010[123]	Japan	Both	40-79	Cohort	Risk Ratio	Adjusted	42,470	********	High
Limburg 2001[124]	China	Both	50-60	Case-control	Odds Ratio	Adjusted	373	********	High
Lin 2014[125]	China	Both	40-75	Case-control	Odds Ratio	Adjusted	316	********	High
Lindblad 2005[126]	UK	Both	40-84	Case-control	Odds Ratio	Adjusted	10,522	********	High
Lissowska 2004[127]	Poland	Both	50-70	Case-control	Odds Ratio	Adjusted	737	********	High
Lopez- Carrillo 2004[128]	Mexico	Both	28-86	Case-control	Odds Ratio	Adjusted	665	*******	High
Ma 2015[129]	Korea	Both	No data	Cohort	Hazard Ratio	Adjusted	18,863	*******	High
Machida-Montani 2004[130]	Japan	Both	57.53	Case-control	Odds Ratio	Adjusted	357	*******	High
Machova 2007[131]	Czech	Both	30-64	Case-control	Odds Ratio	Adjusted	38,150	*******	High
Maeda 2000[132]	Australia	Both	32-73	Case-control	Odds Ratio	Adjusted	160	*******	High
Mao 2002[133]	Canada	Both	30-70	Case-control	Odds Ratio	Adjusted	3,378	********	High
Mao 2011[134]	China	Both	51.90	Case-control	Odds Ratio	Adjusted	400	*******	High
Mathew 2000[135]	Indian	Both	20-75	Case-control	Odds Ratio	Adjusted	499	*******	High
Meine 2011[136]	Brazil	Both	64.66	Case-control	Odds Ratio	Adjusted	87	*******	High
Merry 2007[137]	Nether lands	Both	55-69	Cohort	Risk Ratio	Adjusted	120,852	********	High
Minami 2003[138]	Japan	Both	61.78	Case-control	Odds Ratio	Adjusted	3,058	********	High
Moller 1994[139]	Denmark	Both	No data	Cohort	Risk Ratio	Unadjusted	43,965	*******	High
Moy 2010[140]	China	Male	45-64	Cohort	Hazard Ratio	Adjusted	18,244	********	High
Mu 2005[141]	China	Both	≥40	Case-control	Odds Ratio	Adjusted	621	********	High
Munoz 2001[142]	Venezuela	Both	≥35	Case-control	Odds Ratio	Adjusted	777	*******	High
Nagano 2001[143]	Japan	Both	55.25	Cohort	Risk Ratio	Adjusted	38,540	********	High
Nakaya 2005[144]	Japan	Male	40-64	Cohort	Risk Ratio	Adjusted	25,279	********	High
Navrro Silvera 2008[145]	USA	Both	30-79	Case-control	Odds Ratio	Adjusted	1,294	*******	High
Nishimoto 2002[146]	Brazil	Both	40-79	Case-control	Odds Ratio	Adjusted	472	*******	High
Nomura 1990[147]	USA	Male	45-75	Cohort	Risk Ratio	Adjusted	7,990	*******	High
Nomura 1991[148]	USA	Both	49-70	Case-control	Odds Ratio	Adjusted	218	*******	High
Nomura 2002[149]	USA	Both	50-90	Case-control	Odds Ratio	Adjusted	522	********	High
Nomura 2005[150]	USA	Both	26-97	Case-control	Odds Ratio	Adjusted	635	********	High
Nomura 2012[151]	USA	Both	45-75	Cohort	Hazard Ratio	Adjusted	182,441	*******	High
Ogimoto 1995[152]	Japan	Female	<40	Case-control	Odds Ratio	Adjusted	260	*******	High
Pakseresht 2011[153]	Iran	Both	64.55	Case-control	Odds Ratio	Adjusted	590	********	High
Palli 1992[154]	Italy	Both	45-75	Case-control	Odds Ratio	Adjusted	2,082	********	High
Palli 2001a[155]	Italy	Both	50-64	Case-control	Odds Ratio	Unadjusted	943	*****	Low
Palli 2001b[156]	Italy	Both	50-64	Case-control	Odds Ratio	Unadjusted	943	*******	High
Parent 2010[157]	Canada	Male	58.98	Case-control	Odds Ratio	Adjusted	784	********	High
Parsonnet 1991[158]	USA	Both	53.70	Case-control	Odds Ratio	Adjusted	372	********	High
Parsonnet 1997[159]	USA	Male	55.48	Case-control	Odds Ratio	Adjusted	242	********	High
Peleteiro 2010[160]	Portugal	Both	18-92	Case-control	Odds Ratio	Adjusted	458	********	High
Persson 2008[161]	Japan	Both	40-59	Case-control	Odds Ratio	Adjusted	1,022	********	High
Persson 2011[162]	Sweden	Both	16-68	Case-control	Odds Ratio	Adjusted	176	********	High
Phukan 2006[163]	India	Both	57.00	Case-control	Odds Ratio	Adjusted	987	********	High
Pourfarzi 2009[164]	Iran	Both	64.70	Case-control	Odds Ratio	Adjusted	611	*******	High
Queiroz 1999[165]	Brazil	Both	32-96	Case-control	Odds Ratio	Adjusted	254	*******	High

Ramon 1993[166]	Spain	Both	30-80	Case-control	Odds Ratio	Adjusted	351	********	High
Ramos 2018[167]	Brazil	Both	20-80	Case-control	Odds Ratio	Adjusted	739	*******	High
Rao 2002[168]	India	Both	51.90	Case-control	Odds Ratio	Adjusted	2,354	*******	High
Rapp 2005[169]	Austria	Both	35-54	Cohort	Hazard Ratio	Adjusted	145,931	********	High
Rudi 1995[170]	Germany	Both	26-83	Case-control	Odds Ratio	Adjusted	222	*******	High
Rudi 1997[171]	Germany	Both	30-87	Case-control	Odds Ratio	Adjusted	180	*******	High
Rugge 1999[172]	Italy	Both	16-40	Case-control	Odds Ratio	Adjusted	210	*******	High
Saha 1991[173]	Japan	Both	31-90	Case-control	Odds Ratio	Unadjusted	351	*****	Low
Samanic 2006[174]	Sweden	Male	34.30	Cohort	Risk Ratio	Adjusted	362,552	********	High
Sarker 2017[175]	Bangladesh	Both	46.92	Case-control	Odds Ratio	Adjusted	634	*******	High
Sasazuki 2002[176]	Japan	Male	40-59	Cohort	Rate Ratio	Adjusted	19,657	*******	High
Sasazuki 2004[177]	Japan	Both	40-69	Cohort	Risk Ratio	Adjusted	72,943	********	High
Sasazuki 2006[178]	Japan	Both	57.40	Case-control	Odds Ratio	Adjusted	1,022	********	High
Sekikawa 1998[179]	Japan	Male	>40	Case-control	Odds Ratio	Adjusted	45	*******	High
Setiawan 2001[180]	China	Both	48.63	Case-control	Odds Ratio	Adjusted	566	********	High
Siman 1997[181]	Sweden	Both	34-70	Case-control	Odds Ratio	Adjusted	280	********	High
Siman 2007[182]	Sweden	Both	50.83	Case-control	Odds Ratio	Adjusted	605	*******	High
Sjodahl 2008a[183]	Norway	Both	50.01	Cohort	Odds Ratio	Unadjusted	73,133	******	Low
Sojdahl 2008b[184]	Norway	Both	49.60	Cohort	Hazard Ratio	Adjusted	73,133	*******	High
Somi 2015[185]	Iran	Both	21-84	Case-control	Odds Ratio	Unadjusted	616	*****	Low
Sriamporn 2003[186]	Thailand	Both	No data	Case-control	Odds Ratio	Adjusted	393	*******	High
Steevens 2010[187]	Netherland	Both	55-70	Cohort	Rate Ratio	Adjusted	3,962	********	High
Stemmermann 1990[188]	Japan	Male	45-65	Cohort	Risk Ratio	Adjusted	4,762	*******	High
Strumylaite 2006[189]	Lithuania	Both	22-86	Case-control	Odds Ratio	Adjusted	1,516	*******	High
Sun 2013[190]	China	Both	40-69	Case-control	Odds Ratio	Adjusted	940	*******	High
Sunwanrungruang 2008[191]	Thailand	Male	39-70	Case-control	Odds Ratio	Adjusted	303	*******	High
Takezaki 2001[192]	China	Both	40-79	Case-control	Odds Ratio	Adjusted	520	********	High
Tally 1991[193]	England	Both	54-71	Case-control	Odds Ratio	Adjusted	321	*******	High
Terry 1998[194]	Sweden	Both	50-70	Cohort	Risk Ratio	Unadjusted	11,546	******	Low
Terry 2000[195]	Sweden	Both	67.65	Case-control	Odds Ratio	Adjusted	1,073	********	High
Tønnessen 1994[196]	Denmark	Both	44-55	Cohort	Risk Ratio	Unadjusted	18,368	******	Low
Tran 2005[197]	China	Both	40-69	Cohort	Risk Ratio	Adjusted	29,584	*******	High
Tsubone 2001[198]	Japan	Both	56.72	Cohort	Risk Ratio	Adjusted	26,311	********	High
Tsugane 2004[199]	Japan	Female	40-59	Cohort	Risk Ratio	Adjusted	39,065	********	High
Turns 1982[200]	France	Both	25-75	Case-control	Odds Ratio	Adjusted	2,139	*********	High
Wang 2011[201]	China	Both	30-79	Case-control	Odds Ratio	Adjusted	771	********	High
Wang 2012[202]	China	Both	30-79	Case-control	Odds Ratio	Adjusted	771	*******	High
Wang 2015[203]	China	Both	No data	Case-control	Odds Ratio	Adjusted	480	*******	High
Wang 2017[204]	Asia	Both	59.50	Case-control	Odds Ratio	Adjusted	1,970	********	High
Ward 1999[205]	Mexico	Both	≥20	Case-control	Odds Ratio	Adjusted	972	*******	High
Watabe 1998[206]	Japan	Both	40-79	Case-control	Odds Ratio	Adjusted	726	********	High
Watanabe 1997[207]	Japan	Both	35-89	Case-control	Odds Ratio	Adjusted	270	********	High
Webb 1996[208]	China	Male	45-64	Case-control	Odds Ratio	Adjusted	348	*******	High
Wen 2010[209]	China	Both	40-75	Case-control	Odds Ratio	Adjusted	900	*******	High
Wolk 2001[210]	Sweden	Both	34.8	Cohort	Risk Ratio	Unadjusted	28,129	*******	High
Wu 1998[211]	Japan	Both	40-70	Case-control	Odds Ratio	Adjusted	270	*******	High
Wu 2001[212]	USA	Both	30-74	Case-control	Odds Ratio	Adjusted	2,076	********	High
Wu 2003[213]	USA	Both	30-74	Case-control	Odds Ratio	Adjusted	2,076	********	High
Wu 2009[214]	Taiwan	Both	47.83	Case-control	Odds Ratio	Adjusted	591	*******	High
Wu -william 1990[215]	USA	Male	46.95	Case-control	Odds Ratio	Adjusted	274	********	High
Xibin 2002[216]	China	Both	30-75	Case-control	Odds Ratio	Adjusted	840	********	High
Yamaoka 1998[217]	Japan	Both	42-84	Case-control	Odds Ratio	Adjusted	220	*******	High
Yang 2011[218]	China	Both	40-75	Case-control	Odds Ratio	Adjusted	900	******	Low
Yang 2017[219]	East Asians	Both	53.04	Case-control	Odds Ratio	Adjusted	1,500	********	High
Ye 1999[220]	Sweden	Both	40-79	Case-control	Odds Ratio	Adjusted	1,678	********	High
You 1998[221]	China	Both	35-64	Case-control	Odds Ratio	Adjusted	1,695	********	High
Yu 1991[222]	China	Both	50-59	Case-control	Odds Ratio	Adjusted	2,760	********	High
Yuan 1999[223]	China	Male	45-64	Case-control	Odds Ratio	Adjusted	340	*******	High
Yun 2005[224]	Korea	Male	43.18	Cohort	Risk Ratio	Adjusted	733,134	*******	High
Zamani 2013[225]	Iran	Both	62.28	Case-control	Odds Ratio	Unadjusted	837	*****	Low
Zaridze 1999[226]	Russia	Male	55-70	Case-control	Odds Ratio	Adjusted	1,058	*******	High
Zendendel 2008[227]	Scandinavian	Male	20-60	Cohort	Risk Ratio	Adjusted	361,280	********	High
Zhang 1996[228]	USA	Both	No data	Case-control	Odds Ratio	Adjusted	199	*******	High
Zhang 2009[229]	Korea	Both	58.50	Case-control	Odds Ratio	Adjusted	942	*******	High
Zhang 2011[230]	China	Both	35-77	Case-control	Odds Ratio	Adjusted	645	*******	High
Zhong 2012[231]	China	Both	35-77	Case-control	Odds Ratio	Adjusted	617	********	High

References

1. Abnet C, Zheng W, Ye W, Kamangar F, Ji B, Persson C, et al. Plasma pepsinogens, antibodies against Helicobacter pylori, and risk of gastric cancer in the Shanghai Women's Health Study Cohort. Br J Cancer. 2011; 104:1511-6.

2. Agudo A, González CA, Marcos G, Sanz M, Saigi E, Verge J, et al. Consumption of alcohol, coffee, and tobacco, and gastric cancer in Spain. Cancer Causes Control. 1992; 3:137-43.

3. Agudo A, Bonet C, Travier N, González CA, Vineis P, Bueno-de-Mesquita HB, et al. Impact of cigarette smoking on cancer risk in the European prospective investigation into cancer and nutrition study. J Clin Oncol. 2012; 30:4550-7.

4. Al-Qadasi F, Shah S, Ghazi H. Tobacco chewing and risk of gastric cancer: a case--control study in Yemen. East Mediterr Health J. 2016; 22.

5. Amadori D, Nanni O, Ricci M, Falcini F, Decarli A, Palli D, et al. Hospital versus population controls in a retrospective study on diet and stomach cancer. The European Journal of Public Health. 1995; 5:209-14.

6. Andreotti G, Hou L, Freeman LEB, Mahajan R, Koutros S, Coble J, et al. Body mass index, agricultural pesticide use, and cancer incidence in the Agricultural Health Study cohort. Cancer Causes Control. 2010; 21:1759-75.

7. Asombang AW, Kayamba V, Mwanza-Lisulo M, Colditz G, Mudenda V, Yarasheski K, et al. Gastric cancer in Zambian adults: a prospective case-control study that assessed dietary intake and antioxidant status by using urinary isoprostane excretion. Am J Clin Nutr. 2013; 97:1029-35.

8. Bao P, Gao L, Liu D, Tao M, Jin F. A case control study on risk factors for stomach cancer in urban Shanghai. Chinese Journal of Clinical Oncology. 2004; 1:131-8.

9. Baroudi O, Chaaben AB, Mezlini A, Moussa A, Omrane I, Jilson I, et al. Impact of lifestyle factors and nutrients intake on occurrence of gastrointestinal cancer in Tunisian population. Tumour biology : the journal of the International Society for Oncodevelopmental Biology and Medicine. 2014; 35:5815-22.

10. Barstad B, Sørensen T, Tjønneland A, Johansen D, Becker U, Andersen I, et al. Intake of wine, beer and spirits and risk of gastric cancer. European journal of cancer prevention : the official journal of the European Cancer Prevention Organisation (ECP). 2005; 14:239-43.

11. Behnampour N, Hajizadeh E, Zayeri F, Semnani S. Modeling of influential predictors of gastric cancer incidence rates in Golestan Province, North Iran. Asian Pac J Cancer Prev. 2014; 15:1111-7.

12. Blakely T, Barendregt JJ, Foster RH, Hill S, Atkinson J, Sarfati D, et al. The association of active smoking with multiple cancers: national census-cancer registry cohorts with quantitative bias analysis. Cancer Causes Control. 2013; 24:1243-55.

13. Blaser MJ, Perez-Perez GI, Kleanthous H, Cover TL, Peek RM, Chyou P, et al. Infection with Helicobacter pylori strains possessing cagA is associated with an increased risk of developing adenocarcinoma of the stomach. Cancer Res. 1995; 55:2111-5.

14. Boffetta P, Aagnes B, Weiderpass E, Andersen A. Smokeless tobacco use and risk of cancer of the pancreas and other organs. International journal of cancer. 2005; 114:992-5.

15. Brenner H, Arndt V, Bode G, Stegmaier C, Ziegler H, Stümer T. Risk of gastric cancer among smokers infected with Helicobacter pylori. International journal of cancer. 2002; 98:446-9.

16. Brenner H, Arndt V, Stegmaier C, Ziegler H, Rothenbacher D. Is Helicobacter pylori infection a necessary condition for noncardia gastric cancer? Am J Epidemiol. 2004; 159:252-8.

17. Cai L, Yu S-Z, Zhang Z-F. Helicobacter pylori infection and risk of gastric cancer in Changle County, Fujian Province, China. World J Gastroenterol. 2000; 6:374.

18. Cai L, Zheng Z-L, Zhang Z-F. Risk factors for the gastric cardia cancer: a case-control study in Fujian Province. World J Gastroenterol. 2003; 9:214.

19. Cai H, Ye F, Michel A, Murphy G, Sasazuki S, Taylor PR, et al. Helicobacter pylori blood biomarker for gastric cancer risk in East Asia. Int J Epidemiol. 2016; 45:774-81.

20. Campbell PT, Sloan M, Kreiger N. Physical activity and stomach cancer risk: the influence of intensity and timing during the lifetime. Eur J Cancer. 2007; 43:593-600.

21. Campos F, Carrasquilla G, Koriyama C, Serra M, Carrascal E, Itoh T, et al. Risk factors of gastric cancer specific for tumor location and histology in Cali, Colombia. World J Gastroenterol. 2006; 12:5772.

22. Chang WK, Kim HY, Kim DJ, Lee J, Park CK, Yoo JY, et al. Association between Helicobacter pylori infection and the risk of gastric cancer in the Korean population: prospective case-controlled study. J Gastroenterol. 2001; 36:816-22.

23. Chen M-J, Chiou Y-Y, Wu D-C, Wu S-L. Lifestyle habits and gastric cancer in a hospital-based case-control study in Taiwan. Am J Gastroenterol. 2000; 95:3242.

24. Chen H, Ward MH, Graubard BI, Heineman EF, Markin RM, Potischman NA, et al. Dietary patterns and adenocarcinoma of the esophagus and distal stomach. Am J Clin Nutr. 2002; 75:137-44.

25. Chen M-J, Wu D-C, Lin J-M, Wu M-T, Sung F-C. Etiologic factors of gastric cardiac adenocarcinoma among men in Taiwan. World J Gastroenterol. 2009; 15:5472.

26. Chen X-Z, Schöttker B, Castro FA, Chen H, Zhang Y, Holleczek B, et al. Association of helicobacter pylori infection and chronic atrophic gastritis with risk of colonic, pancreatic and gastric cancer: A ten-year follow-up of the ESTHER cohort study. Oncotarget. 2016; 7:17182.

27. Cho S-J, Choi IJ, Kim CG, Lee JY, Kook M-C, Seong M-W, et al. Helicobacter pylori seropositivity is associated with gastric cancer regardless of tumor subtype in Korea. Gut and liver. 2010; 4:466.

28. Choi SY, Kahyo H. Effect of cigarette smoking and alcohol consumption in the etiology of cancers of the digestive tract. International journal of cancer. 1991; 49:381-6.

29. Choi YJ, Lee DH, Han K-D, Kim HS, Yoon H, Shin CM, et al. The relationship between drinking alcohol and esophageal, gastric or colorectal cancer: A nationwide population-based cohort study of South Korea. PloS one. 2017; 12:e0185778.

30. Chow W-H, Blaser MJ, Blot WJ, Gammon MD, Vaughan TL, Risch HA, et al. An inverse relation between cagA+ strains of Helicobacter pylori infection and risk of esophageal and gastric cardia adenocarcinoma. Cancer Res. 1998; 58:588-90.

31. Chow WH, Swanson CA, Lissowska J, Groves FD, Sobin LH, Nasierowska-Guttmejer A, et al. Risk of stomach cancer in relation to consumption of cigarettes, alcohol, tea and coffee in Warsaw, Poland. International journal of cancer. 1999; 81:871-6.

32. Chung HW, Noh SH, Lim J-B. Analysis of demographic characteristics in 3242 young age gastric cancer patients in Korea. World J Gastroenterol. 2010; 16:256.

33. Coggon D, Barker DJ, Cole RB, Nelson M. Stomach cancer and food storage. J Natl Cancer Inst. 1989; 81:1178-82.

34. Corley DA, Kubo A, Zhao W. Abdominal obesity and the risk of esophageal and gastric cardia carcinomas. Cancer Epidemiol Biomarkers Prev. 2008; 17:352-8.

35. Correa P, Fontham E, Pickle LW, Chen V, Lin Y, Haenszel W. Dietary determinants of gastric cancer in south Louisiana inhabitants. J Natl Cancer Inst. 1985; 75:645-54.

36. D'Avanzo B, La Vecchia C, Franceschi S. Alcohol consumption and the risk of gastric cancer. Nutr Cancer. 1994; 22:57-64.

37. de Larrea-Baz NF, Pérez-Gómez B, Michel A, Romero B, Lope V, Pawlita M, et al. Helicobacter pylori serological biomarkers of gastric cancer risk in the MCC-Spain case-control Study. Cancer epidemiology. 2017; 50:76-84.

38. de Menezes RF, Bergmann A, de Aguiar SS, Thuler LCS. Alcohol consumption and the risk of cancer in Brazil: a study involving 203,506 cancer patients. Alcohol. 2015; 49:747-51.

39. de Souza Moura MA, Bergmann A, de Aguiar SS, Thuler LCS. The magnitude of the association between smoking and the risk of developing cancer in Brazil: a multicenter study. BMJ open. 2014; 4:e003736.

40. Demirer T, Icli F, Uzunalimoglu O, Kucuk O. Diet and stomach cancer incidence a case–control study in Turkey. Cancer. 1990; 65:2344-8.

41. Duan L, Wu A, Sullivan-Halley J, Bernstein L. Passive smoking and risk of oesophageal and gastric adenocarcinomas. Br J Cancer. 2009; 100:1483.

42. Duell EJ, Travier N, Lujan-Barroso L, Clavel-Chapelon F, Boutron-Ruault M-C, Morois S, et al. Alcohol consumption and gastric cancer risk in the European Prospective Investigation into Cancer and Nutrition (EPIC) cohort–. Am J Clin Nutr. 2011; 94:1266-75.

43. Ekström AM, Serafini M, Nyrén O, Hansson LE, Ye W, Wolk A. Dietary antioxidant intake and the risk of cardia cancer and noncardia cancer of the intestinal and diffuse types: a population-based case-control study in Sweden. International journal of cancer. 2000; 87:133-40.

44. Ellison-Loschmann L, Sporle A, Corbin M, Cheng S, Harawira P, Gray M, et al. Risk of stomach cancer in Aotearoa/New Zealand: A Māori population based case-control study. PloS one. 2017; 12:e0181581.

45. Engel LS, Chow WH, Vaughan TL, Gammon MD, Risch HA, Stanford JL, et al. Population attributable risks of esophageal and gastric cancers. J Natl Cancer Inst. 2003; 95:1404-13.

46. Enroth H, Kraaz W, Engstrand L, Nyrén O, Rohan T. Helicobacter pylori strain types and risk of gastric cancer: a case-control study. Cancer Epidemiol Biomarkers Prev. 2000; 9:981-5.

47. Eom BW, Joo J, Kim S, Shin A, Yang H-R, Park J, et al. Prediction model for gastric cancer incidence in Korean population. PloS one. 2015; 10:e0132613.

48. Epplein M, Shu X-O, Xiang Y-B, Chow W-H, Yang G, Li H-L, et al. Fruit and vegetable consumption and risk of distal gastric cancer in the Shanghai Women's and Men's Health studies. Am J Epidemiol. 2010; 172:397-406.

49. Epplein M, Zheng W, Li H, Peek Jr RM, Correa P, Gao J, et al. Diet, Helicobacter pylori strain-specific infection, and gastric cancer risk among Chinese men. Nutr Cancer. 2014; 66:550-7.

50. Fang C, Huang Q, Lu L, Shi J, Sun Q, Xu GF, et al. Risk factors of early proximal gastric carcinoma in Chinese diagnosed using WHO criteria. J Dig Dis. 2015; 16:327-36.

51. Fei SJ, Xiao SD. Diet and gastric cancer: a case–control study in Shanghai urban districts. Chin J Dig Dis. 2006; 7:83-8.

52. Forman D, Newell D, Fullerton F, Yarnell J, Stacey A, Wald N, et al. Association between infection with Helicobacter pylori and risk of gastric cancer: evidence from a prospective investigation. BMJ. 1991; 302:1302-5.

53. Fernandez E, Chatenoud L, La Vecchia C, Negri E, Franceschi S. Fish consumption and cancer risk. Am J Clin Nutr. 1999; 70:85-90.

54. Freedman ND, Abnet CC, Leitzmann MF, Mouw T, Subar AF, Hollenbeck AR, et al. A prospective study of tobacco, alcohol, and the risk of esophageal and gastric cancer subtypes. Am J Epidemiol. 2007; 165:1424-33.

55. Fukuda H, Saito D, Hayashi S, Hisai H, Ono H, Yoshida S, et al. Helicobacter pylori infection, serum pepsinogen level and gastric cancer: a case-control study in Japan. Jpn J Cancer Res. 1995; 86:64-71.

56. Gajalakshmi CK, Shanta V. Lifestyle and risk of stomach cancer: a hospital-based case-control study. Int J Epidemiol. 1996; 25:1146-53.

57. Galanis DJ, Kolonel LN, Lee J, Nomura A. Intakes of selected foods and beverages and the incidence of gastric cancer among the Japanese residents of Hawaii: a prospective study. Int J Epidemiol. 1998; 27:173-80.

58. Gallus S, Tramacere I, Tavani A, Bosetti C, Bertuccio P, Negri E, et al. Coffee, black tea and risk of gastric cancer. Cancer Causes Control. 2009; 20:1303-8.

59. Gao CM, Takezaki T, Ding JH, Li MS, Tajima K. Protective effect of allium vegetables against both esophageal and stomach cancer: a simultaneous case-referent study of a high-epidemic area in Jiangsu Province, China. Jpn J Cancer Res. 1999; 90:614-21.

60. Gao Y, Hu N, Han XY, Ding T, Giffen C, Goldstein AM, et al. Risk factors for esophageal and gastric cancers in Shanxi Province, China: a case–control study. Cancer epidemiology. 2011; 35:e91-e9.

61. Goh K-L, Cheah P-L, Md N, Quek K-F, Parasakthi N. Ethnicity and H. pylori as risk factors for gastric cancer in Malaysia: A prospective case control study. Am J Gastroenterol. 2007; 102:40.

62. Goldbohm RA, Hertog MG, Brants HA, van Poppel G, van den Brandt PA. Consumption of black tea and cancer risk: a prospective cohort study. J Natl Cancer Inst. 1996; 88:93-100.

63. Gómez Zuleta M, Otero Regino W, Ruiz Lobo X. Factores de riesgo para cáncer gástrico en pacientes colombianos. Rev Colomb Gastroenterol. 2009; 24.

64. Gunathilake M, Lee J, Jang A, Choi I, Kim Y-I, Kim J. Physical Activity and Gastric Cancer Risk in Patients with and without Helicobacter pylori Infection in A Korean Population: A Hospital-Based Case-Control Study. Cancers. 2018; 10:369.

65. Gwack J, Shin A, Kim C-S, Ko K, Kim Y, Jun J, et al. CagA-producing Helicobacter pylori and increased risk of gastric cancer: a nested case–control study in Korea. Br J Cancer. 2006; 95:639.

66. Hamada GS, Kowalski LP, Nishimoto IN, Rodrigues JJG, Iriya K, Sasazuki S, et al. Risk factors for stomach cancer in Brazil (II): a case-control study among Japanese Brazilians in Sao Paulo. Jpn J Cancer Res. 2002; 32:284-90.

67. Hansen S, Melby K, Aase S, Jellum E, Vollset S. Helicobacter pylori infection and risk of cardia cancer and non-cardia gastric cancer: a nested case-control study. Scand J Gastroenterol. 1999; 34:353-60.

68. Hansson LE, Nyrén O, Bergström R, Wolk A, Lindgren A, Baron J, et al. Diet and risk of gastric cancer. A population-based case-control study in Sweden. International journal of cancer. 1993; 55:181-9.

69. Hansson L-E, Engstrand L, Nyrén O, Evans Jr DJ, Lindgren A, Bergström R, et al. Helicobacter pylori infection: independent risk indicator of gastric adenocarcinoma. Gastroenterology. 1993; 105:1098-103.

70. Hansson LE, Baron J, Nyrén O, Bergström R, Wolk A, Adami HO. Tobacco, alcohol and the risk of gastric cancer. A population-based case-control study in Sweden. International journal of cancer. 1994; 57:26-31.

71. Haruma K, Komoto K, Kamada T, Ito M, Kitadai Y, Yoshihara M, et al. Helicobacter pylori infection is a major risk factor for gastric carcinoma in young patients. Scand J Gastroenterol. 2000; 35:255-9.

72. Held M, Engstrand L, Hansson LE, Bergström R, Wadström T, Nyrén O. Is the association between Helicobacter pylori and gastric cancer confined to CagA-positive strains? Helicobacter. 2004; 9:271-7.

73. Hirohata T, Kono S. Diet/nutrition and stomach cancer in Japan. International journal of cancer. 1997; 71:34-6.

74. Hishida A, Matsuo K, Goto Y, Naito M, Wakai K, Tajima K, et al. Smoking behavior and risk of Helicobacter pylori infection, gastric atrophy and gastric cancer in Japanese. Asian Pac J Cancer Prev. 2010; 11:669-73.

75. Hoang BV, Lee J, Choi IJ, Kim Y-W, Ryu KW, Kim J. Effect of dietary vitamin C on gastric cancer risk in the Korean population. World J Gastroenterol. 2016; 22:6257.

76. Hoey J, Montvernay C, Lambert R. Wine and tobacco: risk factors for gastric cancer in France. Am J Epidemiol. 1981; 113:668-74.

77. Hoshiyama Y, Sasaba T. A case-control study of stomach cancer and its relation to diet, cigarettes, and alcohol consumption in Saitama Prefecture, Japan. Cancer Causes Control. 1992; 3:441-8.

78. Hoshiyama Y, Sasaba T. A case-control study of single and multiple stomach cancers in Saitama Prefecture, Japan. Jpn J Cancer Res. 1992; 83:937-43.

79. Hoshiyama Y, Kawaguchi T, Miura Y, Mizoue T, Tokui N, Yatsuya H, et al. A nested case–control study of stomach cancer in relation to green tea consumption in Japan. Br J Cancer. 2004; 90:135.

80. Huang X, Tajima K, Hamajima N, Inoue M, Takezaki T, Kuroishi T, et al. Effect of life styles on the risk of sybsute-specific gastric cancer in those with and without family history. J Epidemiol. 1999; 9:40-5.

81. Huang XE, Tajima K, Hamajima N, Xiang J, Inoue M, Hirose K, et al. Comparison of lifestyle and risk factors among Japanese with and without gastric cancer family history. International journal of cancer. 2000; 86:421-4.

82. Huerta JM, Chirlaque MD, Molina AJ, Amiano P, Martín V, Fernández-Villa T, et al. Physical activity domains and risk of gastric adenocarcinoma in the MCC-Spain case-control study. PloS one. 2017; 12:e0179731.

83. Icli F, Akbulut H, Yalcin B, Ozdemir F, Isıkdogan A, Hayran M, et al. Education, economic status and other risk factors in gastric cancer:"a case–control study of Turkish oncology group". Med Oncol. 2011; 28:112-20.

84. Inoue M, Tajima K, Hirose K, Kuroishi T, Gao CM, Kitoh T. Life-style and subsite of gastric cancer—joint effect of smoking and drinking habits. International journal of cancer. 1994; 56:494-9.

85. Inoue M, Tajima K, Hirose K, Hamajima N, Takezaki T, Kuroishi T, et al. Tea and coffee consumption and the risk of digestive tract cancers: data from a comparative case-referent study in Japan. Cancer Causes Control. 1998; 9:209-16.

86. Inoue M, Ito LS, Tajima K, Yamamura Y, Kodera Y, Takezaki T, et al. Height, weight, menstrual and reproductive factors and risk of gastric cancer among Japanese postmenopausal women: analysis by subsite and histologic subtype. International journal of cancer. 2002; 97:833-8.

87. Inoue M, Yamamoto S, Kurahashi N, Iwasaki M, Sasazuki S, Tsugane S, et al. Daily total physical activity level and total cancer risk in men and women: results from a large-scale population-based cohort study in Japan. Am J Epidemiol. 2008; 168:391-403.

88. Ito LS, Inoue M, Tajima K, Yamamura Y, Kodera Y, Hirose K, et al. Dietary factors and the risk of gastric cancer among Japanese women: a comparison between the differentiated and non-differentiated subtypes. Ann Epidemiol. 2003; 13:24-31.

89. Jang J, Cho E-J, Hwang Y, Weiderpass E, Ahn C, Choi J, et al. Association between Body Mass Index and Gastric Cancer Risk According to Effect Modification by Helicobacter pylori Infection. Cancer Res Treat. 2018.

90. Jayalekshmi PA, Hassani S, Nandakumar A, Koriyama C, Sebastian P, Akiba S. Gastric cancer risk in relation to tobacco use and alcohol drinking in Kerala, India-Karunagappally cohort study. World J Gastroenterol. 2015; 21:12676.

91. Jedrychowski W, Wahrendorf J, Popiela T, Rachtan J. A case-control study of dietary factors and stomach cancer risk in Poland. International journal of cancer. 1986; 37:837-42.

92. Jedrychowski W, Boeing H, Wahrendorf J, Popiela T, Tobiasz-Adamczyk B, Kulig J. Vodka consumption, tobacco smoking and risk of gastric cancer in Poland. Int J Epidemiol. 1993; 22:606-13.

93. Ji BT, Chow WH, Yang G, McLaughlin JK, Gao RN, Zheng W, et al. The influence of cigarette smoking, alcohol, and green tea consumption on the risk of carcinoma of the cardia and distal stomach in Shanghai, China. Cancer. 1996; 77:2449-57.

94. Kabat GC, Ng SK, Wynder EL. Tobacco, alcohol intake, and diet in relation to adenocarcinoma of the esophagus and gastric cardia. Cancer Causes Control. 1993; 4:123-32.

95. Kamangar F, Dawsey SM, Blaser MJ, Perez-Perez GI, Pietinen P, Newschaffer CJ, et al. Opposing risks of gastric cardia and noncardia gastric adenocarcinomas associated with Helicobacter pylori seropositivity. J Natl Cancer Inst. 2006; 98:1445-52.

96. Karagulle M, Fidan E, Kavgaci H, Ozdemir F. The effects of environmental and dietary factors on the development of gastric cancer. J BUON. 2014; 19:1076-82.

97. Kato I, Tominaga S, Ito Y, Kobayashi S, Yoshii Y, Matsuura A, et al. A comparative case-control analysis of stomach cancer and atrophic gastritis. Cancer Res. 1990; 50:6559-64.

98. Kato M, Asaka M, Shimizu Y, Nobuta A, Takeda H, Sugiyama T, et al. Relationship between Helicobacter pylori infection and the prevalence, site and histological type of gastric cancer. Aliment Pharmacol Ther. 2004; 20:85-9.

99. Keck JW, Miernyk KM, Bulkow LR, Kelly JJ, McMahon BJ, Sacco F, et al. Helicobacter pylori infection and markers of gastric cancer risk in Alaska Native persons: a retrospective case-control study. Can J Gastroenterol Hepatol. 2014; 28:305-10.

100. Keszei A, Schouten L, Goldbohm R, Van Den Brandt P. Red and processed meat consumption and the risk of esophageal and gastric cancer subtypes in The Netherlands Cohort Study. Ann Epidemiol. 2012; 23:2319-26.

101. Kikuchi S, Nakajima T, Kobayashi O, Yamazaki T, Kikuichi M, Mori K, et al. U-shaped effect of drinking and linear effect of smoking on risk for stomach cancer in Japan. Jpn J Cancer Res. 2002; 93:953-9.

102. Kim HY, Cho BD, Chang WK, Kim DJ, Kim YB, Park CK, et al. Helicobacter pylori infection and the risk of gastric cancer among the Korean population. Journal of gastroenterology and hepatology. 1997; 12:100-3.

103. Kim HJ, Chang WK, Kim MK, Lee SS, Choi BY. Dietary factors and gastric cancer in Korea: A case-control study. International journal of cancer. 2002; 97:531-5.

104. Kim D-S, Lee M-S, Kim Y-S, Kim D-H, Bae J-M, Shin M-H, et al. Effect modification by vitamin C on the relation between gastric cancer and Helicobacter pylori. Eur J Epidemiol. 2005; 20:67-71.

105. Kim J, Park S, Nam B-H. Gastric cancer and salt preference: a population-based cohort study in Korea. Am J Clin Nutr. 2010; 91:1289-93.

106. Kim HJ, Kim N, Kim HY, Lee HS, Yoon H, Shin CM, et al. Relationship between body mass index and the risk of early gastric cancer and dysplasia regardless of Helicobacter pylori infection. Gastric Cancer. 2015; 18:762-73.

107. Ko K-P, Park SK, Yang JJ, Ma SH, Gwack J, Shin A, et al. Intake of soy products and other foods and gastric cancer risk: a prospective study. J Epidemiol. 2013; 23:337-43.

108. Komoto K, Haruma K, Kamada T, Tanaka S, Yoshihara M, Sumii K, et al. Helicobacter pylori infection and gastric neoplasia: correlations with histological gastritis and tumor histology. Am J Gastroenterol. 1998; 93:1271-6.

109. Kono S, Ikeda M, Tokudome S, Kuratsune M. A case-control study of gastric cancer and diet in northern Kyushu, Japan. Jpn J Cancer Res. 1988; 79:1067-74.

110. Kuriyama S, Tsubono Y, Hozawa A, Shimazu T, Suzuki Y, Koizumi Y, et al. Obesity and risk of cancer in Japan. International journal of cancer. 2005; 113:148-57.

111. La Vecchia C, Negri E, Decarli A, D'Avanzo B, Franceschi S. A case-control study of diet and gastric cancer in northern Italy. International journal of cancer. 1987; 40:484-9.

112. La Vecchia C, Negri E, Decarli A, D'Avanzo B, Gallotti L, Gentile A, et al. A case-control study of diet and colo-rectal cancer in northern Italy. International journal of cancer. 1988; 41:492-8.

113. La Vecchia C, Muñoz SE, Braga C, Fernandez E, Decarli A. Diet diversity and gastric cancer. International journal of cancer. 1997; 72:255-7.

114. Lagergren J, Bergström R, Lindgren A, Nyrén O. The role of tobacco, snuff and alcohol use in the aetiology of cancer of the oesophagus and gastric cardia. International journal of cancer. 2000; 85:340-6.

115. Lai HTM, Koriyama C, Tokudome S, Tran HH, Tran LT, Nandakumar A, et al. Waterpipe tobacco smoking and gastric cancer risk among Vietnamese men. PloS one. 2016; 11:e0165587.

116. Larsson SC, Giovannucci E, Wolk A. Coffee consumption and stomach cancer risk in a cohort of Swedish women. International journal of cancer. 2006; 119:2186-9.

117. Lazarević K, Nagorni A, Bogdanović D, Rančić N, Stošić L, Milutinović S. Dietary micronutrients and gastric cancer: hospital based study. Cent Eur J Med. 2011; 6:783.

118. Lee J-K, Park B-J, Yoo K-Y, Ahn Y-O. Dietary factors and stomach cancer: a case-control study in Korea. Int J Epidemiol. 1995; 24:33-41.

119. Lee BM, Jang JJ, Kim JS, You YC, Chun SA, Kim HS, et al. Association of Helicobacter pylori infection with gastric adenocarcinoma. Jpn J Cancer Res. 1998; 89:597-603.

120. Lee SA, Kang D, Hong WS, Shim KN, Choe JW, Choi H. Dietary habit and Helicobacter pylori infection in early gastric cancer patient. Cancer Res Treat. 2002; 34:104-10.

121. Lee S-A, Kang D, Shim K, Choe J, Hong W, Choi H. Original article effect of diet and Helicobacter pylori infection to the risk of early gastric cancer. J Epidemiol. 2003; 13:162-8.

122. Levi Z, Kark JD, Shamiss A, Derazne E, Tzur D, Keinan-Boker L, et al. Body mass index and socioeconomic status measured in adolescence, country of origin, and the incidence of gastroesophageal adenocarcinoma in a cohort of 1 million men. Cancer. 2013; 119:4086-93.

123. Li WQ, Kuriyama S, Li Q, Nagai M, Hozawa A, Nishino Y, et al. Citrus consumption and cancer incidence: the Ohsaki cohort study. International journal of cancer. 2010; 127:1913-22.

124. Limburg PJ, Qiao Y-L, Mark SD, Wang G-Q, Perez-Perez GI, Blaser MJ, et al. Helicobacter pylori seropositivity and subsite-specific gastric cancer risks in Linxian, China. J Natl Cancer Inst. 2001; 93:226-33.

125. Lin S-H, Li Y-H, Leung K, Huang C-Y, Wang X-R. Salt processed food and gastric cancer in a Chinese population. Asian Pac J Cancer Prev. 2014; 15:5293-8.

126. Lindblad M, Rodríguez LAG, Lagergren J. Body mass, tobacco and alcohol and risk of esophageal, gastric cardia, and gastric non-cardia adenocarcinoma among men and women in a nested case-control study. Cancer Causes Control. 2005; 16:285-94.

127. Lissowska J, Gail MH, Pee D, Groves FD, Sobin LH, Nasierowska-Guttmejer A, et al. Diet and stomach cancer risk in Warsaw, Poland. Nutr Cancer. 2004; 48:149-59.

128. López-Carrillo L, Torres-López J, Galván-Portillo M, Munoz L, López-Cervantes M. Helicobacter pylori-CagA seropositivity and nitrite and ascorbic acid food intake as predictors for gastric cancer. Eur J Cancer. 2004; 40:1752-9.

129. Ma S-H, Jung W, Weiderpass E, Jang J, Hwang Y, Ahn C, et al. Impact of alcohol drinking on gastric cancer development according to Helicobacter pylori infection status. Br J Cancer. 2015; 113:1381.

130. Machida-Montani A, Sasazuki S, Inoue M, Natsukawa S, Shaura K, Koizumi Y, et al. Association of Helicobacter pylori infection and environmental factors in non-cardia gastric cancer in Japan. Gastric Cancer. 2004; 7:46-53.

131. Máchová L, Čížek L, Horáková D, Koutná J, Lorenc J, Janoutová G, et al. Association between obesity and cancer incidence in the population of the District Sumperk, Czech Republic. Oncol Res Treat. 2007; 30:538-42.

132. Maeda S, Yoshida H, Ogura K, Yamaji Y, Ikenoue T, Mitsushima T, et al. Assessment of gastric carcinoma risk associated with Helicobacter pylori may vary depending on the antigen used: CagA specific enzyme-linked immunoadsorbent assay (ELISA) versus commercially available H. pylori ELISAs. Cancer. 2000; 88:1530-5.

133. Mao Y, Hu J, Semenciw R, White K, Group CCRER. Active and passive smoking and the risk of stomach cancer, by subsite, in Canada. European journal of cancer prevention : the official journal of the European Cancer Prevention Organisation (ECP). 2002; 11:27-38.

134. Mao XQ, Jia XF, Zhou G, Li L, Niu H, Li FL, et al. Green tea drinking habits and gastric cancer in southwest China. Asian Pac J Cancer Prev. 2011; 12:2179-82.

135. Mathew A, Gangadharan P, Varghese C, Nair M. Diet and stomach cancer: a case-control study in South India. European journal of cancer prevention : the official journal of the European Cancer Prevention Organisation (ECP). 2000; 9:89-97.

136. Meine GC, Rota C, Dietz J, Sekine S, Prolla JC. Relationship between caga-positive Helicobacter pylori infection and risk of gastric cancer: a case control study in Porto Alegre, RS, Brazil. Arq Gastroenterol. 2011; 48:41-5.

137. Merry AH, Schouten LJ, Goldbohm RA, van den Brandt PA. Body mass index, height and risk of adenocarcinoma of the oesophagus and gastric cardia: a prospective cohort study. Gut. 2007.

138. Minami Y, Tateno H. Associations between cigarette smoking and the risk of four leading cancers in Miyagi Prefecture, Japan: a multi-site case-control study. Cancer science. 2003; 94:540-7.

139. Moller H, Mellemgaard A, Lindvig K, Olsen JH. Obesity and cancer risk: a Danish record-linkage study. Eur J Cancer. 1994; 30a:344-50.

140. Moy KA, Fan Y, Wang R, Gao Y-T, Mimi CY, Yuan J-M. Alcohol and tobacco use in relation to gastric cancer: a prospective study of men in Shanghai, China. Cancer Epidemiol Biomarkers Prev. 2010:1055-9965. EPI-10-0362.

141. Mu LN, Lu QY, Yu SZ, Jiang QW, Cao W, You NC, et al. Green tea drinking and multigenetic index on the risk of stomach cancer in a Chinese population. International journal of cancer. 2005; 116:972-83.

142. Muñoz N, Plummer M, Vivas J, Moreno V, De Sanjosé S, Lopez G, et al. A case-control study of gastric cancer in Venezuela. International journal of cancer. 2001; 93:417-23.

143. Nagano J, Kono S, Preston DL, Mabuchi K. A prospective study of green tea consumption and cancer incidence, Hiroshima and Nagasaki (Japan). Cancer Causes Control. 2001; 12:501-8.

144. Nakaya N, Tsubono Y, Kuriyama S, Hozawa A, Shimazu T, Kurashima K, et al. Alcohol consumption and the risk of cancer in Japanese men: the Miyagi cohort study. European journal of cancer prevention : the official journal of the European Cancer Prevention Organisation (ECP). 2005; 14:169-74.

145. Navarro Silvera SA, Mayne ST, Risch H, Gammon MD, Vaughan TL, Chow WH, et al. Food group intake and risk of subtypes of esophageal and gastric cancer. International journal of cancer. 2008; 123:852-60.

146. Nishimoto IN, Hamada GS, Kowalski LP, Rodrigues JG, Iriya K, Sasazuki S, et al. Risk factors for stomach cancer in Brazil (I): a case-control study among non-Japanese Brazilians in Sao Paulo. Jpn J Cancer Res. 2002; 32:277-83.

147. Nomura A, Grove JS, Stemmermann GN, Severson RK. A prospective study of stomach cancer and its relation to diet, cigarettes, and alcohol consumption. Cancer Res. 1990; 50:627-31.

148. Nomura A, Stemmermann GN, Chyou P-H, Kato I, Perez-Perez GI, Blaser MJ. Helicobacter pylori infection and gastric carcinoma among Japanese Americans in Hawaii. N Engl J Med. 1991; 325:1132-6.

149. Nomura AM, Lee J, Stemmermann GN, Nomura RY, Perez-Perez GI, Blaser MJ. Helicobacter pylori CagA seropositivity and gastric carcinoma risk in a Japanese American population. J Infect Dis. 2002; 186:1138-44.

150. Nomura AM, Kolonel LN, Miki K, Stemmermann GN, Wilkens LR, Goodman MT, et al. Helicobacter pylori, pepsinogen, and gastric adenocarcinoma in Hawaii. J Infect Dis. 2005; 191:2075-81.

151. Nomura AM, Wilkens LR, Henderson BE, Epplein M, Kolonel LN. The association of cigarette smoking with gastric cancer: the multiethnic cohort study. Cancer Causes Control. 2012; 23:51-8.

152. Ogimoto I, Yoshimura T, Ikeda M. Reproductive Life History as Gastric Cancer Risk for Females: Case-Control Study. J Epidemiol. 1995; 5:177-85.

153. Pakseresht M, Forman D, Malekzadeh R, Yazdanbod A, West RM, Greenwood DC, et al. Dietary habits and gastric cancer risk in north-west Iran. Cancer Causes Control. 2011; 22:725-36.

154. Palli D, Bianchi S, Decarli A, Cipriani F, Avellini C, Cocco P, et al. A case-control study of cancers of the gastric cardia in Italy. Br J Cancer. 1992; 65:263.

155. Palli D, Russo A, Decarli A. Dietary patterns, nutrient intake and gastric cancer in a high-risk area of Italy. Cancer Causes Control. 2001; 12:163-72.

156. Palli D, Russo A, Ottini L, Masala G, Saieva C, Amorosi A, et al. Red meat, family history, and increased risk of gastric cancer with microsatellite instability. Cancer Res. 2001; 61:5415-9.

157. Parent M-É, Rousseau M-C, El-Zein M, Latreille B, Désy M, Siemiatycki J. Occupational and recreational physical activity during adult life and the risk of cancer among men. Cancer epidemiology. 2011; 35:151-9.

158. Parsonnet J, Friedman GD, Vandersteen DP, Chang Y, Vogelman JH, Orentreich N, et al. Helicobacter pylori infection and the risk of gastric carcinoma. N Engl J Med. 1991; 325:1127-31.

159. Parsonnet J, Friedman G, Orentreich N, Vogelman H. Risk for gastric cancer in people with CagA positive or CagA negative Helicobacter pylori infection. Gut. 1997; 40:297-301.

160. Peleteiro B, Lunet N, Barros R, La Vecchia C, Barros H. Factors contributing to the underestimation of Helicobacter pylori-associated gastric cancer risk in a high-prevalence population. Cancer Causes Control. 2010; 21:1257-64.

161. Persson C, Sasazuki S, Inoue M, Kurahashi N, Iwasaki M, Miura T, et al. Plasma levels of carotenoids, retinol and tocopherol and the risk of gastric cancer in Japan: a nested case–control study. Carcinogenesis. 2008; 29:1042-8.

162. Persson C, Jia Y, Pettersson H, Dillner J, Nyrén O, Ye W. H. pylori seropositivity before age 40 and subsequent risk of stomach cancer: a glimpse of the true relationship? PloS one. 2011; 6:e17404.

163. Phukan RK, Narain K, Zomawia E, Hazarika NC, Mahanta J. Dietary habits and stomach cancer in Mizoram, India. J Gastroenterol. 2006; 41:418-24.

164. Pourfarzi F, Whelan A, Kaldor J, Malekzadeh R. The role of diet and other environmental factors in the causation of gastric cancer in Iran—a population based study. International journal of cancer. 2009; 125:1953-60.

165. Queiroz DM, Mendes EN, Rocha GA, Oliveira AM, Oliveira CA, Cabral MM, et al. Serological and direct diagnosis of Helicobacter pylori in gastric carcinoma: a case-control study. J Med Microbiol. 1999; 48:501-6.

166. Ramón JM, Serra L, Cerdó C, Oromí J. Dietary factors and gastric cancer risk. A case-control study in Spain. Cancer. 1993; 71:1731-5.

167. Ramos MFKP, Ribeiro Júnior U, Viscondi JKY, Zilberstein B, Cecconello I, Eluf-Neto J. Risk factors associated with the development of gastric cancer—case-control study. Rev Colomb Gastroenterol. 2018; 64:611-9.

168. Rao DN, Ganesh B, Dinshaw KA, Mohandas KM. A case-control study of stomach cancer in Mumbai, India. International journal of cancer. 2002; 99:727-31.

169. Rapp K, Schroeder J, Klenk J, Stoehr S, Ulmer H, Concin H, et al. Obesity and incidence of cancer: a large cohort study of over 145 000 adults in Austria. Br J Cancer. 2005; 93:1062.

170. Rudi J, Müller M, Von Herbay A, Zuna I, Raedsch R, Stremmel W, et al. Lack of association of Helicobacter pylori seroprevalence and gastric cancer in a population with low gastric cancer incidence. Scand J Gastroenterol. 1995; 30:958-63.

171. Rudi J, Kolb C, Maiwald M, Zuna I, Von Herbay A, Galle PR, et al. Serum antibodies against Helicobacter pylori proteins VacA and CagA are associated with increased risk for gastric adenocarcinoma. Dig Dis Sci. 1997; 42:1652-9.

172. Rugge M, Busatto G, Cassaro M, Shiao YH, Russo V, Leandro G, et al. Patients younger than 40 years with gastric carcinoma. Cancer. 1999; 85:2506-11.

173. Saha SK. Smoking habits and carcinoma of the stomach: a case-control study. Jpn J Cancer Res. 1991; 82:497-502.

174. Samanic C, Chow W-H, Gridley G, Jarvholm B, Fraumeni JF. Relation of body mass index to cancer risk in 362,552 Swedish men. Cancer Causes Control. 2006; 17:901-9.

175. Sarker KK, Kabir MJ, uddin Bhuyian AM, Alam MS, Chowdhury FR, Ahad MA, et al. H. pylori infection and gastric cancer in Bangladesh: a case-control study. Int J Surg Oncol. 2017; 2:e44.

176. Sasazuki S, Sasaki S, Tsugane S, Group JPHCS. Cigarette smoking, alcohol consumption and subsequent gastric cancer risk by subsite and histologic type. International journal of cancer. 2002; 101:560-6.

177. Sasazuki S, Inoue M, Hanaoka T, Yamamoto S, Sobue T, Tsugane S. Green tea consumption and subsequent risk of gastric cancer by subsite: the JPHC Study. Cancer Causes Control. 2004; 15:483-91.

178. Sasazuki S, Inoue M, Iwasaki M, Otani T, Yamamoto S, Ikeda S, et al. Effect of Helicobacter pylori infection combined with CagA and pepsinogen status on gastric cancer development among Japanese men and women: a nested case-control study. Cancer Epidemiol Biomarkers Prev. 2006; 15:1341-7.

179. Sekikawa T, Masaki M, Nakamura KI. Relation of gastric cancer with helicobacter pylori infection and dietary habits. Journal of the Showa Medical Association. 1998; 58:479-86.

180. Setiawan VW, Zhang ZF, Yu GP, Lu QY, Li YL, Lu ML, et al. Protective effect of green tea on the risks of chronic gastritis and stomach cancer. International journal of cancer. 2001; 92:600-4.

181. Siman J, Forsgren A, Berglund G, Florén C-H. Association between Helicobacter pylori and gastric carcinoma in the city of Malmö, Sweden: a prospective study. Scand J Gastroenterol. 1997; 32:1215-21.

182. Simán JH, Engstrand L, Berglund G, Forsgren A, Florén C-H. Helicobacter pylori and CagA seropositivity and its association with gastric and oesophageal carcinoma. Scand J Gastroenterol. 2007; 42:933-40.

183. Sjödahl K, Jia C, Vatten L, Nilsen T, Hveem K, Lagergren J. Body mass and physical activity and risk of gastric cancer in a population-based cohort study in Norway. Cancer Epidemiol Biomarkers Prev. 2008; 17:135-40.

184. Sjödahl K, Jia C, Vatten L, Nilsen T, Hveem K, Lagergren J. Salt and gastric adenocarcinoma: a population-based cohort study in Norway. Cancer Epidemiol Biomarkers Prev. 2008; 17:1997-2001.

185. Somi MH, Mousavi SM, Naghashi S, Faramarzi E, Jafarabadi MA, Ghojazade M, et al. Is there any relationship between food habits in the last two decades and gastric cancer in North-Western Iran. Asian Pac J Cancer Prev. 2015; 16:283-90.

186. Sriamporn S, Setiawan V, Pisani P, Suwanrungruang K, Sirijaichingkul S, Mairiang P, et al. Gastric cancer: the roles of diet, alcohol drinking, smoking and Helicobacter pylori in Northeastern Thailand. Asian Pac J Cancer Prev. 2002; 3:52.

187. Steevens J, Schouten LJ, Goldbohm RA, van den Brandt PA. Alcohol consumption, cigarette smoking and risk of subtypes of oesophageal and gastric cancer: a prospective cohort study. Gut. 2010; 59:39-48.

188. Stemmermann GN, Nomura AM, Chyou P-H, Yoshizawa C. Prospective study of alcohol intake and large bowel cancer. Dig Dis Sci. 1990; 35:1414-20.

189. Strumylaitė L, Žičkutė J, Dudzevičius JE, Dregval L. Salt-preserved foods and risk of gastric cancer. Medicina. 2006; 42:164-70.

190. Sun C-Q, Chang Y-B, Cui L-L, Chen J-J, Sun N, Zhang W-J, et al. A population-based case-control study on risk factors for gastric cardia cancer in rural areas of Linzhou. Asian Pac J Cancer Prev. 2013; 14:2897-901.

191. Suwanrungruang K, Sriamporn S, Wiangnon S, Rangsrikajee D, Sookprasert A, Thipsuntornsak N, et al. Lifestyle-related risk factors for stomach cancer in northeast Thailand. Asian Pac J Cancer Prev. 2008; 9:71-5.

192. Takahashi M, Hasegawa R. Enhancing effects of dietary salt on both initiation and promotion stages of rat gastric carcinogenesis. Princess Takamatsu symposia. 1985; 16:169-82.

193. Talley NJ, Zinsmeister AR, Weaver A, DiMagno EP, Carpenter HA, Perez-Perez GI, et al. Gastric adenocarcinoma and Helicobacter pylori infection. J Natl Cancer Inst. 1991; 83:1734-9.

194. Terry P, Nyrén O, Yuen J. Protective effect of fruits and vegetables on stomach cancer in a cohort of Swedish twins. International journal of cancer. 1998; 76:35-7.

195. Terry P, Lagergren J, Ye W, Nyrén O, Wolk A. Antioxidants and cancers of the esophagus and gastric cardia. International journal of cancer. 2000; 87:750-4.

196. Tønnesen H, Møller H, Andersen J, Jensen E, Juel K. Cancer morbidity in alcohol abusers. Br J Cancer. 1994; 69:327.

197. Tran GD, Sun XD, Abnet CC, Fan JH, Dawsey SM, Dong ZW, et al. Prospective study of risk factors for esophageal and gastric cancers in the Linxian general population trial cohort in China. International journal of cancer. 2005; 113:456-63.

198. Tsubono Y, Nishino Y, Komatsu S, Hsieh C-C, Kanemura S, Tsuji I, et al. Green tea and the risk of gastric cancer in Japan. N Engl J Med. 2001; 344:632-6.

199. Tsugane S, Sasazuki S, Kobayashi M, Sasaki S. Salt and salted food intake and subsequent risk of gastric cancer among middle-aged Japanese men and women. Br J Cancer. 2004; 90:128.

200. Turns A, Péqljignot G, Gignoux M, Valla A. Cancers of the digestive tract, alcohol and tobacco. International journal of cancer. 1982; 30:9-11.

201. Wang X-Q, Yan H, Terry PD, Wang J-S, Cheng L, Wu W-A, et al. Interactions between CagA and smoking in gastric cancer. World J Gastroenterol. 2011; 17:3330.

202. Wang X-Q, Yan H, Terry PD, Wang J-S, Cheng L, Wu W-A, et al. Interaction between dietary factors and Helicobacter pylori infection in noncardia gastric cancer: a population-based case-control study in China. J Am Coll Nutr. 2012; 31:375-84.

203. Wang Y, Duan H, Yang H. A case-control study of stomach cancer in relation to Camellia sinensis in China. Surg Oncol. 2015; 24:67-70.

204. Wang T, Cai H, Sasazuki S, Tsugane S, Zheng W, Cho ER, et al. Fruit and vegetable consumption, Helicobacter pylori antibodies, and gastric cancer risk: a pooled analysis of prospective studies in China, Japan, and Korea. International journal of cancer. 2017; 140:591-9.

205. Ward MH, Lopez-Carrillo L. Dietary factors and the risk of gastric cancer in Mexico City. Am J Epidemiol. 1999; 149:925-32.

206. Watabe K, Nishi M, Miyake H, Hirata K. Lifestyle and gastric cancer: a case-control study. Oncol Rep. 1998; 5:1191-5.

207. Watanabe Y, Kurata JH, Mizuno S, Mukai M, Inokuchi H, Miki K, et al. Helicobacter pylori infection and gastric cancer (A nested case-control study in a rural area of Japan). Dig Dis Sci. 1997; 42:1383-7.

208. Webb PM, Yu MC, Forman D, Henderson BE, Newell DG, Yuan JM, et al. An apparent lack of association between Helicobacter pylori infection and risk of gastric cancer in China. International journal of cancer. 1996; 67:603-7.

209. Wen X, Song F-m. Salt taste sensitivity, physical activity and gastric cancer. Asian Pac J Cancer Prev. 2010; 11:1473-7.

210. Wolk A, Gridley G, Svensson M, Nyren O, McLaughlin JK, Fraumeni JF, et al. A prospective study of obesity and cancer risk (Sweden). Cancer Causes Control. 2001; 12:13-21.

211. Wu M-S, Shun C-T, Lee W, Chen C, Wang H, Lee W, et al. Gastric cancer risk in relation to Helicobacter pylori infection and subtypes of intestinal metaplasia. Br J Cancer. 1998; 78:125.

212. Wu AH, Wan P, Bernstein L. A multiethnic population-based study of smoking, alcohol and body size and risk of adenocarcinomas of the stomach and esophagus (United States). Cancer Causes Control. 2001; 12:721-32.

213. Wu AH, Crabtree JE, Bernstein L, Hawtin P, Cockburn M, Tseng Cc, et al. Role of Helicobacter pylori CagA+ strains and risk of adenocarcinoma of the stomach and esophagus. International journal of cancer. 2003; 103:815-21.

214. Wu I-C, Wu D-C, Yu F-J, Wang J-Y, Kuo C-H, Yang S-F, et al. Association between Helicobacter pylori seropositivity and digestive tract cancers. World J Gastroenterol. 2009; 15:5465.

215. Wu-Williams AH, Mimi CY, Mack TM. Life-style, workplace, and stomach cancer by subsite in young men of Los Angeles County. Cancer Res. 1990; 50:2569-76.

216. Xibin S, Moller H, Evans HS, Dixing D, Wenjie D, Jianbang L. Residential environment, diet and risk of stomach cancer: a case-control study in Linzhou, China. Asian Pac J Cancer Prev. 2002; 3:167-72.

217. Yamaoka Y, Kodama T, Kashima K, Graham D. Antibody against Helicobacter pylori CagA and VacA and the risk for gastric cancer. J Clin Pathol. 1999; 52:215-8.

218. Yang W-G, Chen C-B, Wang Z-X, Liu Y-P, Wen X-Y, Zhang S-F, et al. A case-control study on the relationship between salt intake and salty taste and risk of gastric cancer. World J Gastroenterol. 2011; 17:2049.

219. Yang S, Lee J, Choi IJ, Kim YW, Ryu KW, Sung J, et al. Effects of alcohol consumption, ALDH2 rs671 polymorphism, and Helicobacter pylori infection on the gastric cancer risk in a Korean population. Oncotarget. 2017; 8:6630.

220. Ye W, Ekström AM, Hansson LE, Bergström R, Nyrén O. Tobacco, alcohol and the risk of gastric cancer by sub-site and histologic type. International journal of cancer. 1999; 83:223-9.

221. You W-C, Blot WJ, Chang Y-S, Ershow AG, Yang Z-T, An Q, et al. Diet and high risk of stomach cancer in Shandong, China. Cancer Res. 1988; 48:3518-23.

222. Yu G-p, Hsieh C-c. Risk factors for stomach cancer: a population-based case-control study in Shanghai. Cancer Causes Control. 1991; 2:169-74.

223. Yuan J-M, Mimi CY, Xu W-W, Cockburn M, Gao Y-T, Ross RK. Helicobacter pylori infection and risk of gastric cancer in Shanghai, China: updated results based upon a locally developed and validated assay and further follow-up of the cohort. Cancer Epidemiol Biomarkers Prev. 1999; 8:621-4.

224. Yun YH, Jung KW, Bae J-M, Lee JS, Shin SA, Park SM, et al. Cigarette smoking and cancer incidence risk in adult men: National Health Insurance Corporation Study. Cancer Detect Prev. 2005; 29:15-24.

225. Zamani N, Hajifaraji M, Malekshah A-T, Keshtkar AA, Esmaillzadeh A, Malekzadeh R. A case-control study of the relationship between gastric cancer and meat consumption in Iran. Arch Iran Med. 2013; 16:324-9.

226. Zaridze D, Borisova E, Maximovitch D, Chkhikvadze V. Alcohol Consumption, Smoking and Risk of Gastric Cancer: Case—Control Study from Moscow, Russia. Cancer Causes Control. 2000; 11:363-71.

227. Zendehdel K, Nyrén O, Luo J, Dickman PW, Boffetta P, Englund A, et al. Risk of gastroesophageal cancer among smokers and users of Scandinavian moist snuff. International journal of cancer. 2008; 122:1095-9.

228. Zhang Z-F, Kurtz RC, Sun M, Karpeh M, Yu G-P, Gargon N, et al. Adenocarcinomas of the esophagus and gastric cardia: medical conditions, tobacco, alcohol, and socioeconomic factors. Cancer Epidemiol Biomarkers Prev. 1996; 5:761-8.

229. Zhang YW, Eom SY, Kim YD, Song YJ, Yun HY, Park JS, et al. Effects of dietary factors and the NAT2 acetylator status on gastric cancer in Koreans. International journal of cancer. 2009; 125:139-45.

230. Zhang Z, Zhang X. Salt taste preference, sodium intake and gastric cancer in China. Asian Pac J Cancer Prev. 2011; 12:1207-10.

231. Zhong C, Li K-N, Bi J-W, Wang B-C. Sodium intake, salt taste and gastric cancer risk according to Helicobacter pylori infection, smoking, histological type and tumor site in China. Asian Pac J Cancer Prev. 2012; 13:2481-4.

Study or Subgroup	log[Odds Ratio]	SE	Weight	Odds Ratio IV, Random, 95% CI
Abnet 2011	0.78390157	0.51229948	1.2%	2.19 [0.80, 5.98]
Behnampour 2014	2.92230111	1.24079101	0.4%	18.58 [1.63, 211.50]
Blaster 1995	0.64185387	0.38052422	1.4%	1.90 [0.90, 4.01]
Brenner 2002	0.59277421	0.31300977	1.6%	1.81 [0.98, 3.34]
Brenner 2004	0.83290911	0.33144975	1.6%	2.30 [1.20, 4.40]
Cai 2000	1.23924341	0.68527639	0.8%	3.45 [0.90, 13.23]
Cai 2016	1.02961951	0.11125053	2.0%	2.80 [2.25, 3.48]
Chang 2001	0.59883654	0.25594441	1.8%	1.82 [1.10, 3.01]
Chen 2009	0.98954123	0.36934277	1.5%	2.69 [1.30, 5.55]
Cho 2010	1.14103311	0.12209304	2.0%	3.13 [2.46, 3.98]
Chow 1998	0.26236424	0.30346534	1.6%	1.30 [0.72, 2.36]
Chung 2010	0.53003985	0.08177318	2.1%	1.70 [1.45, 1.99]
Ellison-Loschman 2017	2.49897381	0.34819631	1.5%	12.17 [6.15, 24.08]
Enorth 2000	0.74193728	0.32287407	1.6%	2.10 [1.12, 3.95]
Fang 2015	0.51640999	0.18416485	1.9%	1.68 [1.17, 2.40]
Forman 1998	1.01884731	0.51950611	1.1%	2.77 [1.00, 7.67]
Frenandez de Larrea-Baz 2017	0.27002711	0.24110685	1.8%	1.31 [0.82, 2.10]
Fukuda 1995	0.00796819	0.20213221	1.9%	1.01 [0.68, 1.50]
Goh 2007	0.93137641	0.40180826	1.4%	2.54 [1.15, 5.58]
Gwack 2006	1.31908561	0.62465591	0.9%	3.74 [1.10, 12.72]
Hansen 1999	0.76546788	0.21459191	1.9%	2.15 [1.41, 3.27]
Hansson 1993 B	1.00430171	0.38303319	1.4%	2.73 [1.29, 5.78]
Haruma 2000	3.15700031	0.62957931	0.9%	23.50 [6.84, 80.72]
Held 2004	1.36097661	0.37303069	1.5%	3.90 [1.88, 8.10]
Huerta 2017	0.03292879	0.07113943	2.1%	1.03 [0.90, 1.19]
Kamanger 2006	-1.28013421	1.04785451	0.5%	0.28 [0.04, 2.17]
Kato 2004	1.34781341	0.06140258	2.1%	3.85 [3.41, 4.34]
Keck 2014	0.96698391	0.39176306	1.4%	2.63 [1.22, 5.67]
Kim 1997	0.32930374	0.22621855	1.8%	1.39 [0.89, 2.17]
Kim 2005	0.53649342	0.21060504	1.9%	1.71 [1.13, 2.58]
Kiyuchi 2002	1.35609281	0.17141938	1.9%	3.88 [2.77, 5.43]
Lee 1998	1.64865861	0.26324514	1.7%	5.20 [3.10, 8.71]
Lee 2002	0.93950915	0.45421061	1.3%	2.56 [1.05, 6.23]
Lee 2003	1.66770681	0.57977861	1.0%	5.30 [1.70, 16.51]
Limburge 2001	0.47000363	0.21614742	1.8%	1.60 [1.05, 2.44]
Lopez- Carrillo 2004	0.04974209	0.23366272	1.8%	1.05 [0.66, 1.66]
Machida-Montani 2004	2.10413411	0.40640021	1.4%	8.20 [3.70, 18.19]
Maeda 2000	2.34180571	0.49899557	1.2%	10.40 [3.91, 27.66]
Meine 2011	2.49979521	0.75802416	0.7%	12.18 [2.76, 53.81]
Nomura 2002	0.73716402	0.16844657	1.9%	2.09 [1.50, 2.91]
Nomura 1991	1.79175951	0.53795129	1.1%	6.00 [2.09, 17.22]
Nomura 2005	0.51162529	0.22535153	1.8%	1.67 [1.07, 2.59]
Packseresht 2011	0.44340292	1.51501391	0.3%	1.56 [0.08, 30.35]
Parsonnet 1991	1.28093391	0.35716525	1.5%	3.60 [1.79, 7.25]
Parsonnet 1997	1.35824691	0.37549618	1.5%	3.89 [1.86, 8.12]
Peleteiro 2010	-0.31471071	0.23814604	1.8%	0.73 [0.46, 1.16]
Persson 2011	1.41098691	0.38219702	1.4%	4.10 [1.94, 8.67]
Phukan 2006	0.13976192	0.71770681	0.8%	1.15 [0.28, 4.69]
Queiroz 1999	1.91692271	0.50603331	1.2%	6.80 [2.52, 18.33]
Rudi 1995	0.32930374	0.26967156	1.7%	1.39 [0.82, 2.36]
Rudi 1997	0.32425508	0.17282321	1.9%	1.38 [0.99, 1.94]
Rugge 1999	1.02604161	0.30930844	1.6%	2.79 [1.52, 5.12]
Sarker 2017	1.15618221	0.31469673	1.6%	3.18 [1.71, 5.89]
Sasazuki 2006	1.37498061	0.94233281	0.5%	3.96 [0.62, 25.08]
Sekikawa 1998	1.11841491	0.73812586	0.8%	3.06 [0.72, 13.00]
Siman 1997	1.60943791	0.42191061	1.4%	5.00 [2.19, 11.43]
Siman 2007	2.87919831	0.73462588	0.8%	17.80 [4.22, 75.12]
Tally 1991	0.48858002	0.37005997	1.5%	1.63 [0.79, 3.37]
Wang 2011	1.29143401	0.46152675	1.3%	3.64 [1.47, 8.99]
Wang 2016	1.64400371	0.21612108	1.8%	5.18 [3.39, 7.91]
Watanabe 1997	0.60976559	0.57949018	1.0%	1.84 [0.59, 5.73]
Wu 1998	0.88789129	0.32709822	1.6%	2.43 [1.28, 4.61]
Wu 2003	0.33575773	0.13939823	2.0%	1.40 [1.06, 1.84]
Wu 2009	1.11841491	0.31508139	1.6%	3.06 [1.65, 5.67]
Yamaoka 1998	0.78390157	0.31989369	1.6%	2.19 [1.17, 4.10]
Yang 2017	1.95586051	0.21914031	1.8%	7.07 [4.60, 10.86]
Yuan 1999	0.60976559	0.26981163	1.7%	1.84 [1.08, 3.12]
Zamani 2013	0.18078789	0.24312492	1.8%	1.20 [0.74, 1.93]
**Total (95% CI)**			**100.0%**	**2.56 [2.18, 3.00]**

Heterogeneity: Tau² = 0.31; Chi² = 489.69, df = 67 (P < 0.00001); I² = 86%

Test for overall effect: Z = 11.58 (P < 0.00001)

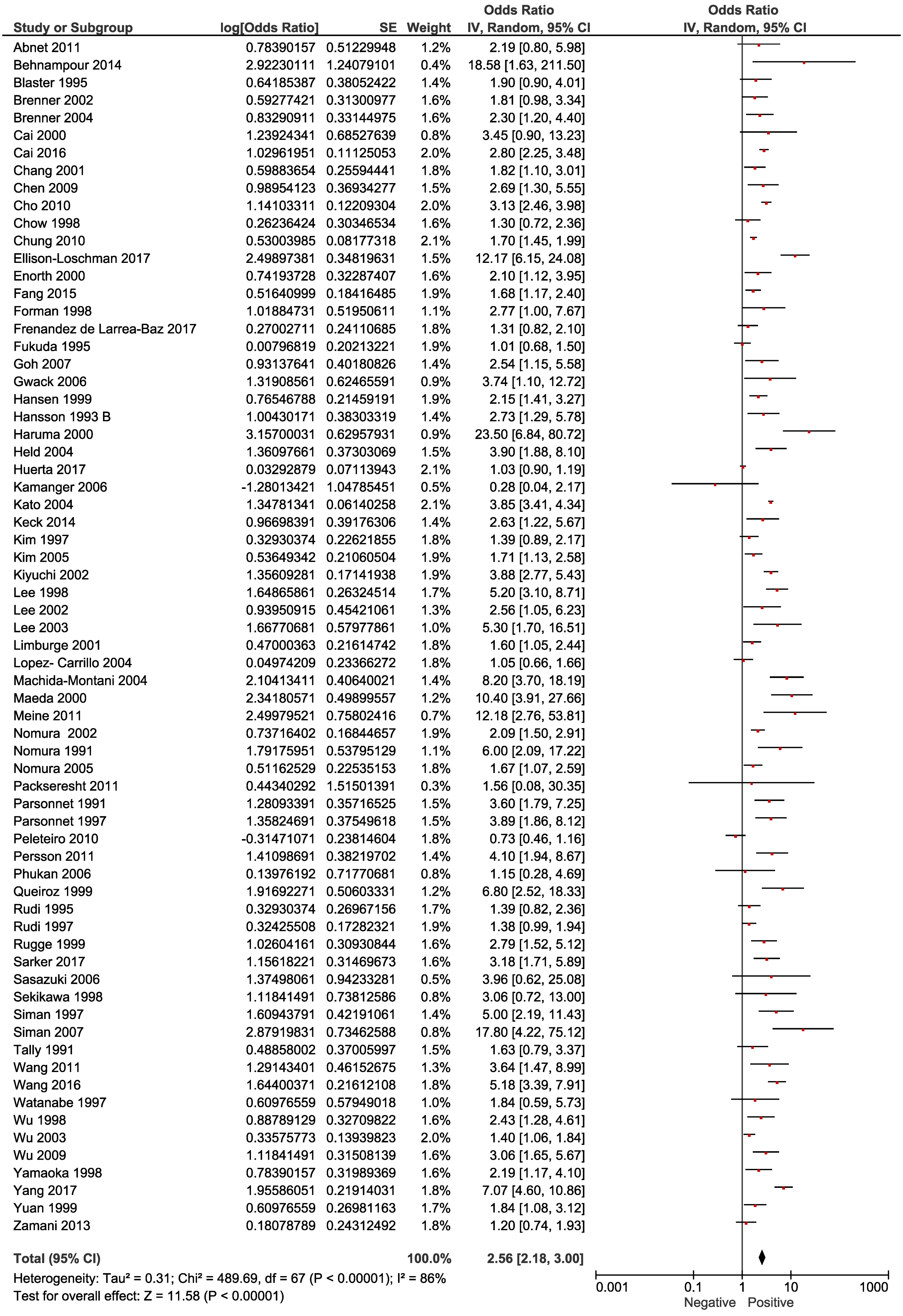

Study or Subgroup	log[Effect size]	SE	Weight	Effect size IV, Random, 95% CI
**Odds Ratio**				
Agudo 1991	-0.07257068	0.23897175	1.1%	0.93 [0.58, 1.49]
Al-qadasi 2016	0.55388511	0.29721478	0.9%	1.74 [0.97, 3.12]
Amadori 1995	0.16381806	0.25917044	1.0%	1.18 [0.71, 1.96]
Asombang 2013	1.97685491	0.84511931	0.2%	7.22 [1.38, 37.84]
Bao 2004	0.39204211	0.16578038	1.3%	1.48 [1.07, 2.05]
Baroudi 2014	1.29746321	0.37059179	0.7%	3.66 [1.77, 7.57]
Brenner 2002	1.06471071	0.37546341	0.7%	2.90 [1.39, 6.05]
Cai 2003	0.42657402	0.20067721	1.2%	1.53 [1.03, 2.27]
Campos 2006	0.18232159	0.22066261	1.1%	1.20 [0.78, 1.85]
Chen 2000	1.09861231	0.41418943	0.6%	3.00 [1.33, 6.76]
Chen 2002	-0.51754797	0.21566084	1.2%	0.60 [0.39, 0.91]
Chen 2009	0.82417542	0.39607543	0.7%	2.28 [1.05, 4.96]
Chew 1999	0.54985404	0.20347561	1.2%	1.73 [1.16, 2.58]
Choi 1991	0.29266962	0.21323192	1.2%	1.34 [0.88, 2.04]
Chung 2010	0.64395696	0.08337975	1.6%	1.90 [1.62, 2.24]
Correa 1985	0.43825489	0.27545306	1.0%	1.55 [0.90, 2.66]
Duan 2009	0.67243415	0.26943409	1.0%	1.96 [1.16, 3.32]
Ellison-Loschman 2017	0.37156358	0.27017385	1.0%	1.45 [0.85, 2.46]
Engel 2003	1.02961951	0.22801478	1.1%	2.80 [1.79, 4.38]
Epplein 2014	0.53680092	0.20897151	1.2%	1.71 [1.14, 2.58]
Fang 2015	0.37156358	0.15830491	1.4%	1.45 [1.06, 1.98]
Fei 2006	1.03460691	0.23076597	1.1%	2.81 [1.79, 4.42]
Gajalkshmi 1996	0.91629076	0.30182901	0.9%	2.50 [1.38, 4.52]
Gammon 1997	0.71245951	0.16241214	1.4%	2.04 [1.48, 2.80]
Gao 2011	-0.19851667	0.13662151	1.5%	0.82 [0.63, 1.07]
Goh 2007	0.92465562	0.36432478	0.7%	2.52 [1.23, 5.15]
Gomez Zuleta 2011	-0.57981849	0.30130953	0.9%	0.56 [0.31, 1.01]
Gwack 2006	0.42488337	0.26054573	1.0%	1.53 [0.92, 2.55]
Hamada 2002	-0.35667497	0.42703481	0.6%	0.70 [0.30, 1.62]
Hansson 1994	0.43825489	0.31914294	0.8%	1.55 [0.83, 2.90]
Haruma 2000	1.05779031	0.42321011	0.6%	2.88 [1.26, 6.60]
Hisihida 2010	0.92425889	0.18682855	1.3%	2.52 [1.75, 3.63]
Hoey 1981	1.56666281	0.73813201	0.3%	4.79 [1.13, 20.36]
Hoshiyama 1992 A	0.58778661	0.23221191	1.1%	1.80 [1.14, 2.84]
Hoshiyama 1992 B	0.06485101	0.28958836	0.9%	1.07 [0.60, 1.88]
Huang 1998	0.79750723	0.14188997	1.4%	2.22 [1.68, 2.93]
Huerta 2017	0.10211068	0.08530123	1.6%	1.11 [0.94, 1.31]
Inoue 1994	0.99325181	0.20749906	1.2%	2.70 [1.80, 4.05]
Inoue 2002	0.73188698	0.13589066	1.5%	2.08 [1.59, 2.71]
Ito 2003	0.51879376	0.12875003	1.5%	1.68 [1.31, 2.16]
Jedrchowski 1993	0.12486901	0.11635548	1.5%	1.13 [0.90, 1.42]
Jedrychowski 1986	-0.38566247	0.28671688	0.9%	0.68 [0.39, 1.19]
Kabat 1992	0.60595393	0.29446298	0.9%	1.83 [1.03, 3.26]
Kato 1990	0.08065789	0.40902013	0.6%	1.08 [0.49, 2.42]
Keszei 2012	0.45563719	0.20880723	1.2%	1.58 [1.05, 2.37]
Kim 2002	0.62379712	0.41048887	0.6%	1.87 [0.83, 4.17]
Kiyuchi 2002	0.12839319	0.13853955	1.4%	1.14 [0.87, 1.49]
Ko 2013	1.09650041	0.18418721	1.3%	2.99 [2.09, 4.30]
Komoto 1998	0.99584103	0.37435571	0.7%	2.71 [1.30, 5.64]
La Vecchia 1987	-0.10997484	0.19680391	1.2%	0.90 [0.61, 1.32]
Lagergren 2000	1.41585311	0.16287878	1.4%	4.12 [2.99, 5.67]
Lai 2016	0.09531021	0.14275911	1.4%	1.10 [0.83, 1.46]
Lazarevic 2011	0.74193728	0.28165991	0.9%	2.10 [1.21, 3.65]
Lee 1995	1.22377551	0.35364652	0.8%	3.40 [1.70, 6.80]
Lee 2003	0.38933569	0.75405831	0.2%	1.48 [0.34, 6.47]
Lindblad 2005	0.53062826	0.09116811	1.6%	1.70 [1.42, 2.03]
Lissowska 2004	0.75340652	0.14087184	1.4%	2.12 [1.61, 2.80]
Machida-Montani 2004	1.02961951	0.43098995	0.6%	2.80 [1.20, 6.52]
Machova 2007	0.22793211	0.13901636	1.4%	1.26 [0.96, 1.65]
Mao 2002	0.17479327	0.14174951	1.4%	1.19 [0.90, 1.57]
Mao 2011	0.58221561	0.22517577	1.1%	1.79 [1.15, 2.78]
Mathew 2000	0.27611542	0.22848484	1.1%	1.32 [0.84, 2.06]
Minami 2003	0.32208351	0.13508107	1.5%	1.38 [1.06, 1.80]
Moura 2014	0.63127178	0.02417134	1.7%	1.88 [1.79, 1.97]
Munoz 2001	0.33647221	0.17682326	1.3%	1.40 [0.99, 1.98]
Navrro Silvera 2008	0.55518544	0.15703855	1.4%	1.74 [1.28, 2.37]
Nishimoto 2002	0.47000363	0.24375291	1.1%	1.60 [0.99, 2.58]
Palli 2001 A	-0.15123098	0.16706745	1.3%	0.86 [0.62, 1.19]
Palli 2001 B	0.10868847	0.25005826	1.0%	1.11 [0.68, 1.82]
Phukan 2006	0.83290911	0.45708151	0.5%	2.30 [0.94, 5.63]
Pourfarzi 2009	-0.34249035	0.28437287	0.9%	0.71 [0.41, 1.24]
Ramos 2018	0.98207849	0.22345743	1.1%	2.67 [1.72, 4.14]
RAO 2002	0.15786745	0.18550441	1.3%	1.17 [0.81, 1.68]
Saha 1991	0.94778937	0.38276216	0.7%	2.58 [1.22, 5.46]
Sjodahl 2008 B	-0.03625637	0.14884152	1.4%	0.96 [0.72, 1.29]
Somi 2015	0.93609333	0.20686996	1.2%	2.55 [1.70, 3.82]
Sriamporn 2003	0.47000363	0.56677806	0.4%	1.60 [0.53, 4.86]
Sun 2013	0.66268802	0.29011482	0.9%	1.94 [1.10, 3.43]
Sunwanrungruang 2008	0.71294981	1.54384031	0.1%	2.04 [0.10, 42.05]
Terry 2000	1.33992601	0.21782477	1.2%	3.82 [2.49, 5.85]
Tuyns 1982	0.00995032	0.51354581	0.5%	1.01 [0.37, 2.76]
Wang 2011	1.68639901	0.34803966	0.8%	5.40 [2.73, 10.68]
Watanabe 1997	0.03922068	0.08333782	1.6%	1.04 [0.88, 1.22]
Webb 1996	0.07696108	0.31450558	0.9%	1.08 [0.58, 2.00]
Wen 2010	0.78024185	0.25700605	1.0%	2.18 [1.32, 3.61]
Wu -william 1990	0.78754783	0.31827885	0.8%	2.20 [1.18, 4.10]
Wu 2001	0.51521599	0.13210231	1.5%	1.67 [1.29, 2.17]
Yang 2011	0.81977981	0.29521117	0.9%	2.27 [1.27, 4.05]
Ye 1999	0.62914187	0.14747345	1.4%	1.88 [1.41, 2.50]
You 1998	0.32930374	0.13891102	1.4%	1.39 [1.06, 1.82]
Yu 1991	0.85057825	0.51654732	0.5%	2.34 [0.85, 6.44]
Zamani 2013	-0.37446901	0.30252835	0.9%	0.69 [0.38, 1.24]
Zaridze 1999	0.33647221	0.22801478	1.1%	1.40 [0.90, 2.19]
Zhang 1996	0.58778661	0.47627628	0.5%	1.80 [0.71, 4.58]
Zhang 2009	0.17395335	0.10431011	1.6%	1.19 [0.97, 1.46]
**Subtotal (95% CI)**			**100.0%**	**1.61 [1.49, 1.75]**
Heterogeneity: Tau² = 0.10; Chi² = 436.08, df = 94 (P < 0.00001); I² = 78%				
Test for overall effect: Z = 11.76 (P < 0.00001)				
**Risk Ratio**				
Blakely 2013	0.29266962	0.05905912	14.6%	1.34 [1.19, 1.50]
Boffetta 2005	0.00000001	0.17682326	8.5%	1.00 [0.71, 1.41]
Jayalekshmi 2015	-0.25360274	0.23030801	6.4%	0.78 [0.49, 1.22]
Nomura 1990	0.99325181	0.21000008	7.1%	2.70 [1.79, 4.07]
Sasazuki 2002	0.53062826	0.17682326	8.5%	1.70 [1.20, 2.40]
Steevens 2010	0.57941842	0.13298391	10.6%	1.79 [1.38, 2.32]
Terry 1998	-0.18632961	0.42777529	2.6%	0.83 [0.36, 1.92]
Tran 2005	0.15614871	0.07179844	14.0%	1.17 [1.02, 1.35]
Yun 2005	0.48242617	0.06609917	14.3%	1.62 [1.42, 1.84]
Zendendel 2008	0.26236424	0.07912115	13.6%	1.30 [1.11, 1.52]
**Subtotal (95% CI)**			**100.0%**	**1.38 [1.20, 1.60]**
Heterogeneity: Tau² = 0.03; Chi² = 38.37, df = 9 (P < 0.0001); I² = 77%				
Test for overall effect: Z = 4.34 (P < 0.0001)				
**Hazard Ratio**				
Choil 2017	0.29266962	0.00955551	42.7%	1.34 [1.32, 1.37]
Eom 2015	0.24920107	0.02068993	39.1%	1.28 [1.23, 1.34]
Freedman 2007	0.81845737	0.18360378	4.2%	2.27 [1.58, 3.25]
Galanis 1998	0.09531021	0.30713591	1.6%	1.10 [0.60, 2.01]
Moy 2010	0.43825489	0.16171031	5.3%	1.55 [1.13, 2.13]
Nomura 2012	0.62486833	0.13487631	7.2%	1.87 [1.43, 2.43]
**Subtotal (95% CI)**			**100.0%**	**1.39 [1.28, 1.50]**
Heterogeneity: Tau² = 0.00; Chi² = 19.60, df = 5 (P = 0.001); I² = 74%				
Test for overall effect: Z = 8.24 (P < 0.00001)				

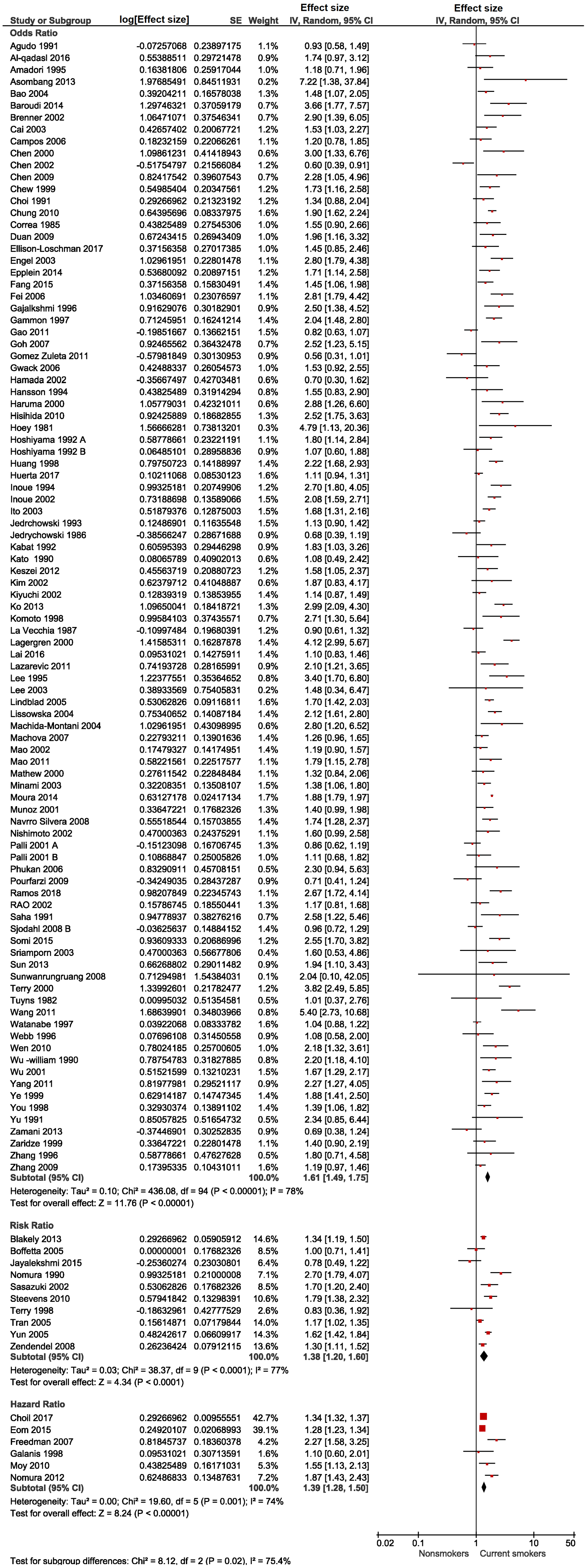

Test for subgroup differences: Chi² = 8.12, df = 2 (P = 0.02), I² = 75.4%

Study or Subgroup	log[Effect size]	SE	Weight	Effect size IV, Random, 95% CI
**Odds Ratio**				
Agudo 1991	-0.06187541	0.28493914	1.7%	0.94 [0.54, 1.64]
Amadori 1995	0.18232159	0.23374763	2.0%	1.20 [0.76, 1.90]
Brenner 2002	0.64185387	0.34056148	1.4%	1.90 [0.97, 3.70]
Campos 2006	0.40546511	0.21247681	2.2%	1.50 [0.99, 2.27]
Chen 2000	0.53062826	0.33144975	1.5%	1.70 [0.89, 3.26]
Chew 1999	-0.03252321	0.21476848	2.2%	0.97 [0.64, 1.47]
Choi 1991	-0.11653383	0.31498584	1.5%	0.89 [0.48, 1.65]
Correa 1985	0.15700372	0.34089214	1.4%	1.17 [0.60, 2.28]
Duan 2009	0.16551439	0.19537534	2.3%	1.18 [0.80, 1.73]
Ellison-Loschman 2017	0.81536484	0.22946009	2.1%	2.26 [1.44, 3.54]
Engel 2003	0.69314718	0.19442348	2.3%	2.00 [1.37, 2.93]
Epplein 2014	0.62718529	0.24478131	2.0%	1.87 [1.16, 3.03]
Gajalkshmi 1996	0.40546511	0.42464393	1.1%	1.50 [0.65, 3.45]
Gammon 1997	0.47747564	0.13742752	2.7%	1.61 [1.23, 2.11]
Gao 2011	0.08598872	0.17257901	2.5%	1.09 [0.78, 1.53]
Gwack 2006	0.19391744	0.38324562	1.2%	1.21 [0.57, 2.57]
Hamada 2002	-0.51082557	0.35364652	1.4%	0.60 [0.30, 1.20]
Hansson 1994	-0.22314353	0.29672217	1.6%	0.80 [0.45, 1.43]
Hoshiyama 1992 A	0.09531021	0.29405087	1.7%	1.10 [0.62, 1.96]
Hoshiyama 1992 B	0.39406708	0.29633847	1.6%	1.48 [0.83, 2.65]
Inoue 1994	0.94000721	0.19724232	2.3%	2.56 [1.74, 3.77]
Inoue 2002	0.43825489	0.26600301	1.8%	1.55 [0.92, 2.61]
Ito 2003	0.50077528	0.20738478	2.2%	1.65 [1.10, 2.48]
Jedrychowski 1986	-0.23572231	0.50867254	0.8%	0.79 [0.29, 2.14]
Kabat 1992	0.38594241	0.26622495	1.8%	1.47 [0.87, 2.48]
Kato 1990	0.50681758	0.46939445	0.9%	1.66 [0.66, 4.17]
Keszei 2012	0.16854116	0.19681716	2.3%	1.18 [0.80, 1.74]
Ko 2013	1.23163771	0.22899254	2.1%	3.43 [2.19, 5.37]
La Vecchia 1987	-0.19082692	0.23680156	2.0%	0.83 [0.52, 1.31]
Lagergren 2000	1.16844931	0.15303476	2.6%	3.22 [2.38, 4.34]
Lai 2016	0.18232159	0.16224204	2.5%	1.20 [0.87, 1.65]
Lee 1995	1.09861231	0.37961149	1.2%	3.00 [1.43, 6.31]
Lindblad 2005	0.26236424	0.12269711	2.8%	1.30 [1.02, 1.65]
Machida-Montani 2004	1.02961951	0.40259832	1.2%	2.80 [1.27, 6.16]
Mao 2002	0.15529287	0.10498298	2.9%	1.17 [0.95, 1.43]
Mao 2011	1.07840961	0.41437846	1.1%	2.94 [1.31, 6.62]
Minami 2003	0.15700372	0.15516524	2.6%	1.17 [0.86, 1.59]
Munoz 2001	0.18232159	0.25472674	1.9%	1.20 [0.73, 1.98]
Navrro Silvera 2008	0.36097804	0.13633981	2.7%	1.43 [1.10, 1.87]
Nishimoto 2002	0.09531021	0.31958237	1.5%	1.10 [0.59, 2.06]
Palli 1992	0.09531021	0.37796035	1.3%	1.10 [0.52, 2.31]
Palli 2001 A	-0.04775191	0.16652152	2.5%	0.95 [0.69, 1.32]
Palli 2001 B	0.03636762	0.25789106	1.9%	1.04 [0.63, 1.72]
Phukan 2006	0.58778661	0.75446707	0.4%	1.80 [0.41, 7.90]
Pourfarzi 2009	0.33647221	0.40812969	1.1%	1.40 [0.63, 3.12]
Ramon 1993	0.81093019	0.19685726	2.3%	2.25 [1.53, 3.31]
Saha 1991	0.21189651	0.31923148	1.5%	1.24 [0.66, 2.31]
Terry 2000	1.09053081	0.20348461	2.2%	2.98 [2.00, 4.43]
Wu -william 1990	0.09531021	0.37796035	1.3%	1.10 [0.52, 2.31]
Wu 2001	0.13540462	0.11181805	2.9%	1.14 [0.92, 1.43]
Ye 1999	0.18979357	0.13590091	2.7%	1.21 [0.93, 1.58]
Zaridze 1999	0.09531021	0.29405087	1.7%	1.10 [0.62, 1.96]
Zhang 1996	0.87129343	0.21311182	2.2%	2.39 [1.57, 3.63]
**Subtotal (95% CI)**			**100.0%**	**1.43 [1.29, 1.59]**
Heterogeneity: Tau² = 0.09; Chi² = 149.52, df = 52 (P < 0.00001); I² = 65%				
Test for overall effect: Z = 6.72 (P < 0.00001)				
**Risk Ratio**				
Jayalekshmi 2015	0.33647221	0.26940119	7.5%	1.40 [0.83, 2.37]
Nomura 1990	0.00000001	0.26567701	7.7%	1.00 [0.59, 1.68]
Sasazuki 2002	0.47000363	0.19902004	11.9%	1.60 [1.08, 2.36]
Steevens 2010	0.37706561	0.13837016	18.4%	1.46 [1.11, 1.91]
Tran 2005	0.05069316	0.09446895	25.4%	1.05 [0.87, 1.27]
Yun 2005	0.37156358	0.07389797	29.1%	1.45 [1.25, 1.68]
**Subtotal (95% CI)**			**100.0%**	**1.31 [1.12, 1.54]**
Heterogeneity: Tau² = 0.02; Chi² = 9.97, df = 5 (P = 0.08); I² = 50%				
Test for overall effect: Z = 3.28 (P = 0.001)				
**Hazard Ratio**				
Agudo 2012	0.19062039	0.14116971	3.2%	1.21 [0.92, 1.60]
Choil 2017	0.19885089	0.00836474	60.8%	1.22 [1.20, 1.24]
Eom 2015	0.14669438	0.03983405	25.7%	1.16 [1.07, 1.25]
Freedman 2007	0.33217734	0.14718953	3.0%	1.39 [1.04, 1.86]
Galanis 1998	0.00000001	0.34056148	0.6%	1.00 [0.51, 1.95]
Moy 2010	0.48858002	0.25758773	1.0%	1.63 [0.98, 2.70]
Nomura 2012	0.39474112	0.10422312	5.7%	1.48 [1.21, 1.82]
**Subtotal (95% CI)**			**100.0%**	**1.22 [1.16, 1.29]**
Heterogeneity: Tau² = 0.00; Chi² = 7.67, df = 6 (P = 0.26); I² = 22%				
Test for overall effect: Z = 7.77 (P < 0.00001)				

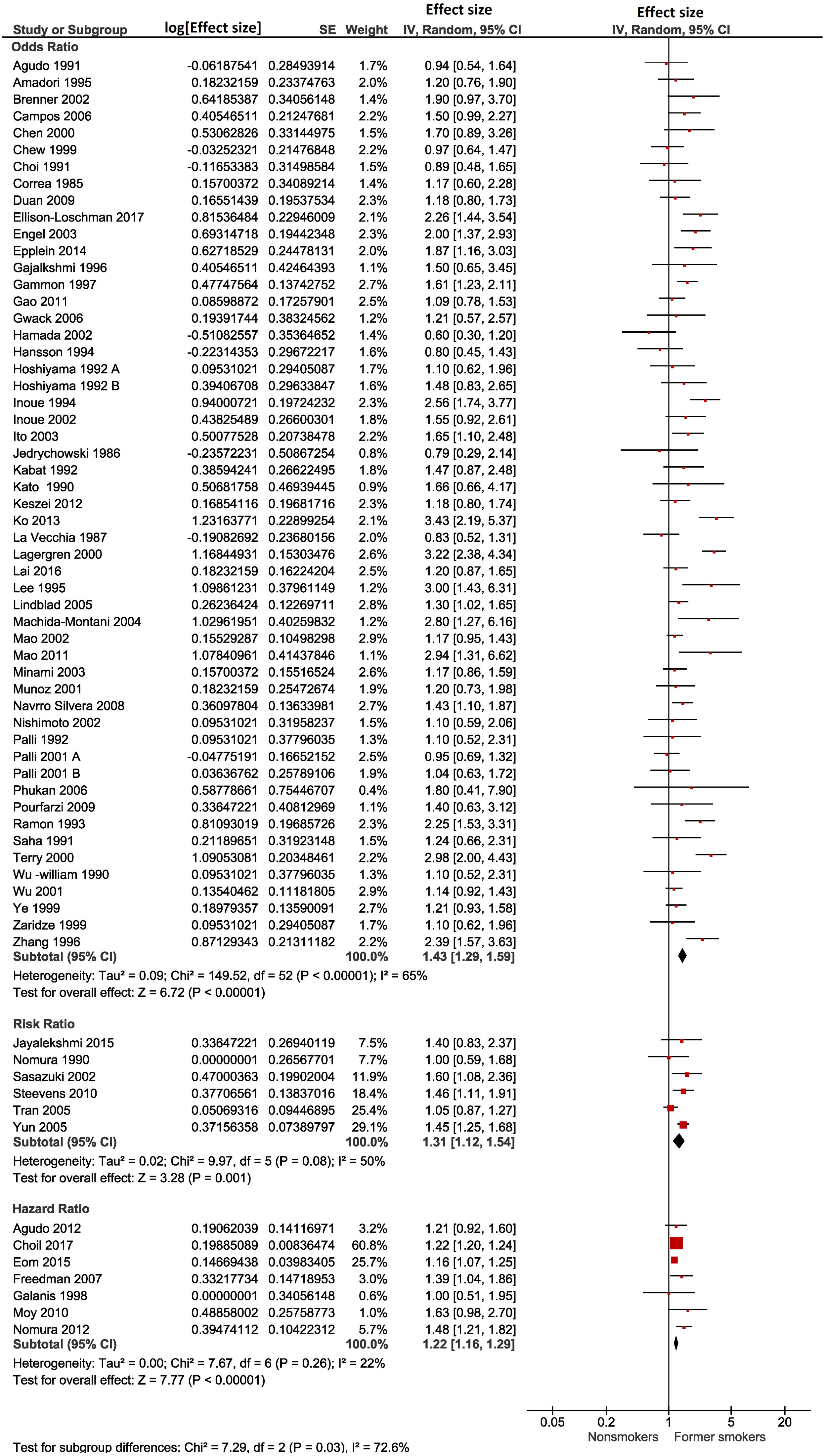

Test for subgroup differences: Chi² = 7.29, df = 2 (P = 0.03), I² = 72.6%

Study or Subgroup	log[Effect size]	SE	Weight	Effect size IV, Random, 95% CI
**Odds Ratio**				
Agudo 1991	0.17730902	0.20941983	1.5%	1.19 [0.79, 1.80]
Amadori 1995	-0.11765804	0.17725381	1.7%	0.89 [0.63, 1.26]
Bao 2004	0.13976192	0.16182749	1.8%	1.15 [0.84, 1.58]
Baroudi 2014	1.17557331	0.49762768	0.5%	3.24 [1.22, 8.59]
Behnampour 2014	0.66114038	0.71640658	0.3%	1.94 [0.48, 7.89]
Chen 2000	0.40546511	0.32359985	1.0%	1.50 [0.80, 2.83]
Chen 2002	-0.36620158	0.21495904	1.5%	0.69 [0.45, 1.06]
Chen 2009	0.53649342	0.33407158	0.9%	1.71 [0.89, 3.29]
Chew 1999	-0.62175721	0.16479126	1.8%	0.54 [0.39, 0.74]
Choi 1991	-0.15665379	0.12919937	2.0%	0.86 [0.66, 1.10]
Chung 2010	0.71539783	0.07513557	2.3%	2.05 [1.76, 2.37]
Correa 1985	0.45107564	0.17329617	1.7%	1.57 [1.12, 2.21]
D'Avanzo 1994	0.14669438	0.06806077	2.4%	1.16 [1.01, 1.32]
de Menezes 2015	0.47000363	0.04651061	2.4%	1.60 [1.46, 1.75]
Epplein 2010	-0.09193211	0.15810405	1.8%	0.91 [0.67, 1.24]
Fei 2006	0.76639761	0.24360588	1.3%	2.15 [1.34, 3.47]
Gajalkshmi 1996	-0.22314353	0.37310654	0.8%	0.80 [0.39, 1.66]
Gammon 1997	-0.36096984	1.27202621	0.1%	0.70 [0.06, 8.43]
Gao 2011	0.07603468	0.09763584	2.2%	1.08 [0.89, 1.31]
Goh 2007	0.63657683	0.52711631	0.5%	1.89 [0.67, 5.31]
Gomez Zuleta 2011	0.41871032	0.29859102	1.0%	1.52 [0.85, 2.73]
Gwack 2006	-0.04905612	0.25136566	1.3%	0.95 [0.58, 1.56]
Hamada 2002	0.17395335	0.37086937	0.8%	1.19 [0.58, 2.46]
Hansson 1994	0.02469259	0.02469259	2.5%	1.02 [0.98, 1.08]
Haruma 2000	0.59883654	0.41432247	0.7%	1.82 [0.81, 4.10]
Hoey 1981	1.94876331	0.44302765	0.6%	7.02 [2.95, 16.73]
Hoshiyama 1992 A	-0.22314353	0.24375291	1.3%	0.80 [0.50, 1.29]
Hoshiyama 1992 B	0.02078257	0.17551263	1.7%	1.02 [0.72, 1.44]
Huang 1998	0.05826885	0.11228333	2.1%	1.06 [0.85, 1.32]
Huang 2000	0.34358969	0.19724232	1.6%	1.41 [0.96, 2.08]
Inoue 1994	0.14841998	0.15191548	1.9%	1.16 [0.86, 1.56]
Jedrchowski 1993	0.74241346	0.13987768	1.9%	2.10 [1.60, 2.76]
Ji 1996	0.03922068	0.11140756	2.1%	1.04 [0.84, 1.29]
Kabat 1992	-0.45413029	0.16724086	1.8%	0.63 [0.46, 0.88]
Kato 1990	-0.31334181	0.16720544	1.8%	0.73 [0.53, 1.01]
Kim 2002	-0.61989677	0.39605352	0.7%	0.54 [0.25, 1.17]
Kiyuchi 2002	0.63657683	0.15683638	1.8%	1.89 [1.39, 2.57]
Ko 2013	0.73861426	0.17516428	1.7%	2.09 [1.48, 2.95]
Lai 2016	0.08342162	0.12625094	2.0%	1.09 [0.85, 1.39]
Lee 1995	-0.02429268	0.27196151	1.2%	0.98 [0.57, 1.66]
Lee 2003	0.18896605	0.77151453	0.2%	1.21 [0.27, 5.48]
Lin 2014	1.07158361	0.46784691	0.6%	2.92 [1.17, 7.30]
Lindblad 2005	-0.07796153	0.06688082	2.4%	0.93 [0.81, 1.05]
Mao 2002	-0.21940061	0.06487793	2.4%	0.80 [0.71, 0.91]
Mao 2011	0.65752001	0.22162749	1.4%	1.93 [1.25, 2.98]
Mathew 2000	0.40546501	0.21247681	1.5%	1.50 [0.99, 2.27]
Munoz 2001	1.06471071	0.20835745	1.5%	2.90 [1.93, 4.36]
Nakaya 2005	0.00000001	0.17682326	1.7%	1.00 [0.71, 1.41]
Nishimoto 2002	-0.61618608	0.27774489	1.1%	0.54 [0.31, 0.93]
Palli 2001 A	0.26489487	0.21711846	1.4%	1.30 [0.85, 1.99]
Palli 2001 B	0.22602579	0.33399445	0.9%	1.25 [0.65, 2.41]
Persson 2008	-0.09193747	0.13372391	2.0%	0.91 [0.70, 1.19]
Phukan 2006	0.74193728	0.29135138	1.1%	2.10 [1.19, 3.72]
Pourfarzi 2009	0.70803577	0.77858919	0.2%	2.03 [0.44, 9.34]
Ramos 2018	0.72270596	0.23257655	1.4%	2.06 [1.31, 3.25]
RAO 2002	-0.06617764	0.31104079	1.0%	0.94 [0.51, 1.72]
Sriamporn 2003	0.46436271	0.51359737	0.5%	1.59 [0.58, 4.35]
Sun 2013	0.85866159	0.30772851	1.0%	2.36 [1.29, 4.31]
Takezaki 2001	-0.21319319	1.43257131	0.1%	0.81 [0.05, 13.39]
Tuyns 1982	-0.61618608	0.61744082	0.4%	0.54 [0.16, 1.81]
Wang 2012	0.13236856	0.15865369	1.8%	1.14 [0.84, 1.56]
Watabe 1998	0.34358969	0.19228873	1.6%	1.41 [0.97, 2.06]
Webb 1996	0.14841998	0.31722033	1.0%	1.16 [0.62, 2.16]
Wu -william 1990	0.39204211	0.40400842	0.7%	1.48 [0.67, 3.27]
Yang 2011	0.87962681	0.24008797	1.3%	2.41 [1.51, 3.86]
Yang 2017	-0.10869941	0.14914961	1.9%	0.90 [0.67, 1.20]
Ye 1999	0.04401685	0.10008434	2.2%	1.04 [0.86, 1.27]
You 1998	-0.17316359	0.11856882	2.1%	0.84 [0.67, 1.06]
Yu 1991	-0.22314353	0.38369319	0.8%	0.80 [0.38, 1.70]
Zamani 2013	-0.55461156	0.54586131	0.4%	0.57 [0.20, 1.67]
Zhang 1996	-0.01106092	0.02293728	2.5%	0.99 [0.95, 1.03]
Zhang 2009	0.09166715	0.08858918	2.3%	1.10 [0.92, 1.30]
**Subtotal (95% CI)**			**100.0%**	**1.19 [1.10, 1.29]**
Heterogeneity: Tau² = 0.06; Chi² = 423.73, df = 71 (P < 0.00001); I² = 83%				
Test for overall effect: Z = 4.35 (P < 0.0001)				
**Risk Ratio**				
Jayalekshmi 2015	0.16551439	0.19871904	6.3%	1.18 [0.80, 1.74]
Sasazuki 2002	-0.03149069	0.10574063	20.1%	0.97 [0.79, 1.19]
Steevens 2010	-0.09321237	0.07298019	36.7%	0.91 [0.79, 1.05]
Stemmermann 1990	0.05354072	0.12253592	15.5%	1.05 [0.83, 1.34]
Terry 1998	0.35276732	0.20254664	6.1%	1.42 [0.96, 2.12]
Tønnesen 1994	0.00000001	0.12385402	15.2%	1.00 [0.78, 1.27]
**Subtotal (95% CI)**			**100.0%**	**1.00 [0.90, 1.10]**
Heterogeneity: Tau² = 0.00; Chi² = 5.60, df = 5 (P = 0.35); I² = 11%				
Test for overall effect: Z = 0.01 (P = 0.99)				
**3.1.3 Hazard Ratio**				
Choil 2017	0.07325044	0.00426761	53.7%	1.08 [1.07, 1.09]
Duell 2011	0.00995032	0.10206828	4.0%	1.01 [0.83, 1.23]
Eom 2015	0.11867157	0.01858581	38.7%	1.13 [1.09, 1.17]
Galanis 1998	-0.51082557	0.35364652	0.4%	0.60 [0.30, 1.20]
Ma 2015	0.19062039	0.12922479	2.6%	1.21 [0.94, 1.56]
Moy 2010	-0.04082202	0.26044932	0.7%	0.96 [0.58, 1.60]
**Subtotal (95% CI)**			**100.0%**	**1.09 [1.05, 1.14]**
Heterogeneity: Tau² = 0.00; Chi² = 9.83, df = 5 (P = 0.08); I² = 49%				
Test for overall effect: Z = 4.15 (P < 0.0001)				

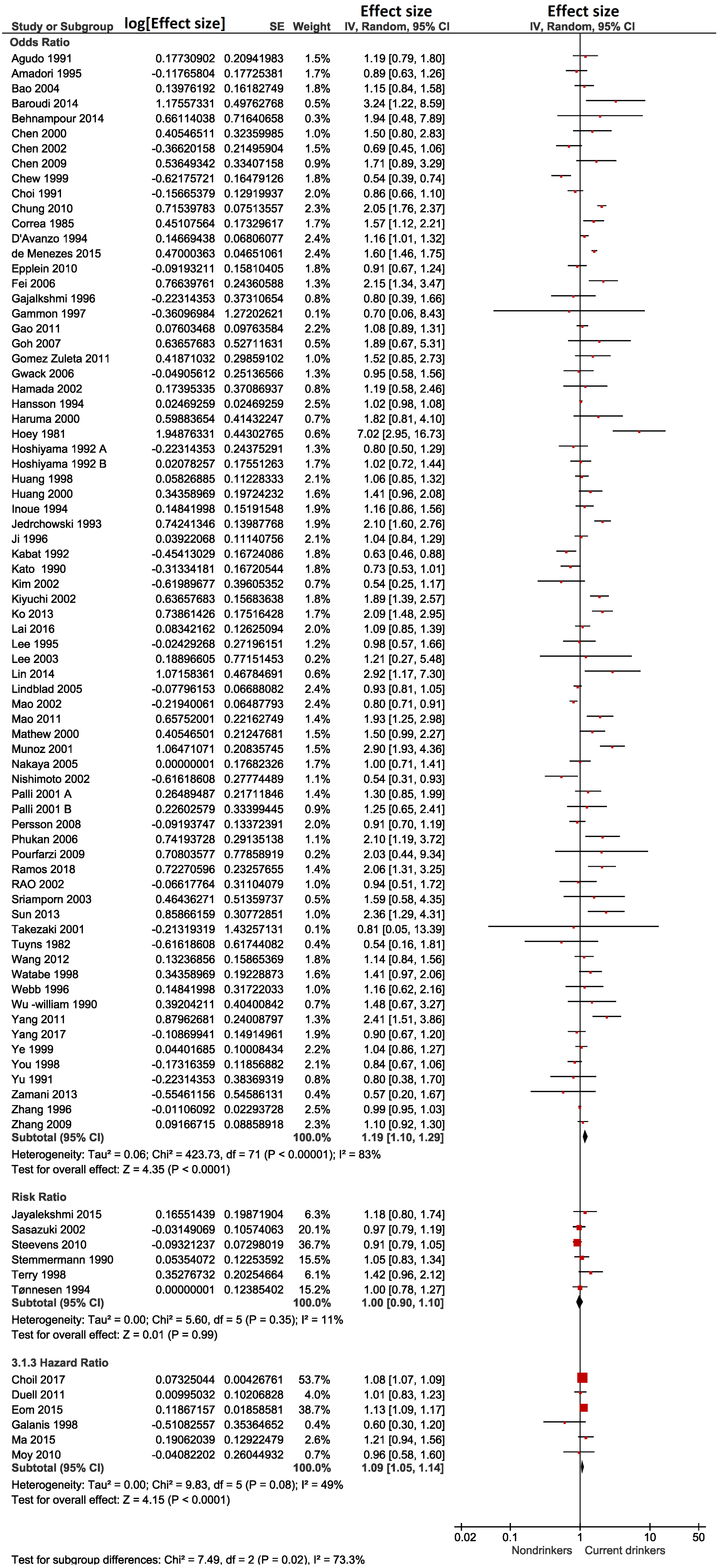

Test for subgroup differences: Chi² = 7.49, df = 2 (P = 0.02), I² = 73.3%

Study or Subgroup	log[Effect size]	SE	Weight	Effect size IV, Random, 95% CI
**Odds Ratio**				
Chew 1999	-0.42007124	0.24569163	9.1%	0.66 [0.41, 1.06]
Gajalkshmi 1996	0.33647221	0.46937793	6.7%	1.40 [0.56, 3.51]
Gwack 2006	0.39159536	0.41265193	7.3%	1.48 [0.66, 3.32]
Hoshiyama 1992 A	-0.35667497	0.45708147	6.8%	0.70 [0.29, 1.71]
Inoue 1994	0.62593842	0.26608226	8.9%	1.87 [1.11, 3.15]
Ji 1996	0.64710325	0.25484249	9.0%	1.91 [1.16, 3.15]
Ko 2013	1.20338091	0.27736139	8.8%	3.33 [1.93, 5.74]
Mao 2011	1.17557331	0.45269927	6.9%	3.24 [1.33, 7.87]
Munoz 2001	1.25276291	0.28025824	8.7%	3.50 [2.02, 6.06]
Phukan 2006	0.47000363	0.30245501	8.5%	1.60 [0.88, 2.89]
Ramos 2018	1.33762921	0.22463208	9.3%	3.81 [2.45, 5.92]
Wu 2001	-0.13696589	0.14413929	10.0%	0.87 [0.66, 1.16]
**Subtotal (95% CI)**			**100.0%**	**1.73 [1.17, 2.56]**
Heterogeneity: Tau² = 0.38; Chi² = 67.12, df = 11 (P < 0.00001); I² = 84%				
Test for overall effect: Z = 2.74 (P = 0.006)				
**Risk/Hazard Ratio**				
Duell 2011	0.29266962	0.22774494	28.0%	1.34 [0.86, 2.09]
Jayalekshmi 2015	-0.10536055	0.31218761	15.1%	0.90 [0.49, 1.66]
Ma 2015	0.37156358	0.19425163	38.1%	1.45 [0.99, 2.12]
Nakaya 2005	-0.10536055	0.28025824	18.7%	0.90 [0.52, 1.56]
**Subtotal (95% CI)**			**100.0%**	**1.21 [0.95, 1.53]**
Heterogeneity: Tau² = 0.00; Chi² = 3.08, df = 3 (P = 0.38); I² = 3%				
Test for overall effect: Z = 1.53 (P = 0.13)				

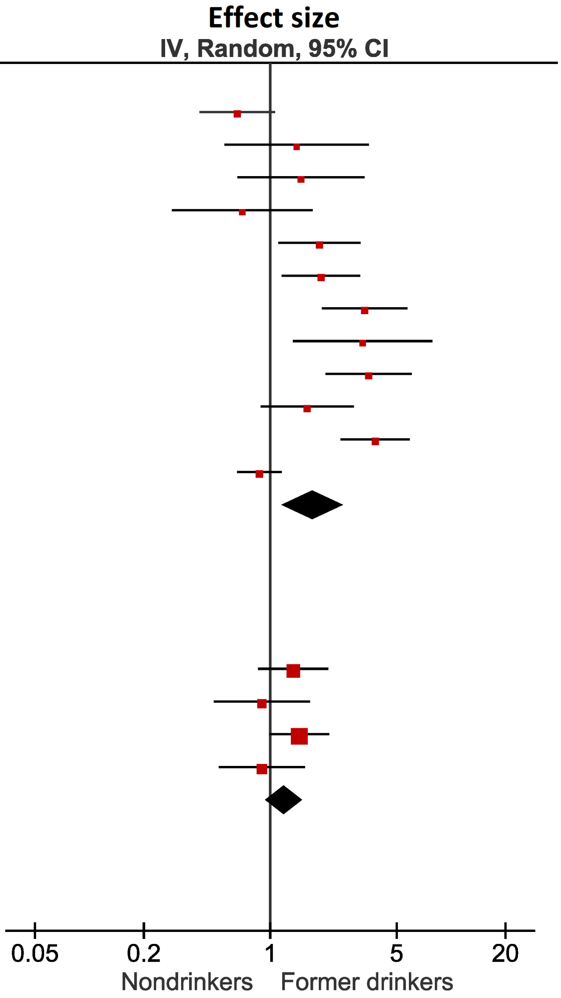

Test for subgroup differences: Chi² = 2.37, df = 1 (P = 0.12), I² = 57.7%

Study or Subgroup	log[Effect size]	SE	Weight	Effect size IV, Random, 95% CI
**Odds Ratio**				
Campbell 2007	0.06485101	0.09826515	8.3%	1.07 [0.88, 1.29]
Corley 2008	0.00598211	0.25775898	5.5%	1.01 [0.61, 1.67]
Ellison-Loschman 2017	-0.93904769	0.17976111	6.9%	0.39 [0.27, 0.56]
Fang 2015	-0.73605466	0.45031121	3.1%	0.48 [0.20, 1.16]
Hoang 2016	0.00177738	0.13303803	7.8%	1.00 [0.77, 1.30]
Huerta 2017	0.26531836	0.07695691	8.6%	1.30 [1.12, 1.52]
Kim 2014	0.17058629	0.14830826	7.5%	1.19 [0.89, 1.59]
Ko 2013	-0.41806251	0.19498309	6.6%	0.66 [0.45, 0.96]
Lindblad 2005	0.12044618	0.08283761	8.5%	1.13 [0.96, 1.33]
Machova 2007	-0.06400531	0.11447613	8.1%	0.94 [0.75, 1.17]
Sjodahl 2008 B	0.34149176	0.11781237	8.0%	1.41 [1.12, 1.77]
Sun 2013	-0.99425226	0.21614742	6.3%	0.37 [0.24, 0.57]
Wang 2012	0.27777041	0.17230986	7.1%	1.32 [0.94, 1.85]
Wen 2010	-0.38566247	0.13643822	7.7%	0.68 [0.52, 0.89]
**Subtotal (95% CI)**			**100.0%**	**0.89 [0.74, 1.08]**
Heterogeneity: Tau² = 0.10; Chi² = 90.29, df = 13 (P < 0.00001); I² = 86%				
Test for overall effect: Z = 1.18 (P = 0.24)				
**Risk/Hazard Ratio**				
Andereotti 2010	0.11955921	0.36729926	1.2%	1.13 [0.55, 2.32]
Eom 2015	-0.14041214	0.02554621	24.0%	0.87 [0.83, 0.91]
Jang 2018	0.13540462	0.10297091	9.8%	1.14 [0.94, 1.40]
Kuriyama 2005	0.06391331	0.10863317	9.1%	1.07 [0.86, 1.32]
Levi 2013	0.17311263	0.23412307	2.7%	1.19 [0.75, 1.88]
Merry 2007	-0.04082201	0.12901771	7.2%	0.96 [0.75, 1.24]
Moller 1994	0.09531021	0.11271244	8.7%	1.10 [0.88, 1.37]
Rapp 2005	-0.09431065	0.11894317	8.1%	0.91 [0.72, 1.15]
Samanic 2006	-0.18032359	0.05211747	18.4%	0.83 [0.75, 0.92]
Sojdahl 2008 A	0.02858751	0.15048561	5.7%	1.03 [0.77, 1.38]
Wolk 2001	0	0.15791816	5.3%	1.00 [0.73, 1.36]
**Subtotal (95% CI)**			**100.0%**	**0.96 [0.89, 1.04]**
Heterogeneity: Tau² = 0.01; Chi² = 18.28, df = 10 (P = 0.05); I² = 45%				
Test for overall effect: Z = 1.05 (P = 0.29)				

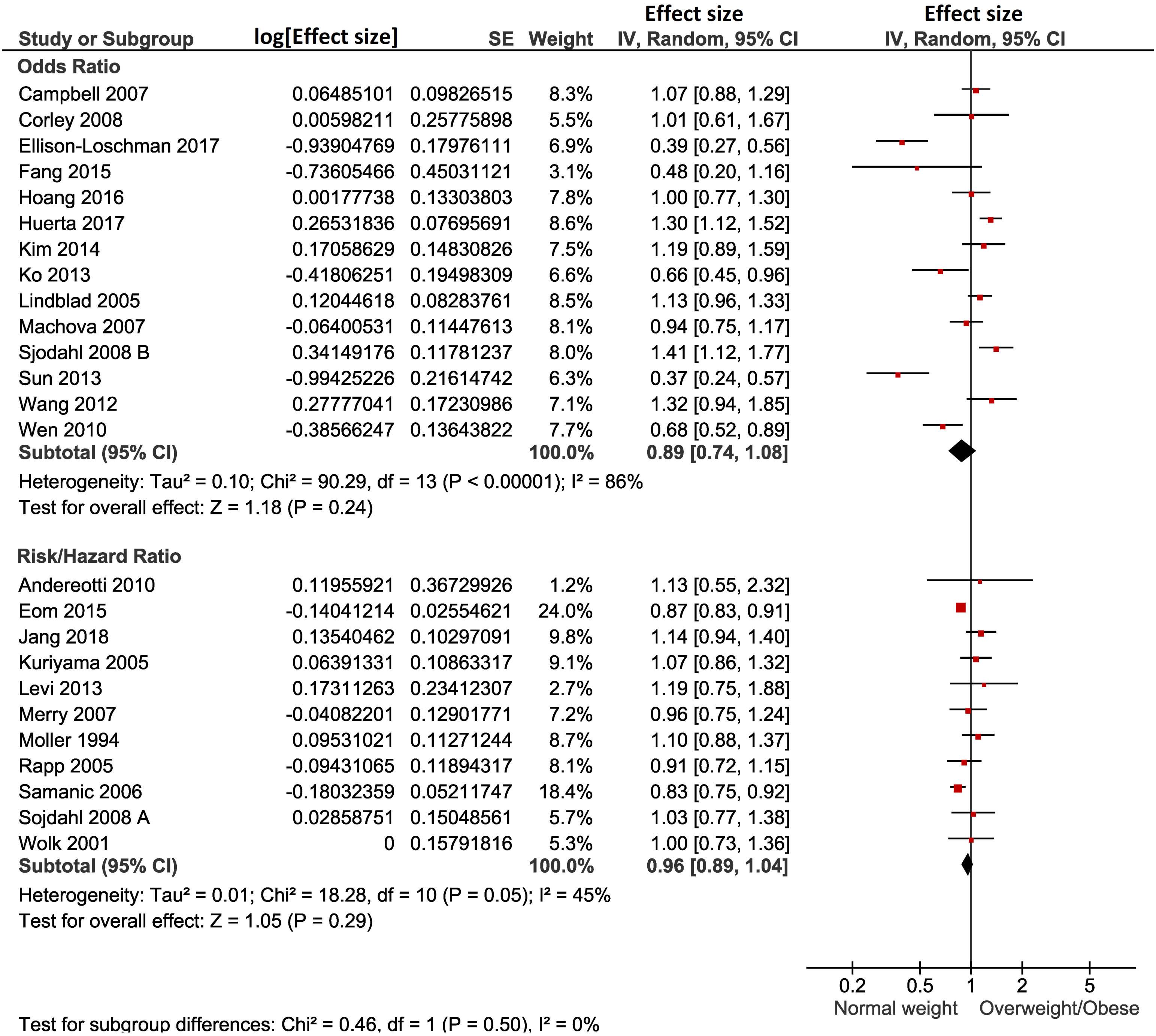

Test for subgroup differences: Chi² = 0.46, df = 1 (P = 0.50), I² = 0%

Study or Subgroup	log[Effect size]	SE	Weight	Effect size IV, Random, 95% CI
**Odds Ratio**				
Behnampour 2014	-1.56637841	0.64646137	2.4%	0.21 [0.06, 0.74]
Ellison-Loschman 2017	0.04974209	0.19824818	15.0%	1.05 [0.71, 1.55]
Gunathilake 2018	-0.47320873	0.24154425	11.8%	0.62 [0.39, 1.00]
Huerta 2017	0.09531021	0.18011494	16.6%	1.10 [0.77, 1.57]
Keszei 2012	-0.26004213	0.20273474	14.7%	0.77 [0.52, 1.15]
Parent 2010	-0.07257068	0.17104711	17.5%	0.93 [0.67, 1.30]
Sjodahl 2008 B	-0.28397045	0.13037281	21.9%	0.75 [0.58, 0.97]
**Subtotal (95% CI)**			**100.0%**	**0.83 [0.68, 1.02]**
Heterogeneity: Tau² = 0.03; Chi² = 10.93, df = 6 (P = 0.09); I² = 45%				
Test for overall effect: Z = 1.77 (P = 0.08)				
**Risk/Hazard Ratio**				
Choil 2017	-0.05837091	0.00481341	95.9%	0.94 [0.93, 0.95]
Eom 2015	-0.06081216	0.02844931	2.7%	0.94 [0.89, 0.99]
Inoue 2008	-0.10400251	0.04085814	1.3%	0.90 [0.83, 0.98]
Sojdahl 2008 A	-0.08992474	0.27213565	0.0%	0.91 [0.54, 1.56]
**Subtotal (95% CI)**			**100.0%**	**0.94 [0.93, 0.95]**
Heterogeneity: Tau² = 0.00; Chi² = 1.25, df = 3 (P = 0.74); I² = 0%				
Test for overall effect: Z = 12.53 (P < 0.00001)				

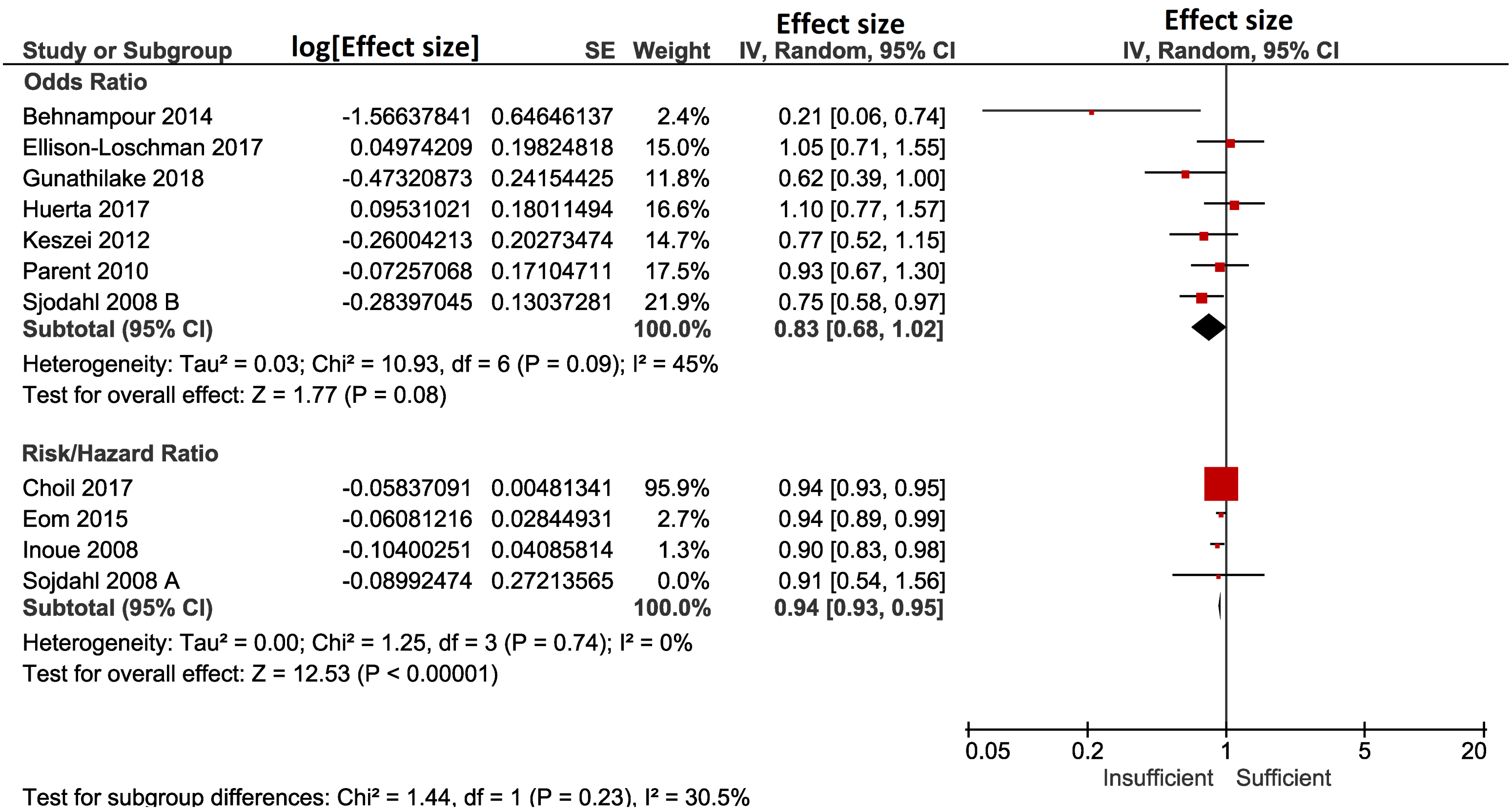

Test for subgroup differences: Chi² = 1.44, df = 1 (P = 0.23), I² = 30.5%

Study or Subgroup	log[Effect size]	SE	Weight	Effect size IV, Random, 95% CI
**Using fruit ≥3 times/week (Odds Ratio)**				
Baroudi 2014	-1.77195681	0.32359982	6.2%	0.17 [0.09, 0.32]
Cai 2003	-1.38230241	0.29539001	6.6%	0.25 [0.14, 0.45]
Demirer 1990	-2.81341081	0.37406558	5.5%	0.06 [0.03, 0.12]
Ekstrom 2000	-0.59602046	0.22346506	7.7%	0.55 [0.36, 0.85]
Gao 2011	-0.59022307	0.11017593	9.2%	0.55 [0.45, 0.69]
Inoue 1994	-0.15082288	0.11530234	9.1%	0.86 [0.69, 1.08]
La Vecchale 1997	-0.39749694	0.09209525	9.3%	0.67 [0.56, 0.80]
Lee 2002	-1.48280531	0.64891511	3.0%	0.23 [0.06, 0.81]
Lee 2003	-1.10563701	0.50218851	4.1%	0.33 [0.12, 0.89]
Mathew 2000	-0.35667497	1.30728541	0.9%	0.70 [0.05, 9.08]
Nishimoto 2002	-0.54719335	0.21775208	7.8%	0.58 [0.38, 0.89]
Pourfarzi 2009	-0.11653383	0.37360883	5.5%	0.89 [0.43, 1.85]
Ramon 1993	-0.12443006	0.23699223	7.5%	0.88 [0.55, 1.41]
Wen 2010	-0.65392649	0.18816298	8.2%	0.52 [0.36, 0.75]
Yang 2011	-0.13926207	0.08364971	9.4%	0.87 [0.74, 1.02]
**Subtotal (95% CI)**			**100.0%**	**0.48 [0.37, 0.63]**
Heterogeneity: Tau² = 0.18; Chi² = 97.43, df = 14 (P < 0.00001); I² = 86%				
Test for overall effect: Z = 5.43 (P < 0.00001)				
**Using fruit ≥3 times/week (Rate/Hazard Ratio)**				
Li 2010	-0.05657036	0.08467886	89.2%	0.94 [0.80, 1.12]
Nomura 1990	-0.22314353	0.24375291	10.8%	0.80 [0.50, 1.29]
**Subtotal (95% CI)**			**100.0%**	**0.93 [0.79, 1.09]**
Heterogeneity: Tau² = 0.00; Chi² = 0.42, df = 1 (P = 0.52); I² = 0%				
Test for overall effect: Z = 0.93 (P = 0.35)				
**Using fruit ≥7 times/week (Odds Ratio)**				
Coggon 1989	-1.60943791	0.49640563	5.0%	0.20 [0.08, 0.53]
Hamada 2002	-0.62615228	0.35367957	7.6%	0.53 [0.27, 1.07]
Hirohata 1997	-0.69314718	0.25021154	10.4%	0.50 [0.31, 0.82]
Huang 1998	-0.09431065	0.09537241	15.2%	0.91 [0.75, 1.10]
Ito 2003	-0.34249035	0.08397496	15.5%	0.71 [0.60, 0.84]
Jedrychowski 1986	-0.87923581	0.41063237	6.4%	0.42 [0.19, 0.93]
Kato 1990	-0.27443686	0.39692795	6.7%	0.76 [0.35, 1.65]
Kim 2002	-0.40047753	0.36682814	7.3%	0.67 [0.33, 1.38]
Kono 1988	-0.69314718	0.25021154	10.4%	0.50 [0.31, 0.82]
Lazarevic 2011	-1.42711641	0.27411091	9.7%	0.24 [0.14, 0.41]
Wu -william 1990	-0.40556514	0.44989762	5.7%	0.67 [0.28, 1.61]
**Subtotal (95% CI)**			**100.0%**	**0.55 [0.42, 0.71]**
Heterogeneity: Tau² = 0.11; Chi² = 35.04, df = 10 (P = 0.0001); I² = 71%				
Test for overall effect: Z = 4.56 (P < 0.00001)				
**Using fruit ≥7 times/week (Rate/Hazard Ratio)**				
Galanis 1998	-0.51082557	0.20686994	37.6%	0.60 [0.40, 0.90]
Tran 2005	-0.10981484	0.05340568	62.4%	0.90 [0.81, 0.99]
**Subtotal (95% CI)**			**100.0%**	**0.77 [0.53, 1.13]**
Heterogeneity: Tau² = 0.06; Chi² = 3.52, df = 1 (P = 0.06); I² = 72%				
Test for overall effect: Z = 1.34 (P = 0.18)				

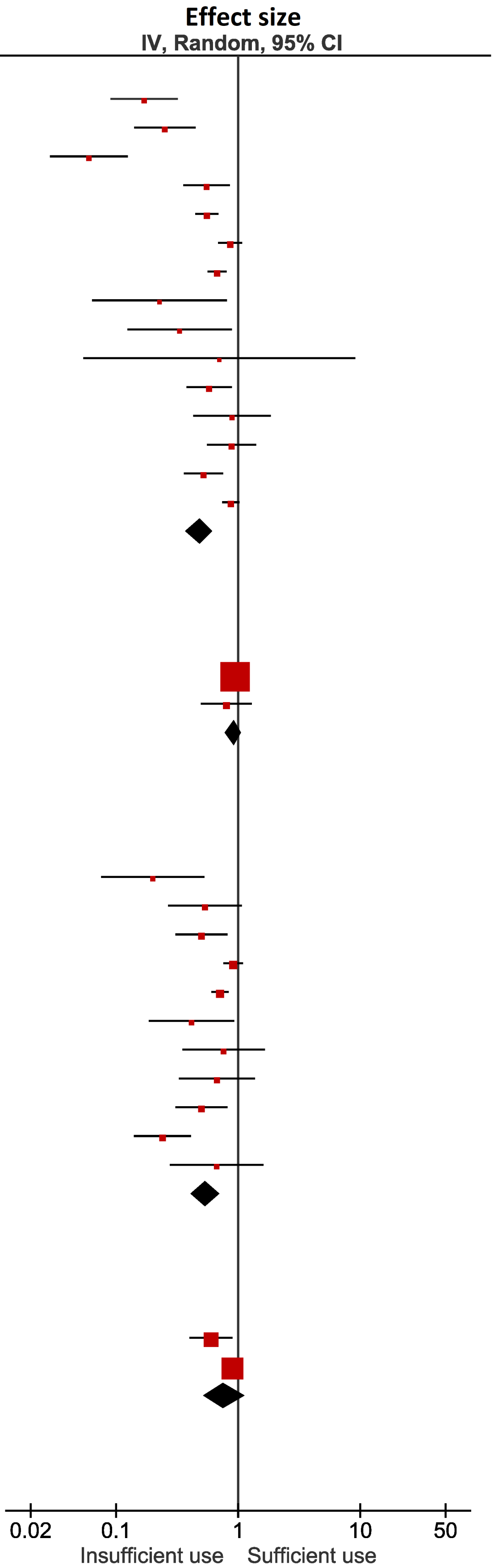

Test for subgroup differences: Chi² = 23.34, df = 3 (P < 0.0001), I² = 87.1%

Study or Subgroup	log[Odds Ratio]	SE	Weight	Odds Ratio IV, Random, 95% CI
**≥3 times/week**				
Baroudi 2014	-1.17118301	0.30863211	4.9%	0.31 [0.17, 0.57]
Ekstrom 2000	-0.78526247	0.23629573	5.8%	0.46 [0.29, 0.72]
Inoue 1994	-0.07257068	0.11344025	7.5%	0.93 [0.74, 1.16]
La Vecchale 1997	-0.41703176	0.08040263	7.8%	0.66 [0.56, 0.77]
Lee 2003	-1.60943791	0.41057089	3.7%	0.20 [0.09, 0.45]
Mathew 2000	-0.73814458	1.00878671	1.0%	0.48 [0.07, 3.45]
Nishimoto 2002	-0.29335397	0.20094205	6.3%	0.75 [0.50, 1.11]
Wen 2010	-0.40047753	0.23935449	5.8%	0.67 [0.42, 1.07]
Yang 2011	-0.08338159	0.13380727	7.2%	0.92 [0.71, 1.20]
**Subtotal (95% CI)**			**50.0%**	**0.62 [0.49, 0.79]**
Heterogeneity: Tau² = 0.08; Chi² = 30.79, df = 8 (P = 0.0002); I² = 74%				
Test for overall effect: Z = 3.82 (P = 0.0001)				
**≥7 times/week**				
Ellison-Loschman 2017	1.17118101	0.28953519	5.1%	3.23 [1.83, 5.69]
Hamada 2002	-0.18232158	0.27973291	5.2%	0.83 [0.48, 1.44]
Huang 1998	-0.22314353	0.08907761	7.7%	0.80 [0.67, 0.95]
Inoue 2002	-0.40047753	0.12348353	7.3%	0.67 [0.53, 0.85]
Ito 2003	-0.38566247	0.09728764	7.6%	0.68 [0.56, 0.82]
Jedrychowski 1986	-0.05551273	0.46811029	3.2%	0.95 [0.38, 2.37]
Kato 1990	-0.33407509	0.32395503	4.7%	0.72 [0.38, 1.35]
Kim 2002	-0.44628713	0.36959559	4.1%	0.64 [0.31, 1.32]
Sun 2013	-2.30258511	0.29405091	5.0%	0.10 [0.06, 0.18]
**Subtotal (95% CI)**			**50.0%**	**0.70 [0.48, 1.02]**
Heterogeneity: Tau² = 0.25; Chi² = 74.70, df = 8 (P < 0.00001); I² = 89%				
Test for overall effect: Z = 1.88 (P = 0.06)				
**Total (95% CI)**			**100.0%**	**0.65 [0.53, 0.80]**
Heterogeneity: Tau² = 0.14; Chi² = 105.52, df = 17 (P < 0.00001); I² = 84%				
Test for overall effect: Z = 4.04 (P < 0.0001)				
Test for subgroup differences: Chi² = 0.29, df = 1 (P = 0.59), I² = 0%				

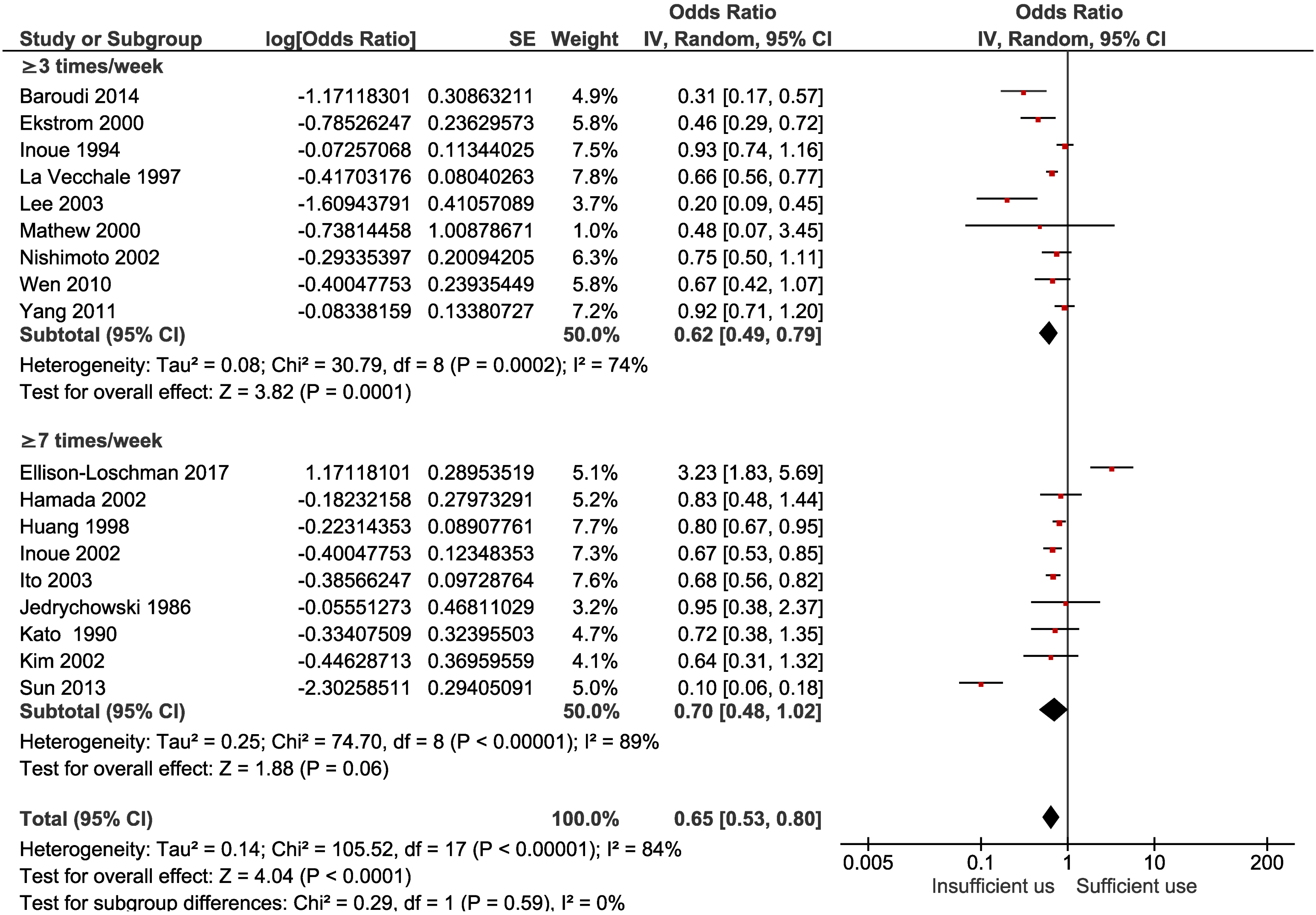

Study or Subgroup	log[Effect size]	SE	Weight	Effect size IV, Random, 95% CI
**Odds Ratio**				
Cai 2003	0.23741567	0.18216988	10.3%	1.27 [0.89, 1.81]
Gao 1999	0.21833196	0.11037521	14.8%	1.24 [1.00, 1.54]
Gao 2011	0.29266962	1.50570401	0.3%	1.34 [0.07, 25.63]
Hamada 2002	0.78115809	0.27012771	6.6%	2.18 [1.29, 3.71]
Hirohata 1997	0.41012091	0.20531249	9.1%	1.51 [1.01, 2.25]
Hoshiyama 1992 A	-0.07688104	0.28989986	5.9%	0.93 [0.52, 1.63]
Iclic 2011	0.18232159	0.26781178	6.6%	1.20 [0.71, 2.03]
Karagulle 2014	1.82272171	0.50752431	2.4%	6.19 [2.29, 16.73]
Lin 2014	0.15272105	1.39630881	0.4%	1.16 [0.08, 17.98]
Ogimoto 1995	1.38629441	1.22667461	0.5%	4.00 [0.36, 44.28]
Somi 2015	-0.12982664	0.26430658	6.7%	0.88 [0.52, 1.47]
Strumylaite 2006	-0.47803581	0.33929968	4.7%	0.62 [0.32, 1.21]
Sun 2013	0.17898265	0.12486666	13.8%	1.20 [0.94, 1.53]
Webb 1996	0.02955877	0.31891891	5.2%	1.03 [0.55, 1.92]
Wen 2010	0.32425508	0.20763668	9.0%	1.38 [0.92, 2.08]
Xibin 2002	0.55790001	0.40760353	3.5%	1.75 [0.79, 3.88]
**Subtotal (95% CI)**			**100.0%**	**1.28 [1.09, 1.51]**
Heterogeneity: $Tau^2 = 0.04$; $Chi^2 = 24.46$, df = 15 (P = 0.06); $I^2 = 39\%$				
Test for overall effect: Z = 2.95 (P = 0.003)				
**Risk Ratio**				
Galanis 1998	0.16211885	0.19676711	29.4%	1.18 [0.80, 1.73]
Nomura 1990	0.04879012	0.15952158	33.4%	1.05 [0.77, 1.44]
Tsugane 2004	0.57773632	0.12327622	37.3%	1.78 [1.40, 2.27]
**Subtotal (95% CI)**			**100.0%**	**1.32 [0.93, 1.88]**
Heterogeneity: $Tau^2 = 0.07$; $Chi^2 = 7.87$, df = 2 (P = 0.02); $I^2 = 75\%$				
Test for overall effect: Z = 1.54 (P = 0.12)				

Effect size
IV, Random, 95% CI

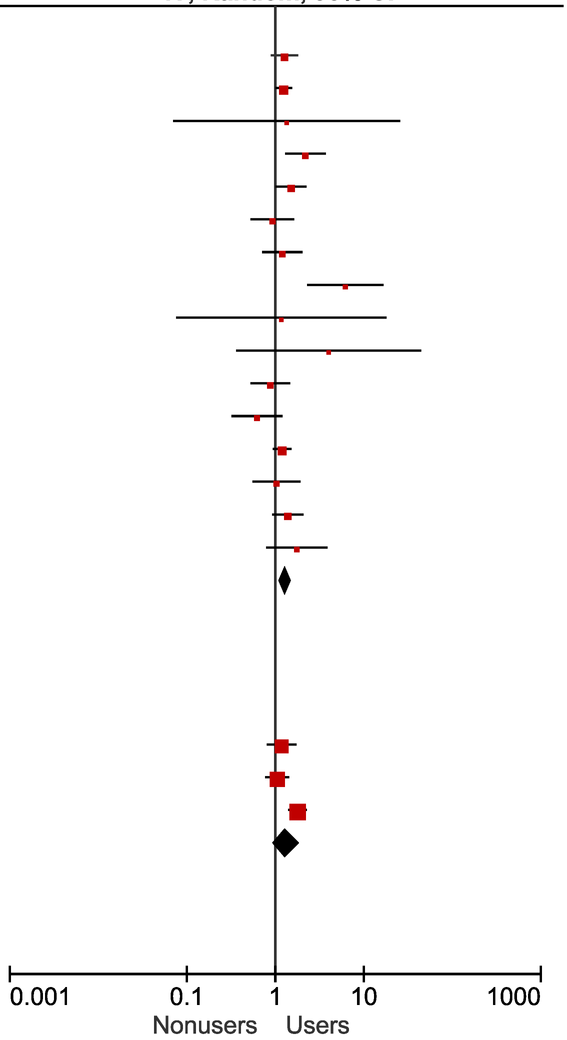

Test for subgroup differences: $Chi^2 = 0.02$, df = 1 (P = 0.88), $I^2 = 0\%$

Study or Subgroup	log[Effect size]	SE	Weight	Effect size IV, Random, 95% CI
**Odds Ratio**				
Al-qadasl 2016	-0.59783697	0.53872627	2.4%	0.55 [0.19, 1.58]
Bao 2004	-0.22815609	0.14660956	11.4%	0.80 [0.60, 1.06]
Baroudi 2014	1.06471071	0.28429955	6.2%	2.90 [1.66, 5.06]
Chen 2009	-0.37106368	0.32558504	5.2%	0.69 [0.36, 1.31]
Chew 1999	-1.24132861	1.16259681	0.6%	0.29 [0.03, 2.82]
Gallus 2009	0.02078257	0.10437228	13.4%	1.02 [0.83, 1.25]
Hansson 1993 A	-0.46203548	0.19123964	9.4%	0.63 [0.43, 0.92]
Hoshiyama 1992 A	0.23507211	0.15371795	11.0%	1.26 [0.94, 1.71]
Inoue 1994	0.53062826	0.52465373	2.5%	1.70 [0.61, 4.75]
Inoue 1998	-0.04814037	0.08230719	14.3%	0.95 [0.81, 1.12]
La Vecchia 1992	0.00000001	0.12385402	12.4%	1.00 [0.78, 1.27]
Takezaki 2001	-0.07257068	0.21354033	8.5%	0.93 [0.61, 1.41]
Watabe 1998	0.69314718	0.50026357	2.7%	2.00 [0.75, 5.33]
**Subtotal (95% CI)**			**100.0%**	**1.00 [0.84, 1.20]**
Heterogeneity: Tau² = 0.05; Chi² = 31.57, df = 12 (P = 0.002); I² = 62%				
Test for overall effect: Z = 0.04 (P = 0.97)				
**Risk/Hazard Ratio**				
Galanis 1998	-0.22314353	0.262658	22.1%	0.80 [0.48, 1.34]
Goldbohm 1996	0.04592898	0.14003634	77.9%	1.05 [0.80, 1.38]
**Subtotal (95% CI)**			**100.0%**	**0.99 [0.77, 1.26]**
Heterogeneity: Tau² = 0.00; Chi² = 0.82, df = 1 (P = 0.37); I² = 0%				
Test for overall effect: Z = 0.11 (P = 0.91)				

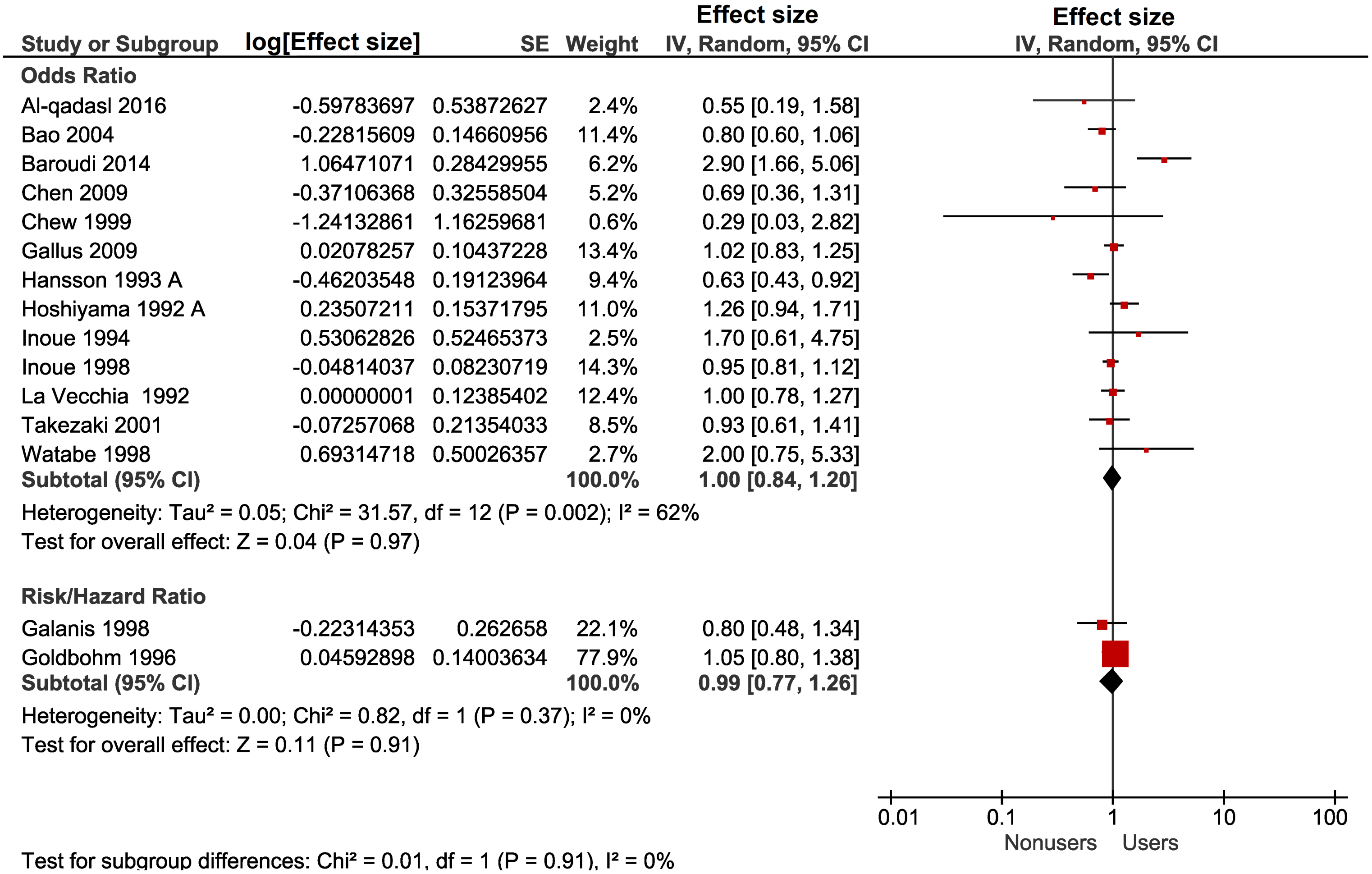

Test for subgroup differences: Chi² = 0.01, df = 1 (P = 0.91), I² = 0%

Study or Subgroup	log[Effect size]	SE	Weight	Effect size IV, Random, 95% CI
**Odds Ratio**				
Chen 2000	-0.35667497	0.28025824	2.9%	0.70 [0.40, 1.21]
Chew 1999	-0.22314353	0.18354951	6.2%	0.80 [0.56, 1.15]
Hoshiyama 2004	-0.01308525	0.21014473	4.9%	0.99 [0.65, 1.49]
Huang 1998	-0.04709161	0.05343554	28.1%	0.95 [0.86, 1.06]
Inoue 1994	0.08617773	0.13877653	9.8%	1.09 [0.83, 1.43]
Inoue 1998	-0.15315121	0.10207377	15.1%	0.86 [0.70, 1.05]
Ji 1996	-0.04082202	0.11530234	12.8%	0.96 [0.77, 1.20]
Mao 2011	0.01980261	0.21718653	4.6%	1.02 [0.67, 1.56]
Mu 2005	-0.52763277	0.27774489	3.0%	0.59 [0.34, 1.02]
Setiawan 2001	-0.65392649	0.29999974	2.6%	0.52 [0.29, 0.94]
Wang 2015	-0.32850403	0.28551826	2.8%	0.72 [0.41, 1.26]
Watabe 1998	-0.37106368	0.16640948	7.3%	0.69 [0.50, 0.96]
**Subtotal (95% CI)**			**100.0%**	**0.88 [0.80, 0.97]**
Heterogeneity: Tau² = 0.01; Chi² = 14.15, df = 11 (P = 0.22); I² = 22%				
Test for overall effect: Z = 2.54 (P = 0.01)				
**Risk/Hazard Ratio**				
Galanis 1998	0.33647221	0.18853041	12.5%	1.40 [0.97, 2.03]
Nagano 2001	-0.02122366	0.07536642	29.8%	0.98 [0.84, 1.13]
Sasazuki 2004	-0.09211529	0.06127122	32.7%	0.91 [0.81, 1.03]
Tsubone 2001	0.18232159	0.09861248	25.1%	1.20 [0.99, 1.46]
**Subtotal (95% CI)**			**100.0%**	**1.05 [0.90, 1.23]**
Heterogeneity: Tau² = 0.02; Chi² = 8.94, df = 3 (P = 0.03); I² = 66%				
Test for overall effect: Z = 0.64 (P = 0.52)				

Effect size
IV, Random, 95% CI

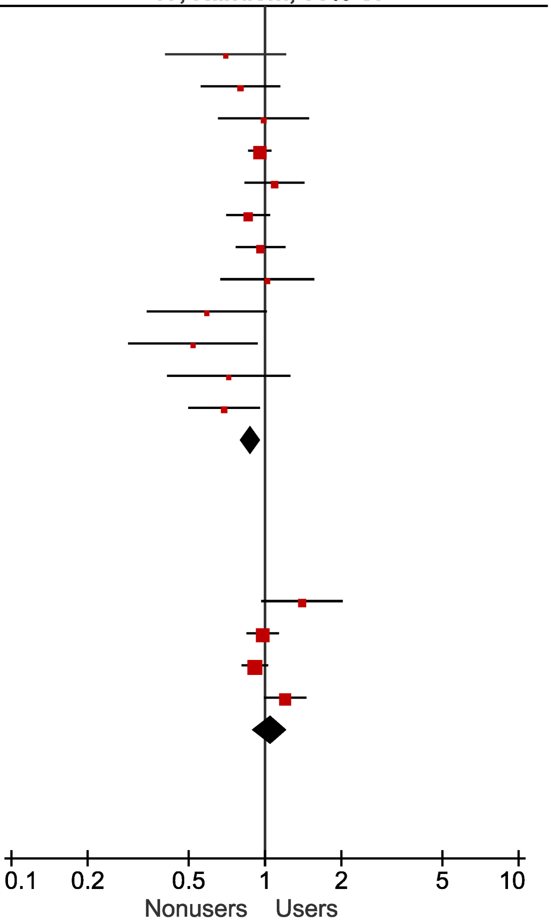

Test for subgroup differences: Chi² = 3.53, df = 1 (P = 0.06), I² = 71.7%

Study or Subgroup	log[Effect size]	SE	Weight	Effect size IV, Random, 95% CI
**Odds Ratio**				
Al-qadasl 2016	-0.77652878	0.45256631	1.7%	0.46 [0.19, 1.12]
Chen 2009	0.18232159	0.38930008	2.3%	1.20 [0.56, 2.57]
Chew 1999	0.09531021	0.20302874	7.1%	1.10 [0.74, 1.64]
Correa 1985	0.04114201	0.25229129	5.0%	1.04 [0.64, 1.71]
Gallus 2009	0.04210116	0.06868385	24.1%	1.04 [0.91, 1.19]
Gomez Zuleta 2011	0.52472854	0.30298781	3.6%	1.69 [0.93, 3.06]
Hansson 1993 A	0.26236424	0.22940801	5.8%	1.30 [0.83, 2.04]
Hansson 1993 B	0.89404511	1.62636881	0.1%	2.44 [0.10, 59.24]
Hoshiyama 1992 A	-0.30652517	0.17267518	9.1%	0.74 [0.52, 1.03]
Inoue 1994	0.13102825	0.16569556	9.7%	1.14 [0.82, 1.58]
Inoue 1998	-0.11093158	0.07696671	22.3%	0.89 [0.77, 1.04]
Watabe 1998	-0.21072103	0.17238661	9.1%	0.81 [0.58, 1.14]
**Subtotal (95% CI)**			**100.0%**	**0.99 [0.88, 1.11]**
Heterogeneity: Tau² = 0.01; Chi² = 15.53, df = 11 (P = 0.16); I² = 29%				
Test for overall effect: Z = 0.23 (P = 0.82)				
**Risk/Hazard Ratio**				
Galanis 1998	0.58778661	0.24198954	37.5%	1.80 [1.12, 2.89]
Larsson 2006	0.46184546	0.18738572	62.5%	1.59 [1.10, 2.29]
**Subtotal (95% CI)**			**100.0%**	**1.66 [1.24, 2.22]**
Heterogeneity: Tau² = 0.00; Chi² = 0.17, df = 1 (P = 0.68); I² = 0%				
Test for overall effect: Z = 3.44 (P = 0.0006)				

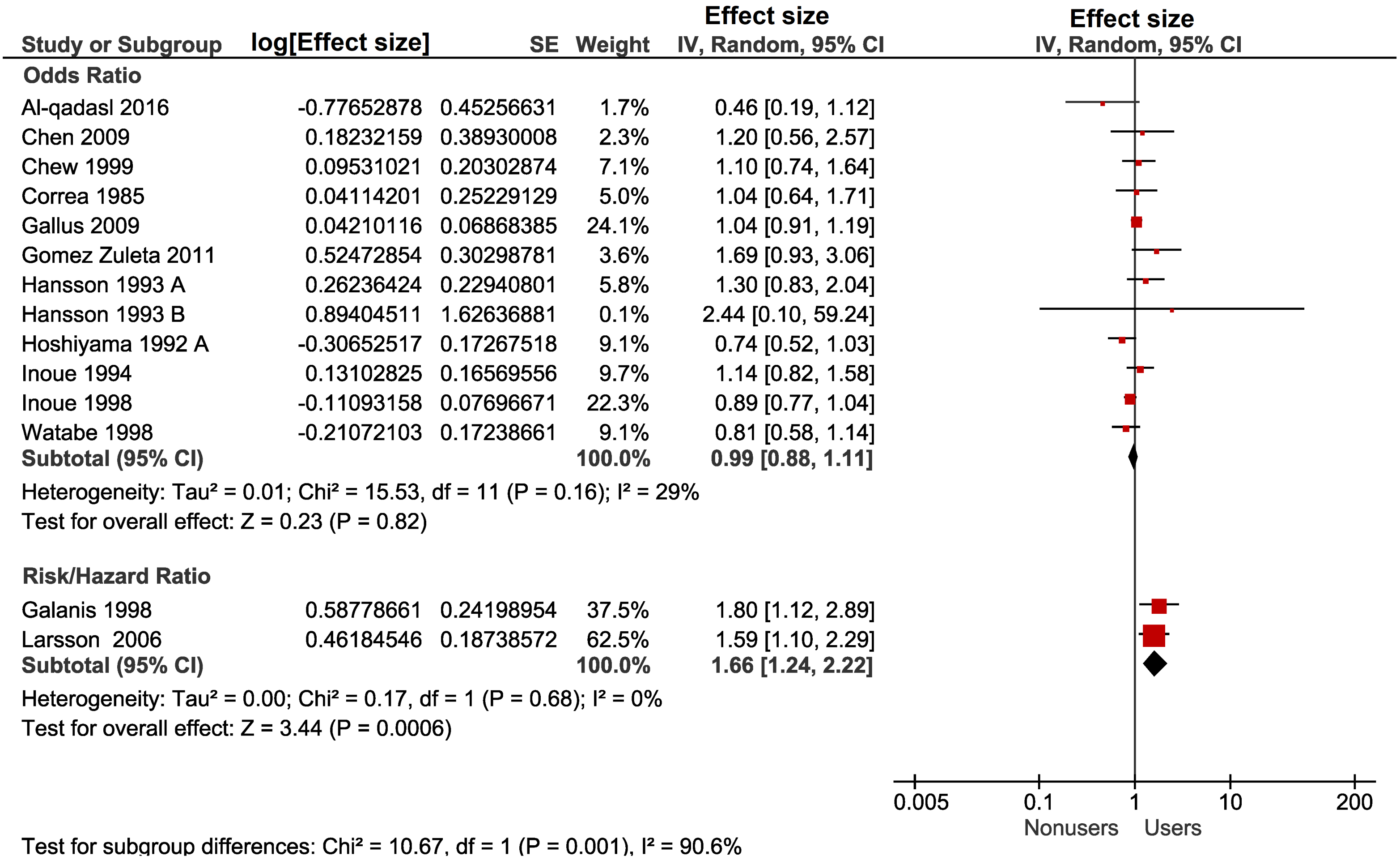

Test for subgroup differences: Chi² = 10.67, df = 1 (P = 0.001), I² = 90.6%

Study or Subgroup	log[Odds Ratio]	SE	Weight	Odds Ratio IV, Random, 95% CI
Baroudi 2014	-1.10866261	0.31135258	8.3%	0.33 [0.18, 0.61]
Ellison-Loschman 2017	0.58723098	0.21797319	10.7%	1.80 [1.17, 2.76]
Franandez 1999	-0.25360274	0.04777572	14.7%	0.78 [0.71, 0.85]
Hamada 2002	-0.66350102	0.31094739	8.3%	0.52 [0.28, 0.95]
Kim 2002	-0.24846141	0.35364652	7.3%	0.78 [0.39, 1.56]
Mathew 2000	-0.22314353	0.36911198	7.0%	0.80 [0.39, 1.65]
Nishimoto 2002	-0.15647192	0.19509415	11.4%	0.86 [0.58, 1.25]
Phukan 2006	-1.15518261	1.41198191	0.8%	0.32 [0.02, 5.01]
Pourfarzi 2009	-0.99425226	0.33266741	7.8%	0.37 [0.19, 0.71]
Strumylaite 2006	-0.19237191	0.16371463	12.2%	0.82 [0.60, 1.14]
Ward 1999	0.42526773	0.18930665	11.5%	1.53 [1.06, 2.22]
**Total (95% CI)**			**100.0%**	**0.79 [0.61, 1.03]**

Heterogeneity: Tau² = 0.12; Chi² = 42.00, df = 10 (P < 0.00001); I² = 76%

Test for overall effect: Z = 1.75 (P = 0.08)

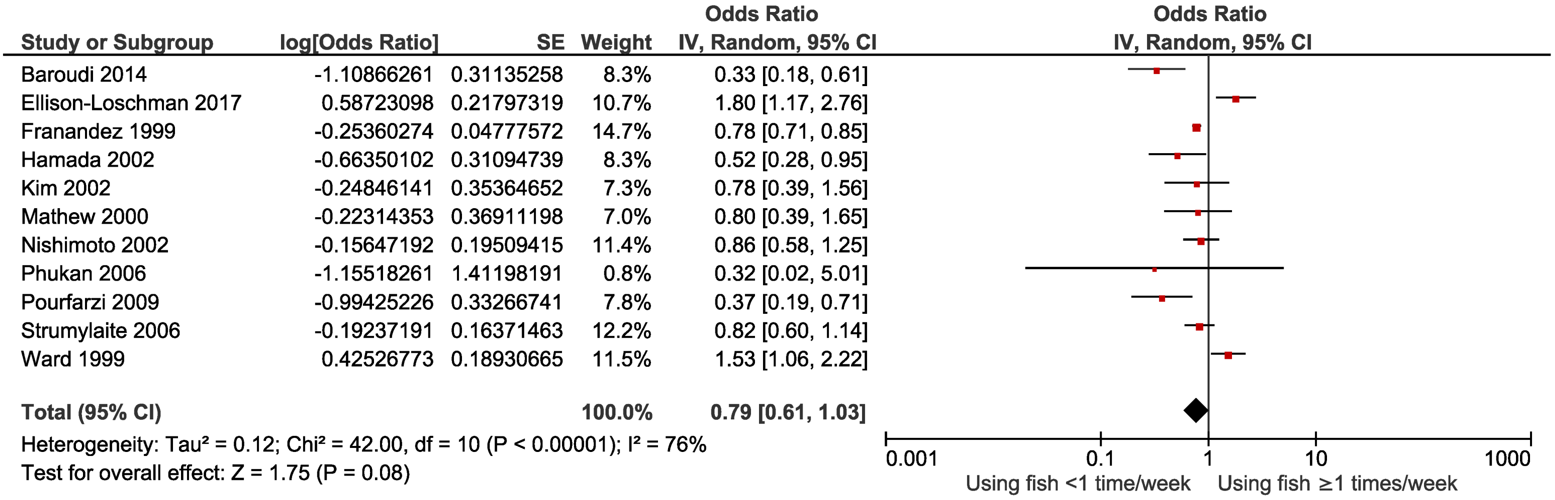

Study or Subgroup	log[Effect size]	SE	Weight	Effect size IV, Random, 95% CI
**Odds Ratio**				
Jedrychowski 1986	0.82780921	0.38583639	11.5%	2.29 [1.07, 4.87]
Kato 1990	-0.22064669	0.48128539	9.4%	0.80 [0.31, 2.06]
La Vecchale 1997	-0.04499741	0.07113382	18.7%	0.96 [0.83, 1.10]
Mathew 2000	-0.51082557	0.25806147	14.7%	0.60 [0.36, 0.99]
Nishimoto 2002	0.04329701	0.24099424	15.2%	1.04 [0.65, 1.67]
Pourfarzi 2009	0.90906471	0.25127226	14.9%	2.48 [1.52, 4.06]
Ward 1999	0.85611606	0.22422512	15.6%	2.35 [1.52, 3.65]
**Subtotal (95% CI)**			**100.0%**	**1.31 [0.87, 1.96]**
Heterogeneity: Tau² = 0.22; Chi² = 35.35, df = 6 (P < 0.00001); I² = 83%				
Test for overall effect: Z = 1.29 (P = 0.20)				
**Risk/Hazard Ratio**				
Eom 2015	-0.02839947	0.02942706	46.4%	0.97 [0.92, 1.03]
Kim 2010	-0.01005033	0.03577281	31.4%	0.99 [0.92, 1.06]
Nomura 1990	-0.22314353	0.24375291	0.7%	0.80 [0.50, 1.29]
Tran 2005	-0.06827883	0.04325766	21.5%	0.93 [0.86, 1.02]
**Subtotal (95% CI)**			**100.0%**	**0.97 [0.93, 1.01]**
Heterogeneity: Tau² = 0.00; Chi² = 1.71, df = 3 (P = 0.63); I² = 0%				
Test for overall effect: Z = 1.62 (P = 0.10)				

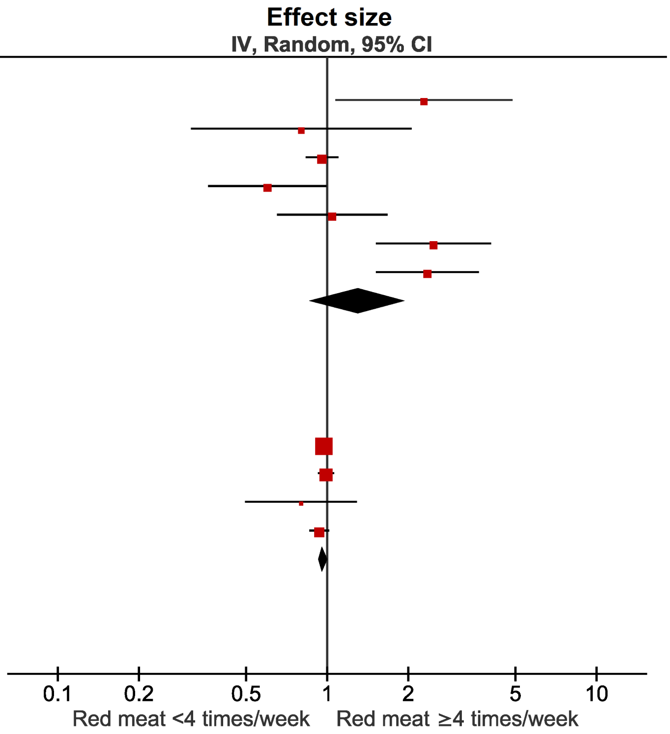

Test for subgroup differences: Chi² = 2.07, df = 1 (P = 0.15), I² = 51.6%
